# A Modular Strategy for the Synthesis of Dothideopyrones E and F, Secondary Metabolites from an Endolichenic Fungus

**DOI:** 10.1021/acs.jnatprod.2c00991

**Published:** 2023-03-31

**Authors:** Marius Aursnes, Karoline Gangestad Primdahl, David Liwara, Eirik Johansson Solum

**Affiliations:** † Department of Chemistry, Biotechnology and Food Science, Norwegian University of Life Sciences, P.O. Box 5003, NO-1433 Ås, Norway; ‡ Department of Pharmacy, Section for Pharmaceutical Chemistry, University of Oslo, P.O. Box 1068, 0316 Oslo, Norway; § Department of Chemistry, Faculty of Natural Sciences, Norwegian University of Science and Technology, NO-7491 Trondheim, Norway; ∥ Ecole Centrale de Marseille, 13013 Marseille, France; ⊥ Faculty of Health Sciences, Nord University, 8026 Bodø, Norway

## Abstract

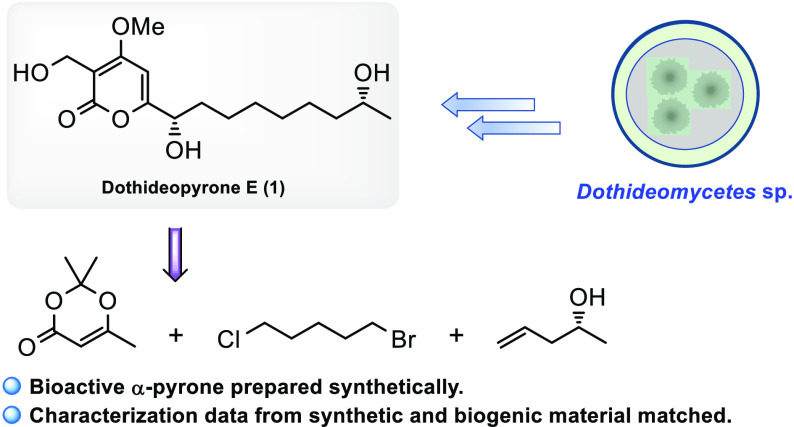

Endolichenic fungi are a rich source of natural products with a wide range of potent bioactivities. Herein, syntheses of the two naturally occurring α-pyrones dothideopyrone E and F are presented. These natural products were isolated from a culture of the endolichenic fungus *Dothideomycetes* sp. EL003334. The outlined strategy includes a Fu–Suzuki akyl–alkyl cross-coupling, a MacMillan α-oxyamination, and a Sato’s pericyclic cascade process to construct the 4-hydroxy-2-pyrone ring system. All the obtained data on the synthesized compounds matched with that of the isolated material.

Natural products from endolichenic fungi have attracted attention due to their bioactivity and new structural motifs. Several structural classes including alkaloids, steroids, peptides, and pyrones are represented and have found applications in agrochemical and pharmaceutical industries.^1^ Dothideopyrones E (**1**) and F (**2**) are recent examples of natural products isolated from cultures of the endolichenic fungus *Dothideomycetes* sp. EL003334, obtained from the lichen *Stereocaulon tomentosum*.^2^ Together with dothideopyrones A (**3**), B (**4**), C (**5**), and D (**6**), dothideopyrones E (**1**) and F (**2**) belong to a small class of naturally occurring α-pyrones.^3^ The structures of these compounds are characterized by a 4-methoxy-2-pyrone core substituted with a hydroxymethyl group at C-3 and an aliphatic side chain with one or two secondary alcohols.

Natural α-pyrones present a range of antifungal, cytotoxic, neurotoxic, and phytotoxic properties.^4^ Additionally, several naturally occurring α-pyrones have been investigated for treatment of high cholesterol and Alzheimer’s disease.^5^ Among the dothideopyrones, compound **6** displayed cytotoxic activity on cancer cell lines, while dothideopyrone F (**2**) inhibited nitric oxide (NO) and prostaglandin E_2_ (PGE_2_) production in lipopolysaccharide (LPS)-induced BV2 microglial cells.^2^ Additionally, compound **2** demonstrated the ability to decrease the transcript levels of IL-1β, IL-6, and TNF-α in a dose-dependent manner on BV2 cells stimulated with LPS. Activated microglia cells produce neuroinflammatory factors, including NO, PGE_2_, and TNF-α, as a response to danger in the central nervous system (CNS).^6^ However, uncontrolled neuroinflammatory factors contribute to neurodegeneration, leading to changes in the CNS and contribute to diseases such as Alzheimer’s and Parkinson’s disease.^7^ Hence, the control of neuroinflammation is a suitable pharmacologic target for neurodegenerative disease.^8^ In this context, the dothideopyrones, and especially dothideopyrone F (**2**), have been highlighted as a promising therapeutic lead agent to prevent neurodegenerative diseases. Owing to our interest in naturally occurring compounds, especially related to anti-inflammatory properties, this class of α-pyrones attracted our attention. Herein we present our synthetic effort to synthesize dothideopyrones E (**1**) and F (**2**).

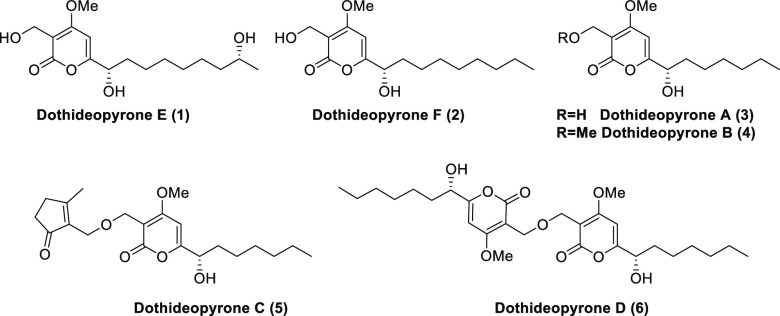



## Results and Discussion

**Scheme 1:**
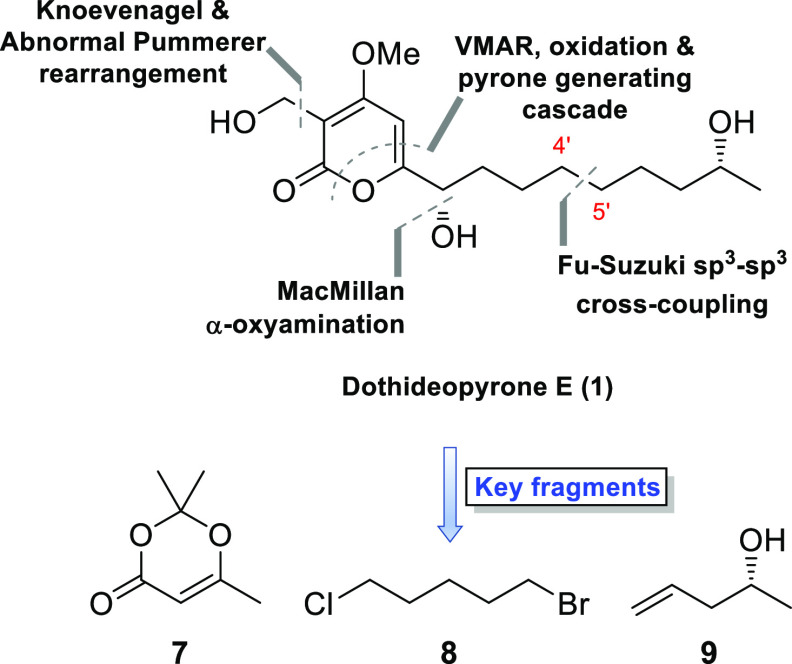
Overview of the Key Retrosynthetic Disconnections Made for Dothideopyrone E (**1**)

The retrosynthetic analysis applied to the structure of dothideopyrone E (**1**) is outlined in Scheme 1. The 4-hydroxy-2-pyrone ring system was planned to be constructed using the pericyclic cascade approach developed by Sato,^9^ allowing, thereafter, the attachment of the needed substitutions onto the α-pyrone system. For the introduction of the secondary alcohol adjacent to the pyrone ring, the enantioselective, organocatalytic α-oxyamination developed by the MacMillan group was deemed ideal.^10^ The Fu–Suzuki cross-coupling^11,12^ was chosen to forge the indicated 4′–5′ carbon–carbon bond, with readily available (*R*)-(−)-4-penten-2-ol (**9**) planned transformed into the organoborane component to be reacted with 1-bromo-5-chloropentane (**8**).

The synthesis commenced with hydroboration of TBS (*tert*-butyldimethylsilyl eter)-protected **10**,^13^ which was thereafter coupled with alkyl bromide **8**. Initially, the general procedure developed by the Fu group was applied.^11,12^ This entailed the use of 1.2 equiv of the organoboron compound and 4 mol % Pd(OAc)_2_, 8 mol % PCy_3_, and K_3_PO_4_·H_2_O in tetrahydrofuran (THF).^11^ While these standard conditions worked well, the homocoupled biproduct formed as a result of the initial Pd^2+^ → Pd^0^ reduction by the organoborane derived from **10** proved difficult to remove from the desired product **11**, thereby complicating the purification process. Additionally, it was also deemed desirable to avoid using a 20% excess of the comparably more valuable enantioenriched fragment in this specific case.

One established alternative is the application of Pd(PCy_3_)_2_, which has been found to be comparable in effectiveness to the above-mentioned catalytic system, and this approach has previously been applied in the context of synthesis of bioactive natural products.^14^ The Pd(PCy_3_)_2_ catalyst is air-sensitive and quite labile, however, and hence typically requires handling in a glovebox for optimal results. After some experimentation, we settled on using 4 mol % of the easily handled Buchwald fourth-generation^15,16^ PCy_3_-Pd-G4 as well as 4 mol % of HPCy_3_·BF_4_,^17^ with the reasoning that this should furnish Pd(PCy_3_)_2_*in situ* under the basic conditions and, importantly, without the formation of the undesired byproduct given the activation mechanism for the palladacycle precatalyst. This approach led to the smooth union of the two fragments and furnished the coupled product **11** in 77% yield. Next, using the Kornblum oxidation,^18^ the alkyl chloride functionality was transformed into the corresponding aldehyde **12** in 69% yield by simply heating **11** to 115–120 °C in DMSO together with NaHCO_3_ and NaI.

The resulting aldehyde **12** was then subjected to the MacMillan α-oxyamination conditions using 10 mol % d-proline and nitrosobenzene in CHCl_3_.^19^ In the telescoped sequence,^20^ this was followed by two reduction steps employing NaBH_4_ and Zn/AcOH before purification using column chromatography, giving the 1,2-diol **13** in 75% yield and 56:1 d.r. (HPLC analysis).

**Scheme 2:**
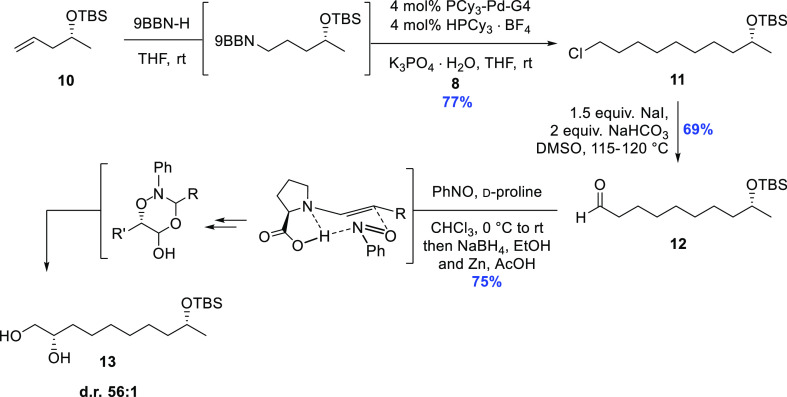
Application of the sp^3^–sp^3^ Fu–Suzuki Coupling, Kornblum Oxidation, and MacMillan α-Oxyamination to Establish the 1,8-Relationship between the Two Secondary Hydroxyl Groups in Dothideopyrone E (**1**)

It should also be pointed out that given the formation of the dioxazinaneol intermediate depicted in Scheme 2, two equivalents of the aldehyde are effectively consumed in this reaction. Consequently, an excess of aldehyde is generally used in order to achieve full consumption of nitrosobenzene: typically three equivalents or more when the aldehyde is readily available and affordable. In this case, however, the number of equivalents was lowered to two.

At this stage, the primary alcohol in the 1,2-diol system in **13** was selectively protected as the sterically hindered pivaloyl ester, and the remaining secondary alcohol was reacted with excess TBS triflate together with a catalytic amount of DMAP (4-dimethylaminopyridine) to yield **14** in 79% yield.^19^

**Scheme 3:**
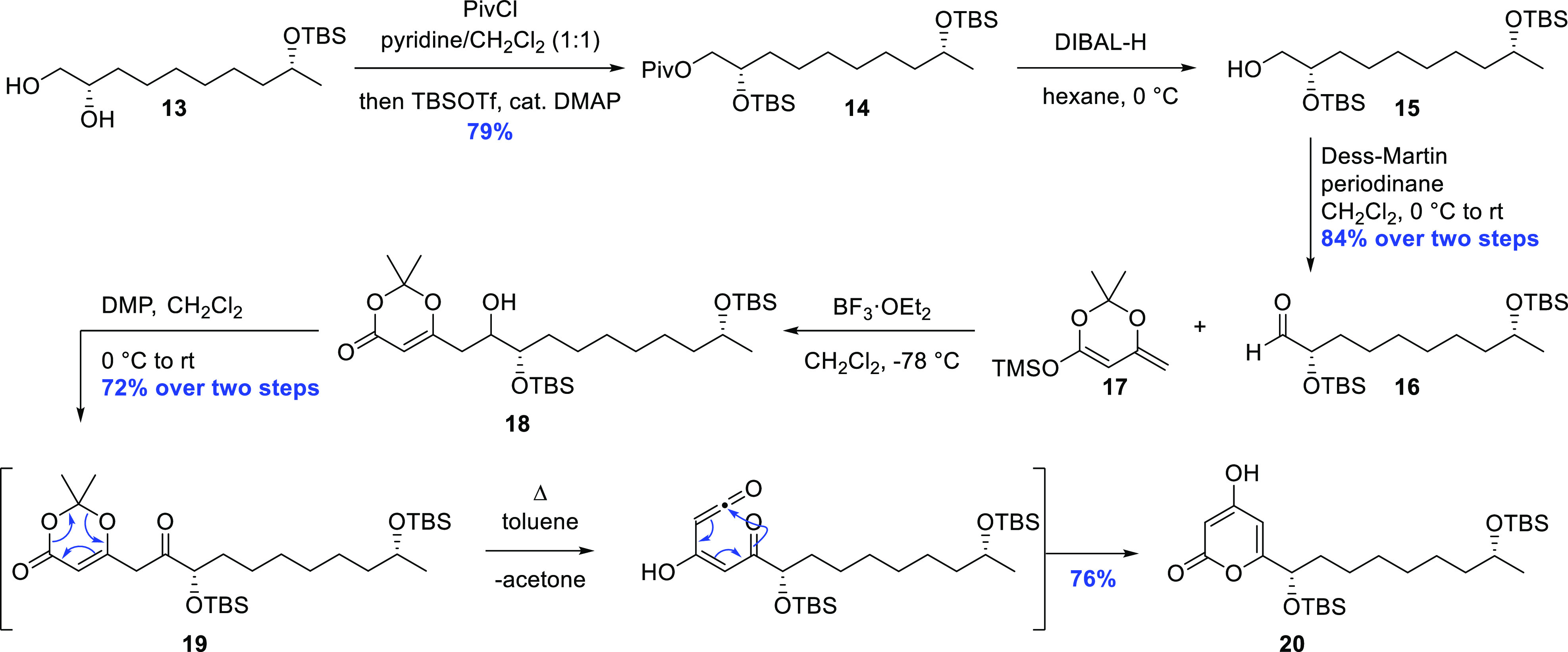
Construction of the Pyrone Nucleus Using a Pericyclic Cascade Approach

The ester moiety was then reductively cleaved with DIBAL-H (diisobutylaluminum hydride) in hexane, giving access to the primary alcohol **15**, which was subsequently oxidized to the corresponding aldehyde **16** in 84% yield over two steps. This sensitive intermediate was rapidly taken forward in a vinylogous Mukaiyama aldol reaction (VMAR) with **17** and BF_3_·OEt_2_ as the Lewis acid.^21^ To aid in the purification process, the resulting crude aldol product **18** was first oxidized, giving ketone **19** in 72% yield over two steps after column chromatography. Adding **19** dropwise to a boiling solution of toluene set in motion a retro-hetero Diels–Alder reaction, expelling acetone, followed by tautomerization and finally electrocyclization to furnish the 4-hydroxy-2-pyrone intermediate **20** in 76% yield (Scheme 3).^9^

The method of Moreno-Mañas was used for the introduction of the sulfide functionality,^22^ installed as a temporary substitute for the hydroxymethyl group attached to the 2-pyrone moiety of dothideopyrone E (**1**). Thus, by subjecting **20** to Knoevenagel conditions, employing paraformaldehyde, acetic acid, and piperidine in EtOH, a highly reactive Michael acceptor intermediate presumably forms, which is subsequently trapped by thiophenol also added to the reaction mixture.^23^ This procedure afforded the thioether product in 79% yield. Thereafter, dimethyl sulfate was used to methylate the 4-hydroxy group present in the depicted and dominant tautomer form, giving **21** in 78% yield. The next objective was to convert the thioether into the corresponding primary alcohol, and this was accomplished with the abnormal Pummerer rearrangement, which with this specific system will furnish the alcohol rather than the aldehyde functionality.^24^ The sequence was initiated by careful *m-*CBPA (*meta*-chloroperoxybenzoic acid) oxidation to prepare the sulfoxide **23**, and, after rapid purification, the obtained material was immediately treated with TFAA (trifluoroacetic anhydride). Finally, aqueous sodium hydroxide was added to hydrolyze any TFA ester formed, giving **24** in 56% yield over two steps.

**Scheme 4:**
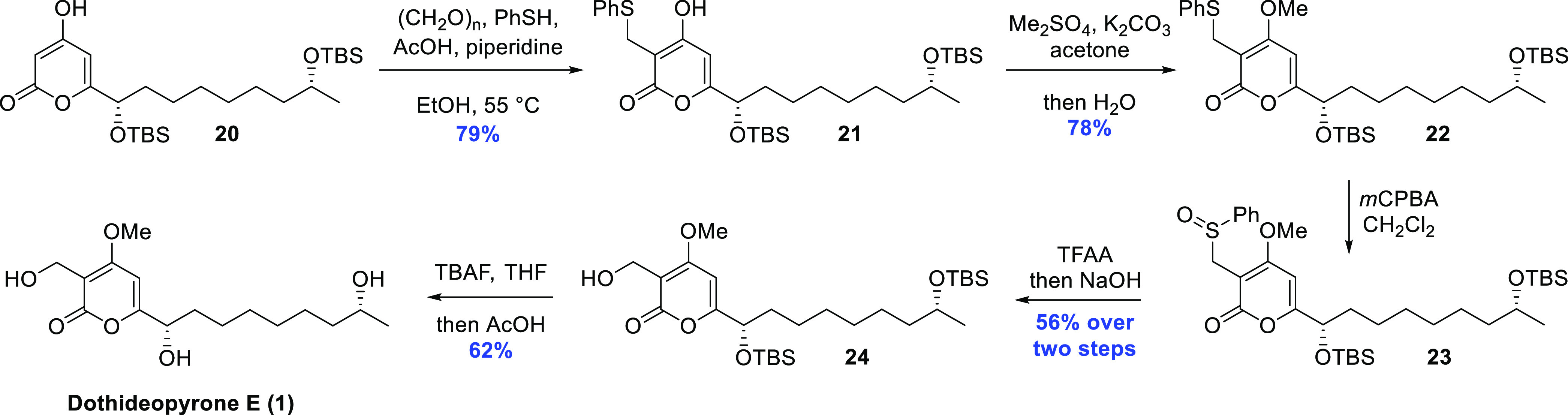
Introduction of the Hydroxymethyl Fragment, Methylation and Deprotection to Yield Dothideopyrone E (**1**)

The ultimate step involved the removal of the two TBS protecting groups. Given the highly hydrophilic nature of **1**, a procedure that avoided aqueous workup was desirable. Employing five equivalents of TBAF (tetra-*n*-butylammonium fluoride) in THF eventually led to full consumption of the starting material **24** (Scheme 4). After addition of acetic acid and removal of the solvent, the crude material was purified using column chromatography with the aim of removing as much of the tetrabutylammonium salts as possible. Fractions containing product were stored at −20 °C in order to induce precipitation of the final product. Subsequently, another round of column chromatography then afforded dothideopyrone E (**1**) in 62% yield and >96% chemical purity (Supporting Information). NMR (^1^H, ^13^C), MS, UV, and optical rotation data were all in accordance with the structure of dothideopyrone E (**1**).^2^ Furthermore, comparison between synthetic and authentic material, using the original NMR data from the isolation and characterization work, showed a clear match (Supporting Information).

**Scheme 5:**
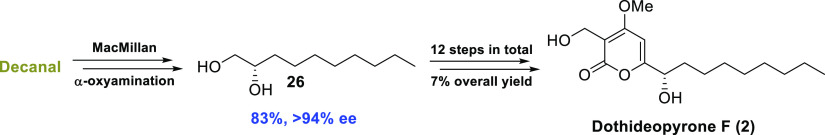
Dothideopyrone F (**2**) Was Prepared in 12 Steps Using the Established Strategy and Approach

During the course of the synthesis of dothideopyrone E (**1**), no sign of epimerization of the carbinol chiral center adjacent to the pyrone ring system was observed in any of the synthetic intermediates. This observation, coupled with the success of the synthetic approach described above, as well as the interesting biological activity of dothideopyrone F (**2**), led to the initiation of a campaign toward **2** following essentially the same strategy. The lack of a secondary alcohol in the 8′-position, however, meant that readily available and affordable decanal could be used as the starting point (Scheme 5).

The organocatalytic α-oxyamination was again used to introduce the secondary alcohol present in the 1′-position of dothideopyrone F (**2**) in 83% yield and in >94% ee. The absolute configuration was confirmed by comparison of the optical rotation value of the synthetically prepared (*S*)-decane-1,2-diol (**26**) to that of literature values (Supporting Information). From there on, the same sequence of events used to prepare dothideopyrone E (**1**) was employed to furnish dothideopyrone F (**2**) in 12 steps and 7% overall yield (Supporting Information). Also in this case, all experimental characterization data were in accordance with the structure of dothideopyrone F (**2**).^2^ Furthermore, comparison between synthetic and authentic material, using the original NMR data from the isolation and characterization work, showed a clear match (Supporting Information).

In summary, the first total syntheses of dothideopyrone E (**1**) and F (**2**) have been achieved. The key transformations include a MacMillan enantioselective and organocatalytic α-oxyamination, the Fu–Suzuki alkyl–alkyl cross-coupling reaction, and a Sato pericyclic cascade approach to build the pyrone nucleus. Regarding the Fu–Suzuki coupling, it was found that a constellation of catalytic amounts of the palladacycle precatalyst PCy_3_-Pd-G4 and additional ligand precursor HPCy_3_·BF_4_ were both convenient and effective for achieving the desired cross-coupling without the formation of a homocoupled byproduct associated with the reduction of Pd(OAc)_2_ and without the use of a glovebox.

The original elucidation work made use of the modified Mosher analysis^25^ to establish the absolute configurations of the two carbinol atoms at the 1′ and 8′ positions. In the synthetic preparation of dothideopyrone E (**1**), commercially available (*R*)-(−)-4-penten-2-ol (**10**) served as the origin for the secondary alcohol in the 8′ position, while the MacMillan α-oxyamination, with its well-established and reliable mode of stereoinduction, was used to set the absolute configuration of the carbinol atom in the 1′ position of both dothideopyrones E (**1**) and F (**2**).

Following the completion of the synthesis, specific rotation experiments showed good agreement in both sign and magnitude. The observed value for the synthetically prepared dothideopyrone E (**1**) was [α]_D_^21^ −89.9 (*c* 0.05, MeOH) compared to [α]_D_^21^ −92.68 (*c* 0.05, MeOH) for the authentic material. In the case of dothideopyrone F (**2**), the observed value was [α]_D_^21^ −109.2 (*c* 0.05, MeOH) compared to [α]_D_^21^ −118.7 (*c* 0.05, MeOH) for the authentic material. Thus, when taken as a whole, the characterization data amassed for the synthetically produced dothideopyrones corroborate the structure elucidation performed by Jang and Ahn.^2^

The established route is robust and easily amendable for future preparation of other bioactive α-pyrone natural products from this family as well as analogs. Work in this area is underway and will be reported in due course.

## Experimental Section

### General Experimental Procedures

Optical rotations were measured using a 0.7 mL cell with a 1.0 dm path length on an Anton Paar MCP 100 polarimeter. The UV/vis spectra from 190 to 900 nm were recorded using an Agilent Technologies Cary 8485 UV/vis spectrophotometer using quartz cuvettes. NMR spectra were recorded on a Bruker NEO400 or a Bruker AVIII HD 400 spectrometer at 400 MHz or a Bruker AVII600 spectrometer at 600 MHz for ^1^H NMR and at 100 or 150 MHz for ^13^C NMR. Spectra are referenced relative to the central residual solvent resonance in ^1^H NMR (CDCl_3_ δ_H_ 7.26, DMSO-*d*_6_ δ_H_ 2.50, and MeOH-*d*_4_ δ_H_ 3.31) and the central carbon solvent resonance in ^13^C NMR (CDCl_3_ δ_C_ 77.00, DMSO-*d*_6_ δ_C_ 39.52, and MeOH-*d*_4_ δ_C_ 49.00). Mass spectra were recorded at 70 eV on a Waters Prospec Q or Micromass QTOF 2W spectrometer using ESI as the method of ionization. High-resolution mass spectra were recorded at 70 eV on a Waters Prospec Q or Micromass QTOF 2W spectrometer using ESI as the method of ionization. Thin-layer chromatography was performed on silica gel 60 F254 aluminum-backed plates fabricated by Merck (Darmstadt, Germany). Flash column chromatography was performed on silica gel 60 (40–63 μm) produced by Merck (Darmstadt, Germany). Determination of enantiomeric excess was performed by HPLC on an Agilent Technologies 1200 Series instrument with a diode array detector set at the wavelength stated and equipped with a chiral stationary phase (Chiralpak AD-H, 4.6 × 250 mm, particle size 5 μm or Chiralcel OD-H, 4.6 × 250 mm, particle size 5 μm, both from Daicel Chemical Ind., Ltd.), applying the conditions stated. Achiral HPLC analyses were performed using a C18 stationary phase (Eclipse XDB-C18, 4.6 × 250 mm, particle size 5 μm, from Agilent Technologies), applying the conditions stated. Unless stated otherwise, all commercially available reagents and solvents were used in the form they were supplied without any further purification. All reactions were performed under an argon atmosphere, unless otherwise stated. The stated yields are based on isolated material. Liquid chromatography-grade solvents were purchased from Fisher Scientific (Oslo, Norway).

#### ((2,2-Dimethyl-4-methylene-4*H*-1,3-dioxin-6-yl)oxy)trimethylsilane (**17**)

Diisopropylamine (7.70 mL, 54.9 mmol, 1.00 equiv) was dissolved in THF (32.5 mL) and cooled to −78 °C. *n*-Butyllithium (2.5 M in hexane, 22.0 mL, 55.0 mmol, 1.00 equiv) was added in a dropwise manner. The resulting reaction mixture was stirred at the above-mentioned temperature for 1 h, 30 min at 0 °C and then recooled to −78 °C. Next, 2,2,6-trimethyl-4*H*-1,3-dioxin-4-one (**7**, 6.50 mL, 48.9 mmol, 0.89 equiv) was added dropwise over 30 min and stirred an additional hour. Trimethylsilyl chloride (7.60 mL, 59.9 mmol, 1.10 equiv) was then added over 30 min, and the reaction mixture was stirred for an additional 40 min. The reaction mixture was allowed to warm up to room temperature, stirred for 2 h, and then filtered through MgSO_4_ (∼5 g, dried for a few days in an exicator over 3 Å molecular sieves) under an inert atmosphere (an inverted funnel connected to an argon line was employed). The filter cake was washed with dry hexane (5 × 5.0 mL), and the filtrate was concentrated *in vacuo* (6 mbar, room temperature water-bath, argon instead of air was used to evacuate the rotary evaporator). The crude material was transferred to a flamed-dried Claisen-Vigreux under argon and distilled (oil-bath temperature ≤70 °C, 2 mbar, 44–45 °C) to afford ketene acetal **17** (7.33 g, 34.2 mmol, 70%) as a colorless oil. The spectroscopic data were in agreement with previously reported data.^26^^1^H NMR (400 MHz, CDCl_3_) δ 4.65 (s, 1H), 4.07 (d, *J* = 0.9 Hz, 1H), 3.88 (d, *J* = 0.9 Hz, 1H), 1.55 (s, 6H), 0.27 (s, 9H).

#### (*R*)-*tert*-Butyl((10-chlorodecan-2-yl)oxy)dimethylsilane (**11**)

##### Preparation of B-Alkyl-9-BBN

TBS-protected, homoallylic alcohol **10** (400 mg, 2.00 mmol, 1.05 equiv based on alkyl bromide) was added to a flame-dried flask under argon. Next, a solution of 9BBN-H (9-borabicyclo[3.3.1]nonane, 0.5 M in THF, 4.39 mL, 2.20 mmol, 1.10 equiv based on alkene) was added at room temperature, and the solution was stirred overnight.

##### Fu–Suzuki Cross-Coupling

An *undried* flask under argon was charged with PCy_3_-Pd-G4 (50.5 mg, 76.0 μmol, 4.0 mol % based on alkyl bromide), HPCy_3_·BF_4_ (28.0 mg, 76.0 μmol, 4.0 mol % based on alkyl bromide), and K_3_PO_4_·H_2_O (finely ground, 575 mg, 2.50 mmol, 1.25 equiv based on organoborane). The flask was evacuated under high vacuum and vented with argon (×3). Gentle tapping at the end ensured that no solids were attached to the sides of the flask.

Then, using the THF solution from the hydroboration described above, 1-bromo-5-chloropentane (**8**, 353 mg, 1.90 mmol, 1.00 equiv) was transferred from a flame-dried flask under argon over to the flask containing the catalyst system and base. The final reaction mixture was stirred overnight. The reaction mixture was diluted with Et_2_O (2.0 mL) and filtered through a short plug of silica gel. The obtained filtrate was concentrated *in vacuo*, and the crude material was purified with flash column chromatography (SiO_2_, heptane → 3.5% EtOAc in heptane) to yield **11** (450 mg, 1.47 mmol, 77%) as a clear oil: *R*_*f*_ (3.5% EtOAc in heptane, visualized by KMnO_4_ stain) = 0.32; [α]_D_^20^ −1.0 (*c* 1.7, CH_2_Cl_2_); ^1^H NMR (400 MHz, CDCl_3_) δ 3.81–3.72 (m, 1H), 3.53 (t, *J* = 6.8 Hz, 2H), 1.85–1.72 (m, 2H), 1.46–1.24 (m, 12H), 1.11 (d, *J* = 6.1 Hz, 3H), 0.89 (s, 9H), 0.05 (s, 3H), 0.04 (s, 3H); ^13^C{^1^H} NMR (101 MHz, CDCl_3_) δ 68.8, 45.3, 39.9, 32.8, 29.7, 29.6, 29.0, 27.0, 26.1 (3C), 25.9, 24.0, 18.3, −4.2, −4.6; HRESIMS *m*/*z* 329.2037 [M + Na]^+^ (calcd for C_16_H_35_ClNaOSi, 329.2038).

#### (*R*)-9-((*tert*-Butyldimethylsilyl)oxy)decanal (**12**)

Alkyl chloride **11** (315 mg, 1.03 mmol, 1.00 equiv) was dissolved in DMSO (2.1 mL) and NaI (finely ground, 231 mg, 1.54 mmol, 1.50 equiv) followed by the addition of NaHCO_3_ (finely ground, 172 mg, 2.05 mmol, 2.00 equiv). The flask was evacuated and filled with argon (×3), heated to 115–120 °C (oil-bath temperature), and stirred for 2 h. At this point, the flask was removed from the oil-bath and agitated in such a way as to remove the crust of salts formed on the inside wall of the flask. The flask was then placed back in the oil-bath, and the suspension was stirred until deemed complete by TLC analysis. The reaction mixture was then cooled to room temperature, diluted with H_2_O (6.5 mL), and extracted with Et_2_O (4 × 2.0 mL). The organic phase was dried (Na_2_SO_4_), filtrated, and concentrated *in vacuo*. The crude material thus obtained was purified with flash column chromatography (SiO_2_, heptane → 10% EtOAc in heptane) to yield the desired aldehyde **12**(27) (203 mg, 0.71 mmol, 69%) as a clear oil. *R*_*f*_ (10% EtOAc in heptane, visualized by KMnO_4_ stain) = 0.31; [α]_D_^25^ = +8.8 (*c* 0.6, CHCl_3_); ^1^H NMR (400 MHz, CDCl_3_) δ 9.73 (t, *J* = 1.8 Hz, 1H), 3.74 (h, *J* = 6.1 Hz, 1H), 2.38 (td, *J* = 7.4, 1.8 Hz, 2H), 1.60 (p, *J* = 7.1 Hz, 2H), 1.44–1.20 (m, 10H), 1.08 (d, *J* = 6.1 Hz, 3H), 0.86 (s, 9H), 0.01 (s, 3H), 0.01 (s, 3H); ^13^C{^1^H} NMR (101 MHz, CDCl_3_) δ 202.6, 68.7, 44.0, 39.8, 29.6, 29.4, 29.2, 26.0 (3C), 25.7, 23.9, 22.2, 18.2, −4.3, −4.6; HRESIMS *m*/*z* 309.2219 [M + Na]^+^ (calcd for C_16_H_34_NaO_2_Si, 309.2220).

#### (2*S*,9*R*)-9-((*tert*-Butyldimethylsilyl)oxy)decane-1,2-diol (**13**)

Aldehyde **12** (615 mg, 2.15 mmol, 2.00 equiv), nitrosobenzene (115 mg, 1.07 mmol, 1.00 equiv), and d-proline (12.4 mg, 0.11 mmol, 10 mol %) were combined, and the flask was cooled to 0 °C. Ice-cold CHCl_3_ (0.54 mL) was added, and the reaction was stirred at this temperature for 3 h. The reaction mixture was then transferred dropwise to a freshly made solution of NaBH_4_ (81.5 mg, 2.15 mmol, 2.00 equiv) in EtOH (6.0 mL) at 0 °C, with two EtOH washes (2 × 0.25 mL) to ensure complete transfer. The resulting reaction mixture was stirred for 3 h and then carefully concentrated *in vacuo* (prone to bumping at this stage). The crude material was treated with saturated aqueous NaHCO_3_ (∼2.5 mL) followed by extraction with EtOAc (4 × 2 mL). The combined organic phase was dried (Na_2_SO_4_), filtrated, and concentrated *in vacuo*. The product was dissolved in EtOH/AcOH (3:1, 6.0 mL), and zinc powder (702 mg, 10.7 mmol, 10.0 equiv) was added. The reaction mixture was stirred at room temperature overnight, filtrated through Celite, and concentrated *in vacuo*. The crude material thus obtained was purified with flash column chromatography (SiO_2_, 50% EtOAc in heptane) to yield diol **13** (245 mg, 0.80 mmol, 75%) as a clear oil. *R*_*f*_ (50% EtOAc in heptane, visualized by KMnO_4_ stain) = 0.17; [α]_D_^25^ 10.9 (*c* 0.6, CHCl_3_); ^1^H NMR (400 MHz, CDCl_3_) δ 3.82–3.68 (m, 2H), 3.66 (dd, *J* = 11.0, 3.1 Hz, 1H), 3.43 (dd, *J* = 11.0, 7.6 Hz, 1H), 2.29–1.99 (bs, 2H), 1.47–1.23 (m, 13H), 1.11 (d, *J* = 6.1 Hz, 3H), 0.88 (s, 9H), 0.04 (s, 3H), 0.04 (s, 3H); ^13^C{^1^H} NMR (101 MHz, CDCl_3_) δ 72.5, 68.8, 67.0, 39.8, 33.3, 29.8, 29.7, 26.1 (3C), 25.9, 25.6, 24.0, 18.3, −4.2, −4.6; HRESIMS *m*/*z* 327.2325 [M + Na]^+^ (calcd for C_16_H_36_NaO_3_Si, 327.2326).

#### (2*S*,9*R*)-2,9-Bis((*tert*-butyldimethylsilyl)oxy)decyl Pivalate (**14**)

Diol **13** (220 mg, 0.72 mmol, 1.00 equiv) was dissolved in a 1:1 mixture of CH_2_Cl_2_/pyridine (2.2 mL) and cooled to 0 °C. Then, trimethylacetyl chloride (0.11 mL, 0.86 mmol, 1.20 equiv) was added dropwise. The reaction mixture was stirred at 0 °C until deemed complete by TLC. TBSOTf (*tert*-butyldimethylsilyl trifluoromethanesulfonate, 0.41 mL, 1.80 mmol, 2.50 equiv) was then added dropwise followed by addition of one crystal of DMAP. Stirring was continued at 0 °C until deemed complete by TLC. The reaction mixture was quenched with saturated aqueous NaHCO_3_ (5.0 mL), extracted with EtOAc (3 × 4 mL), dried (Na_2_SO_4_), filtrated, and concentrated *in vacuo*. The crude material thus obtained was purified by flash chromatography (SiO_2_, heptane → 5% EtOAc in heptane) to yield **14** (288 mg, 0.57 mmol, 79%) as a clear oil. *R*_*f*_ (5% EtOAc in heptane, visualized by prolonged heating with CAM stain) = 0.36; [α]_D_^25^ −24.2 (*c* 1.0, CHCl_3_); ^1^H NMR (400 MHz, CDCl_3_) δ 3.95 (d, *J* = 5.3 Hz, 2H), 3.83 (p, *J* = 5.5 Hz, 1H), 3.76 (h, *J* = 6.1 Hz, 1H), 1.52–1.23 (m, 12H), 1.20 (s, 9H), 1.10 (d, *J* = 6.1 Hz, 3H), 0.88 (s, 18H), 0.07 (s, 3H), 0.06 (s, 3H), 0.04 (s, 3H), 0.03 (s, 3H); ^13^C{^1^H} NMR (101 MHz, CDCl_3_) δ 178.7, 70.2, 68.8, 68.3, 39.9, 38.9, 34.8, 29.9, 29.8, 27.4 (3C), 26.1 (3C), 25.9 (3C), 25.9, 25.1, 24.0, 18.3, 18.2, −4.3, −4.4, −4.5, −4.5; HRESIMS *m*/*z* 525.3765 [M + Na]^+^ (calcd for C_27_H_58_NaO_4_Si_2_, 525.3766).

#### (2*S*,9*R*)-2,9-Bis((*tert*-butyldimethylsilyl)oxy)decanal (**16**)

Pivalate **14** (280 mg, 0.56 mmol, 1.00 equiv) was dissolved in hexane (1.3 mL) and cooled to 0 °C. DIBAL-H (1.0 M in hexane, 1.39 mL, 1.39 mmol, 2.50 equiv) was added in a dropwise manner, and the reaction mixture was stirred at said temperature until deemed complete by TLC. MeOH (0.75 mL) was carefully added to quench the reaction, and then saturated aqueous potassium sodium tartrate (7.5 mL) was added. The reaction mixture was vigorously stirred, and, once the phases cleared, the aqueous phase was extracted with Et_2_O (4 × 2 mL). The combined organic phase was dried (Na_2_SO_4_), filtrated, concentrated *in vacuo*, and then kept under high vacuum for >4 h. The crude alcohol intermediate **15** was dissolved in CH_2_Cl_2_ (16 mL) and cooled to 0 °C. Then Dess–Martin periodinane reagent (283 mg, 0.67 mmol, 1.20 equiv) was added in one portion, the flask was removed from the cooling bath, and stirring was continued for 4 h. The reaction was quenched by addition of saturated aqueous Na_2_S_2_O_3_ (15 mL), and the phases were separated. The aqueous phase was extracted with CH_2_Cl_2_ (3 × 5.0 mL). The combined organic phase was dried (Na_2_SO_4_), filtrated, and concentrated *in vacuo*. The crude product thus obtained was purified by flash column chromatography (SiO_2_, 3% EtOAc in heptane) to furnish the aldehyde **16** (195 mg, 0.47 mmol, 84% over two steps) as a colorless oil. *R*_*f*_ (4% EtOAc in heptane, visualized by KMnO_4_ stain) = 0.44; [α]_D_^25^ +52.8 (*c* 0.5, CHCl_3_); ^1^H NMR (400 MHz, CDCl_3_) δ 9.59 (d, *J* = 1.7 Hz, 1H), 3.95 (ddd, *J* = 7.1, 5.5, 1.7 Hz, 1H), 3.81–3.71 (m, 1H), 1.67–1.54 (m, 2H), 1.48–1.20 (m, 10H), 1.10 (d, *J* = 6.1 Hz, 3H), 0.92 (s, 9H), 0.88 (s, 9H), 0.08 (s, 3H), 0.07 (s, 3H), 0.04 (s, 3H), 0.04 (s, 3H); ^13^C{^1^H} NMR (101 MHz, CDCl_3_) δ 204.6, 77.9, 68.8, 39.8, 32.8, 29.7, 29.6, 26.1, 26.1 (3C), 25.9 (3C), 25.8, 24.8, 24.0, 18.4, 18.3, −4.2, −4.5, −4.6, −4.8. Mass not found for this one.

#### 6-((3*S*,10*R*)-3,10-Bis((*tert*-butyldimethylsilyl)oxy)-2-oxoundecyl)-2,2-dimethyl-4*H*-1,3-dioxin-4-one (**19**)

Aldehyde **16** (337 mg, 0.81 mmol, 1.00 equiv) and freshly distilled ketene acetal **17** (433 mg, 2.02 mmol, 2.50 equiv) were dissolved in CH_2_Cl_2_ (11.5 mL) and cooled to −78 °C. Next, BF_3_·OEt_2_ (249 μL, 2.02 mmol, 2.50 equiv) was added dropwise over 30 min. The reaction mixture was stirred for 1 h and then quenched by the addition of phosphate buffer (10 mL, pH = 7) and warmed to room temperature, and then saturated aqueous NaHCO_3_ (20 mL) was added. The phases were separated, and the aqueous phase was extracted with CH_2_Cl_2_ (3 × 10 mL). The combined organic phase was dried (Na_2_SO_4_), filtrated, and concentrated *in vacuo*. The aldol product **18** coeluted (*R*_*f*_ = 0.15 in 20% EtOAc in heptane) with the hydrolyzed ketene acetal **7** (2,2,6-trimethyl-4*H*-1,3-dioxin-4-one), and the crude material was therefore directly taken up in CH_2_Cl_2_ (25 mL) and cooled to 0 °C. Next, the Dess–Martin periodinane reagent (416 mg, 0.981 mmol) and NaHCO_3_ (100 mg, 1.19 mmol) were added, and the flask was removed from the cooling bath. The reaction mixture was stirred until deemed complete by TLC analysis and quenched by the addition of saturated aqueous Na_2_S_2_O_3_ (5.0 mL), and then the phases were separated. The aqueous phase was extracted with CH_2_Cl_2_ (3 × 10 mL). The combined organic phase was dried (Na_2_SO_4_), filtrated, and concentrated *in vacuo*. The crude product thus obtained was purified by flash column chromatography (SiO_2_, 20% EtOAc in heptane) to yield ketone **19** (324 mg, 0.58 mmol, 72% over two steps) as a clear oil. *R*_*f*_ (20% EtOAc in heptane, visualized by KMnO_4_ stain) = 0.29; [α]_D_^25^ −25.0 (*c* 0.6, CHCl_3_); ^1^H NMR (400 MHz, CDCl_3_) δ 5.32 (s, 1H), 4.05 (t, *J* = 6.1 Hz, 1H), 3.76 (apparent, *J* = 6.2 Hz, 1H), 3.48 (s, 2H), 1.71 (s, 3H), 1.70 (s, 3H), 1.68–1.51 (m, 2H), 1.47–1.20 (m, 10H), 1.10 (d, *J* = 6.0 Hz, 3H), 0.93 (s, 9H), 0.88 (s, 9H), 0.08 (s, 3H), 0.06 (s, 3H), 0.04 (s, 3H), 0.03 (s, 3H); ^13^C{^1^H} NMR (101 MHz, CDCl_3_) δ 207.0, 165.5, 160.9, 107.3, 97.1, 78.9, 68.7, 41.8, 39.8, 34.9, 29.7, 29.6, 26.1 (3C), 25.9 (3C), 25.8, 25.2, 24.7, 24.0, 18.3, 18.2, −4.2, −4.5, −4.7, −4.8; HRESIMS *m*/*z* 579.3506 [M + Na]^+^ (calcd for C_29_H_56_NaO_6_Si_2_, 579.3508).

#### 4-Hydroxy-6-((5*S*,12*R*)-2,3,12,14,15-nonamethyl-4,13-dioxa-3,14-disilahexadecan-5-yl)-2*H*-pyran-2-one (**20**)

Ketone **19** (400 mg, 0.72 mmol, 1.00 equiv) was azeotroped with toluene (2 × 3 mL) and then dissolved in toluene (4.5 mL). This solution was added dropwise to a solution of boiling toluene (28 mL) over a period of 10 min. More toluene (2 × ∼0.5 mL) was used to wash the flask, and the washings were added to the refluxing reaction mixture. The solution was further refluxed for 45 min, cooled to room temperature, and concentrated *in vacuo*. The crude product thus obtained was purified by flash column chromatography (SiO_2_, 50% EtOAc in heptane, tailing was observed) to yield pyrone **20** (273 mg, 0.55 mmol, 76%) as a yellow oil. *R*_*f*_ (50% EtOAc in heptane, visualized by UV and KMnO_4_ stain) = 0.30; [α]_D_^25^ −93.3 (*c* 1.4, CHCl_3_); ^1^H NMR (400 MHz, CDCl_3_) δ 10.38 (bs, 1H), 6.23 (ad, *J* = 1.4 Hz, 1H), 5.55 (ad, *J* = 2.1 Hz, 1H), 4.43 (t, *J* = 5.3 Hz, 1H), 3.76 (ah, *J* = 6.2 Hz, 1H), 1.75–1.67 (m, 2H), 1.48–1.18 (m, 10H), 1.11 (d, *J* = 6.1 Hz, 3H), 0.92 (s, 9H), 0.88 (s, 9H), 0.08 (s, 3H), 0.04 (s, 3H), 0.04 (s, 3H), 0.03 (s, 3H); ^13^C{^1^H} NMR (101 MHz, CDCl_3_) δ 172.1, 169.3, 167.0, 99.6, 90.2, 71.0, 68.7, 39.7, 36.3, 29.6, 29.5, 25.9 (3C), 25.8 (3C), 24.3, 23.8, 18.2, 18.1, −4.4, −4.7, −4.9, −5.0; HRESIMS *m*/*z* 521.3087 [M + Na]^+^ (calcd for C_26_H_50_NaO_5_Si_2_, 521.3089).

#### 4-Hydroxy-6-((5*S*,12*R*)-2,3,12,14,15-nonamethyl-4,13-dioxa-3,14-disilahexadecan-5-yl)-3-((phenylthio)methyl)-2*H*-pyran-2-one (**21**)

Pyrone **20** (188 mg, 0.38 mmol, 1.00 equiv) was dissolved in EtOH (18 mL) and then added to a suspension consisting of paraformaldehyde (18 mg, 0.60 mmol as monomer, 1.6 equiv), thiophenol (0.3 mL, 2.93 mmol, 7.80 equiv), acetic acid (17.7 μL, 0.31 mmol, 0.82 equiv), and piperidine (17.5 μL, 0.18 mmol, 0.47 equiv) in EtOH (12 mL) at 55 °C (oil-bath temperature). The reaction was stirred for 20 h at said temperature, cooled to room temperature, and concentrated *in vacuo*. The crude product thus obtained was purified by flash column chromatography (SiO_2_, 30% EtOAc in heptane) to yield the pyrone **21** (186 mg, 0.30 mmol, 79%) as a yellow oil. *R*_*f*_ (30% EtOAc in heptane, visualized by UV and KMnO_4_ stain) = 0.37; [α]_D_^20^ −6.76 (*c* 1.0, CH_2_Cl_2_); ^1^H NMR (400 MHz, CDCl_3_) δ 8.86 (bs, 1H), 7.42–7.36 (m, 2H), 7.31–7.15 (m, 3H), 6.14 (s, 1H), 4.37 (t, *J* = 5.4 Hz, 1H), 4.09 (s, 2H), 3.77 (ah, *J* = 6.2 Hz, 1H), 1.75–1.58 (m, 2H), 1.49–1.17 (m, 10H), 1.10 (d, *J* = 6.0 Hz, 3H), 0.90 (s, 9H), 0.88 (s, 9H), 0.05 (s, 3H), 0.04 (2 × s, 6H), −0.01 (s, 3H); ^13^C{^1^H} NMR (101 MHz, CDCl_3_) δ 167.7, 167.5, 164.8, 133.5, 130.6, 129.2, 127.4, 98.9, 97.7, 71.1, 68.8, 39.8, 36.3, 29.8, 29.6, 28.5, 26.1 (3C), 25.9 (3C), 24.4, 24.0, 18.3, 18.2, −4.2, −4.5, −4.7, −4.9; HRESIMS *m*/*z* 643.3280 [M + Na]^+^ (calcd for C_33_H_56_NaO_5_SSi_2_, 643.3279).

#### 4-Methoxy-6-((5*S*,12*R*)-2,3,12,14,15-nonamethyl-4,13-dioxa-3,14-disilahexadecan-5-yl)-3-((phenylthio)methyl)-2*H*-pyran-2-one (**22**)

Pyrone **21** (165 mg, 0.27 mmol, 1.00 equiv) was dissolved in acetone (2.7 mL). Dimethyl sulfate (126 μL, 1.33 mmol, 5.00 equiv) was added followed by K_2_CO_3_ (184 mg, 1.33 mmol, 5.00 equiv). The reaction mixture was stirred for 1 h, water (1.4 mL) was added, and then the reaction mixture was vigorously stirred overnight. Saturated aqueous NH_4_Cl (4.5 mL) was then added, and the reaction mixture was extracted with CH_2_Cl_2_ (5 × 1.0 mL). The combined organic phase was dried (Na_2_SO_4_), filtrated, and concentrated *in vacuo*. The crude material thus obtained was purified by flash column chromatography (SiO_2_, 10% → 30% EtOAc in heptane) to yield pyrone **22** (132 mg, 0.21 mmol, 78%) as a clear oil. *R*_*f*_ (30% EtOAc in heptane, visualized by UV and KMnO_4_ stain) = 0.49; [α]_D_^20^ −78.4 (*c* 1.0, CH_2_Cl_2_); ^1^H NMR (400 MHz, CDCl_3_) δ 7.49–7.41 (m, 2H), 7.30–7.23 (m, 2H), 7.22–7.17 (m, 1H), 6.29 (s, 1H), 4.45 (dd, *J* = 6.4, 4.2 Hz, 1H), 3.98 (s, 2H), 3.77 (h, *J* = 6.2 Hz, 1H), 3.70 (s, 3H), 1.80–1.63 (m, 2H), 1.48–1.22 (m, 10H), 1.11 (d, *J* = 6.0 Hz, 3H), 0.94 (s, 9H), 0.89 (s, 9H), 0.11 (s, 3H), 0.05 (s, 3H), 0.04 (s, 3H), 0.04 (s, 3H); ^13^C{^1^H} NMR (101 MHz, CDCl_3_) δ 168.7, 167.3, 163.8, 136.5, 131.6, 128.7, 126.7, 101.7, 92.4, 71.4, 68.7, 56.4, 39.8, 36.6, 29.7, 29.6, 28.6, 26.1 (3C), 25.9 (3C), 24.6, 24.0, 18.3, 18.3, −4.3, −4.5, −4.8, −4.8; HRESIMS *m*/*z* 657.3435 [M + Na]^+^ (calcd for C_34_H_58_NaO_5_SSi_2_, 657.3436).

#### 3-(Hydroxymethyl)-4-methoxy-6-((5*S*,12*R*)-2,3,12,14,15-nonamethyl-4,13-dioxa-3,14-disilahexadecan-5-yl)-2*H*-pyran-2-one (**24**)

Pyrone **22** (50 mg, 78.7 μmol, 1.00 equiv) was dissolved in CH_2_Cl_2_ (1.2 mL) and cooled to 0 °C. *m*-CPBA (≤77%, 17.6 mg, 78.7 μmol, 1.00 equiv) was dissolved in a minimum amount of CH_2_Cl_2_ (∼0.25 mL), and the resulting solution was then added dropwise and slowly, *while carefully monitoring the oxidation progress using TLC analysis* (starting material: *R*_*f*_ = 0.49 in 30% EtOAc in heptane, sulfoxide products: *R*_*f*_ = 0.07 in 30% EtOAc in heptane). When deemed almost complete, the reaction mixture was stirred for 15 min before being quenched by the addition of saturated aqueous NaHCO_3_ (2 mL). The phases were separated, and the aqueous phase was further extracted with CH_2_Cl_2_ (5 × 1.0 mL). The combined organic phase was dried (Na_2_SO_4_), filtrated, and concentrated *in vacuo*. The crude material was purified with flash column chromatography (SiO_2_, 30% → 100% EtOAc in heptane) to yield the sulfoxide products (∼41 mg), which were immediately taken forward in the next reaction.

The obtained material **23** was azeotroped with toluene (3 × 2.0 mL), dissolved in CH_2_Cl_2_ (2.5 mL, dry, stabilized with amylene *and not EtOH*), and cooled to 0 °C. TFAA (35 μL, 0.25 mmol) was added, and the reaction mixture was stirred for 40 min. Then aqueous 1 M NaOH (0.65 mL) and THF (3.7 mL) were added. The reaction mixture was warmed to room temperature and stirred for 2 h. Then the phases were separated, and the aqueous phase was further extracted with EtOAc (4 × 1.0 mL). The combined organic phases were dried (Na_2_SO_4_), filtrated, and concentrated *in vacuo*. The crude material was purified with flash column chromatography (SiO_2_, 30% EtOAc in heptane) to yield **24** (24 mg, 44.2 μmol, 56% over two steps) as a clear oil. *R*_*f*_ (30% EtOAc in heptane, visualized by UV and KMnO_4_ stain) = 0.18; [α]_D_^20^ −86.3 (*c* 1.0, CH_2_Cl_2_); ^1^H NMR (400 MHz, CDCl_3_) δ 6.39 (s, 1H), 4.55 (d, *J* = 6.5 Hz, 2H), 4.50–4.42 (m, 1H), 3.90 (s, 3H), 3.82–3.69 (m, 1H), 2.88 (t, *J* = 6.8 Hz, 1H), 1.81–1.63 (m, 2H), 1.44–1.21 (m, 10H), 1.10 (d, *J* = 6.1 Hz, 3H), 0.94 (s, 9H), 0.88 (s, 9H), 0.10 (s, 3H), 0.04 (2 × s, 6H), 0.03 (s, 3H); ^13^C{^1^H} NMR (101 MHz, CDCl_3_) δ 169.4, 167.0, 165.0, 104.1, 92.7, 71.4, 68.8, 56.6, 54.9, 39.8, 36.6, 29.7, 29.6, 26.1 (3C), 25.9 (3C), 24.5, 24.0, 18.3, 18.3, −4.2, −4.5, −4.7, −4.8; HRESIMS *m*/*z* 565.3350 [M + Na]^+^ (calcd for C_28_H_54_NaO_6_Si_2_, 565.3351).

#### 6-((1*S*,8*R*)-1,8-Dihydroxynonyl)-3-(hydroxymethyl)-4-methoxy-2*H*-pyran-2-one (**1**)

To bis-TBS-protected pyrone **24** (14 mg, 25.8 μmol, 1.00 equiv) was added TBAF (1 M in THF, 129 μL, 129 μmol, 5.00 equiv), and the reaction mixture was stirred until deemed complete by TLC (*R*_*f*_ = 0.47 in EtOAc when one TBS has been removed; *R*_*f*_ = 0.08 in EtOAc when both are gone). Glacial AcOH (6 μL, 105 μmol, 4.10 equiv) was added, and the reaction mixture was stirred for another 15 min. The reaction mixture was then concentrated *in vacuo*. The crude material was loaded onto a short silica gel column using EtOAc and eluted with the same solvent in order to remove as much as possible of the tetrabutylammonium salts (which elutes just after the desired product). The fractions containing product were placed in a −20 °C freezer and allowed to stand until a precipitate formed. The supernatant was quickly removed with a Pasteur pipet, and the solid material was swiftly washed with a small amount of EtOAc (which had been precooled in the same freezer). The material thus obtained was purified again by flash column chromatography (SiO_2_, 50% EtOAc in heptane → EtOAc) through a short column in order to remove the last remaining traces of TBAF salts, yielding dothideopyrone E (**1**, 5.0 mg, 15.9 μmol, 62%) as a clear oil. *R*_*f*_ (EtOAc, visualized by UV and KMnO_4_ stain) = 0.08; [α]_D_^21^ −89.9 (*c* 0.05, MeOH), lit.:^2^ [α]_D_^21^ −92.7 (*c* 0.05, MeOH); ^1^H NMR (400 MHz, DMSO-*d*_6_) δ 6.55 (s, 1H), 5.72 (s, 1H), 4.53 (s, 1H), 4.28 (dd, *J* = 7.8, 4.6 Hz, 2H), 4.19 (s, 2H), 3.91 (s, 3H), 3.61–3.48 (m, 1H), 1.70–1.62 (m, 1H), 1.61–1.53 (m, 1H), 1.40–1.17 (m, 10H), 1.02 (d, *J* = 6.1 Hz, 3H); ^13^C{^1^H} NMR (101 MHz, DMSO-*d*_6_) δ 169.1, 168.2, 164.0, 104.2, 93.5, 69.8, 66.2, 57.3, 52.6, 39.5, 35.3, 29.6, 29.4, 25.8, 25.1, 24.1; HRESIMS *m*/*z* 337.1620 [M + Na]^+^ (calcd for C_16_H_26_NaO_6_, 337.1622).

## Supporting Information Available

^1^H and ^13^C NMR, UV/vis, HPLC, and HRMS data of dothideopyrones E (**1**) and F (**2**) and synthetic intermediates (PDF)

## Dedication

Dedicated to Professor Yngve Henning Stenstrøm on the occasion of his retirement.
