# Supplementary material for: A Modular Strategy for the Synthesis of Dothideopyrones E and F, Secondary Metabolites from an Endolichenic Fungus
Source: J Nat Prod. 2023 Mar 31;86(4):804–11. doi: 10.1021/acs.jnatprod.2c00991 (PMC10152449; doi:10.1021/acs.jnatprod.2c00991)
Supplement: Supplementary file 1 — np2c00991_si_001.pdf [file np2c00991_si_001.pdf]

# A Modular Strategy for the Synthesis of Dothideopyrones E and F, Secondary Metabolites from an Endolichenic Fungus.

*Marius Aursnes[a,b]*, Karoline Gangestad Primdahl[b], David Liwara[c,d] and Eirik Johansson Solum[c,e]**

[a] Department of Chemistry, Biotechnology and Food Science, Norwegian University of Life Sciences, P.O. Box 5003, NO-1433 Ås, Norway

[b] Department of Pharmacy, Section for Pharmaceutical Chemistry, University of Oslo, P.O. Box 1068, 0316 Oslo, Norway

[c] Department of Chemistry Faculty of Natural Sciences, Norwegian University of Science and Technology, NO-7491 Trondheim, Norway

[d] Ecole Centrale de Marseille, 13013 Marseille, France

[e] Faculty of Health Sciences, Nord University, Norway

## Innhold

Synthetic route to dothideopyrone F ........ 2

General Experimental Procedures ........ 3

$^{1}$H and $^{13}$C NMR-Spectra ........ 12

UV-Vis Spectra ........ 39

HPLC-chromatograms ........ 41

HRMS-Spectra ........ 47

# Synthetic route to dothideopyrone F

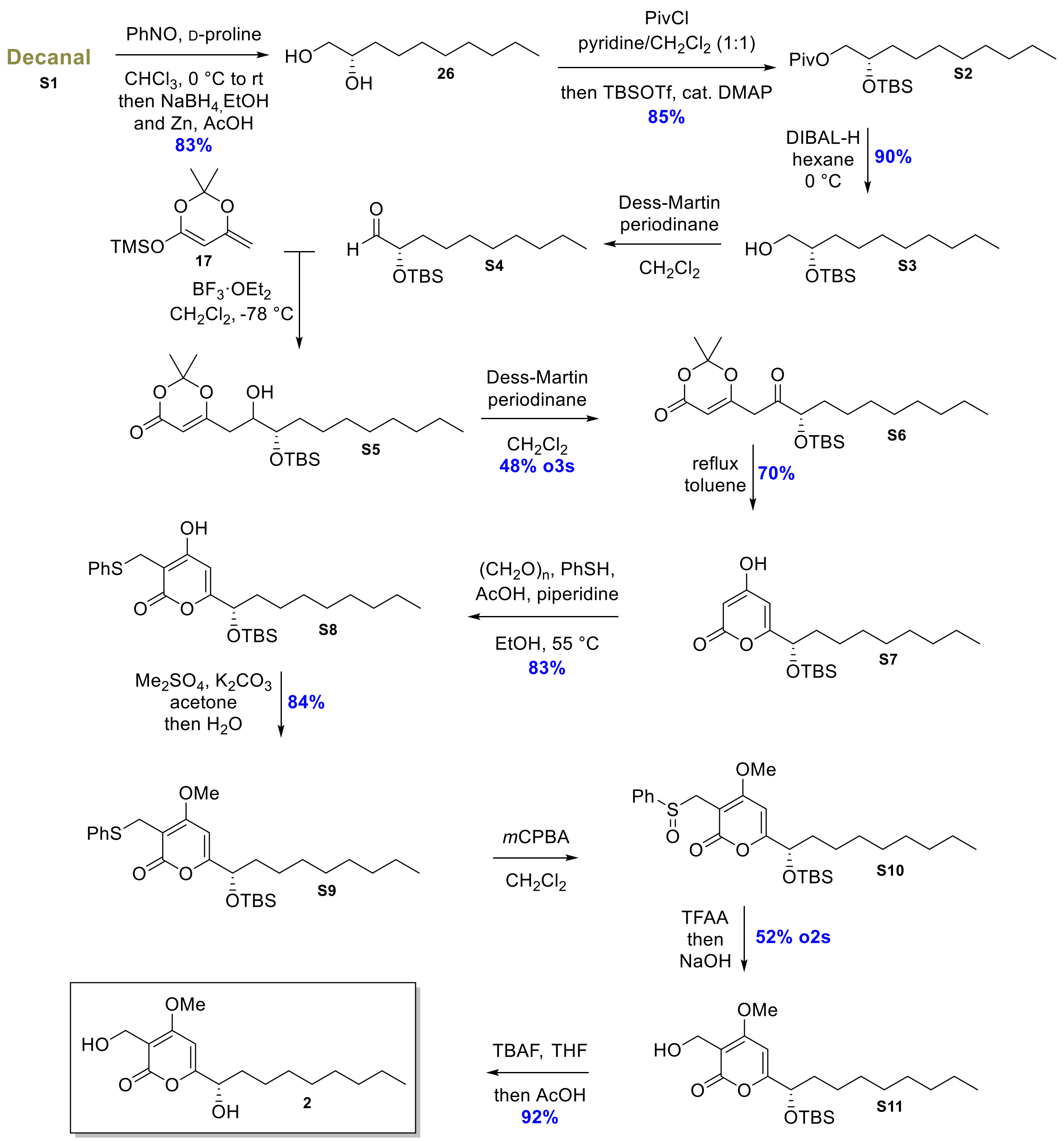

## General Experimental Procedures.

Optical rotations were measured using a 0.7 mL cell with a 1.0 dm path length on an Anton Paar MCP 100 polarimeter. The UV/vis spectra from 190 to 900 nm were recorded using an Agilent Technologies Cary 8485 UV/vis spectrophotometer using quartz cuvettes. NMR spectra were recorded on a Bruker NEO400 or a Bruker AVIII HD 400 spectrometer at 400 MHz or a Bruker AVII600 spectrometer at 600 MHz for $^{1}$H NMR and at 100 or 150 MHz for $^{13}$C NMR. Spectra are referenced relative to the central residual protium solvent resonance in $^{1}$H NMR ($CDCl_3$ δH = 7.26, DMSO-$d_6$ δH = 2.50, and MeOH-$d_4$ δH = 3.31) and the central carbon solvent resonance in $^{13}$C NMR ($CDCl_3$ δC = 77.00, DMSO-$d_6$ δC = 39.52, and MeOH-$d_4$ δC = 49.00). Mass spectra were recorded at 70 eV on a Waters Prospec Q or Micromass QTOF 2W spectrometer using ESI as the method of ionization. High resolution mass spectra were recorded at 70 eV on a Waters Prospec Q or Micromass QTOF 2W spectrometer using ESI as the method of ionization. Thin-layer chromatography was performed on silica gel 60 F254 aluminum-backed plates fabricated by Merck (Darmstadt, Germany). Flash column chromatography was performed on silica gel 60 (40−63 μm) produced by Merck (Darmstadt, Germany). Determination of enantiomeric excess was performed by HPLC on an Agilent Technologies 1200 Series instrument with a diode array detector set at the wavelength stated and equipped with a chiral stationary phase (Chiralpak AD-H, 4.6 × 250 mm, particle size 5 μm or Chiralcel OD-H, 4.6 × 250 mm, particle size 5 μm, both from Daicel Chemical Ind., Ltd), applying the conditions stated. Achiral HPLC analyses were performed using a C18 stationary phase (Eclipse XDB-C18, 4.6 × 250 mm, particle size 5 μm, from Agilent Technologies), applying the conditions stated. Unless stated otherwise, all commercially available reagents and solvents were used in the form they were supplied without any further purification. All reactions were performed under an argon atmosphere, unless otherwise stated. The stated yields are based on isolated material. Liquid chromatography-grade solvents were purchased from Fisher Scientific (Oslo, Norway).

## (*S*)-decane-1,2-diol (26)

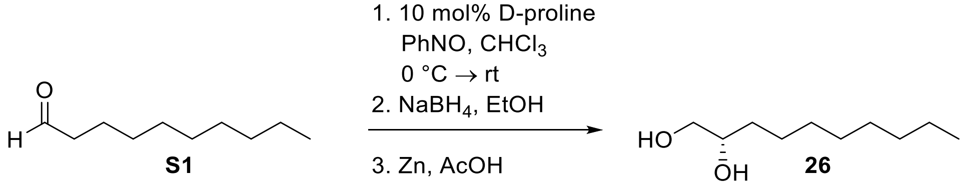

Nitrosobenzene (536 mg, 5.00 mmol, 1.00 equiv.) and D-proline (58.0 mg, 0.50 mmol, 10.0 mol%) were dissolved in $CHCl_3$ (2.5 mL) and cooled to 0 °C. Decanal (**S1**) (2.83 mL, 2.346 g, 15.0 mmol, 3.00 equiv.) was added dropwise, and the reaction was stirred at 0 °C for 2 h. The reaction mixture was then added dropwise to a solution of $NaBH_4$ (567 mg, 15.0 mmol, 3.00 equiv.) in EtOH (30 mL) at 0 °C and stirred at this temperature for an additional 2 h. The solvent was removed *in vacuo* and to the product was added sat. aq. $NaHCO_3$ (10 mL) followed by extraction with EtOAc (3 × 10 mL). The combined organic phase was dried ($Na_2SO_4$) and concentrated *in vacuo*. The product was dissolved in EtOH/AcOH (3:1, 28.0 mL) and zinc powder (3.27 g, 50.0 mmol, 10.0 equiv) was added. The reaction mixture was stirred at room temperature overnight, filtrated through Celite, and concentrated *in vacuo*. The material thus obtained was purified by flash column chromatography ($SiO_2$, 50 → 70% EtOAc in hexane) to give the desired diol **26** (724 mg, 4.15 mmol, 83%) as a clear oil. The spectroscopic data was in agreement with previously reported data.[1] $R_f$ (60% EtOAc in heptane, visualized by $KMnO_4$-stain) = 0,18; $[\alpha]_D^{23}$ -11.2 (*c* 0.44, MeOH), Litt:[1] $[\alpha]_D^{23}$ -11.9 (*c* 0.43, MeOH); $^1$H NMR (400 MHz, $CDCl_3$) δ 3.79 – 3.57 (m, 2H), 3.44 (ddd, *J* = 11.3, 7.4, 3.9 Hz, 1H), 2.01 (d, *J* = 4.0 Hz, 1H), 1.94 – 1.83 (m, 1H), 1.48 – 1.20 (m, 14H), 0.88 (t, *J* = 6.8 Hz, 3H); $^{13}C\{^1H\}$ NMR (101 MHz, $CDCl_3$) δ 72.5, 67.0, 33.4, 32.0, 29.8, 29.7, 29.4, 25.7, 22.8, 14.3.

A small amount of the *α*-aminoxylated alcohol intermediate was kept for HPLC analysis. The enantiomeric excess (>94%), was determined by HPLC analysis using a chiral column (AD-H, *i*PrOH/hexane, 5:95, 1.0 mL/min): $t_r$(major) = 12.769 min, $t_r$(minor) = 15.631 min. *The racemate was made using DL-proline following same procedure and approach as described above.*

**(*S*)-2-((*tert*-butyldimethylsilyl)oxy)decyl pivalate (S2)**

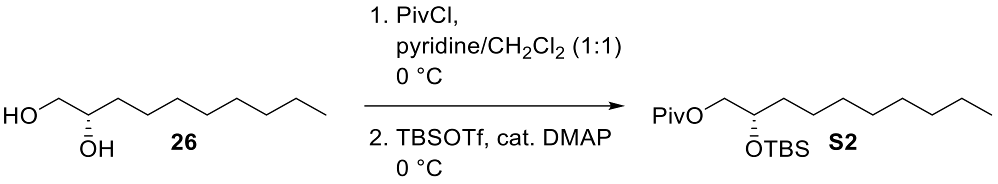


Diol **26** (720 mg, 4.13 mmol, 1.00 equiv.) was dissolved in a 1:1 mixture of $CH_2Cl_2$/pyridine (12.3 mL) and cooled to 0 °C. Then, trimethylacetyl chloride (0.61 mL, 0.60 g, 4.96 mmol, 1.20 equiv.) was added dropwise. The reaction mixture was stirred at 0 °C until deemed complete by TLC. TBSOTf (2.37 mL, 2.73 g, 10.3 mmol, 2.50 equiv.) was then added dropwise followed by addition of one crystal of DMAP. Stirring was continued at 0 °C until deemed complete by TLC. The reaction mixture was quenched with sat. aq. $NaHCO_3$ (30 mL), extracted with EtOAc (3 × 15 mL), dried ($Na_2SO_4$), filtrated, and concentrated *in vacuo*. The crude product thus obtained was purified by flash chromatography ($SiO_2$, heptane → 2% EtOAc in heptane) to yield **S2** (1302 mg, 3.49 mmol, 85%) as a clear oil. $R_f$ (1% EtOAc in heptane, visualized by CAM-stain and prolonged heating) = 0.20; $[\alpha]_D^{25}$ +13.1 (*c* 0.57, $CHCl_3$); $^1H$ NMR (400 MHz, $CDCl_3$) δ 3.96 (dd, *J* = 5.3, 1.0 Hz, 2H), 3.83 (p, *J* = 5.5 Hz, 1H), 1.56 – 1.41 (m, 2H), 1.33 – 1.25 (m, 12H), 1.20 (s, 9H), 0.90 – 0.87 (m, 12H), 0.07 (s, 3H), 0.06 (s, 3H); $^{13}C\{^1H\}$ NMR (101 MHz, $CDCl_3$) δ $^{13}C$ NMR (101 MHz, $CDCl_3$) δ 178.7, 70.2, 68.3, 38.9, 34.8, 32.0, 29.9, 29.7, 29.4, 27.4 (3C), 25.9 (3C), 25.1, 22.8, 18.2, 14.3, -4.4, -4.5; HRESIMS *m*/*z* 395.2950 $[M + Na]^+$ (calcd for $C_{21}H_{44}O_3Si$, 395.2952).

**(*S*)-2-((*tert*-butyldimethylsilyl)oxy)decan-1-ol (S3)**

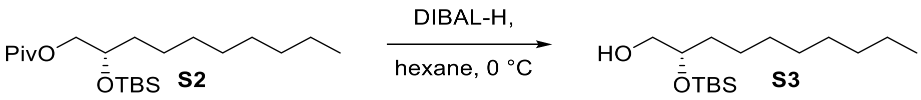


The pivalate **S2** (695 mg, 1.86 mmol, 1.00 equiv) was dissolved in hexane (4.4 mL) and cooled to 0 °C. DIBAL-H (1.0 M in hexane, 4.66 mL, 4.66 mmol, 2.50 equiv) was added dropwise, and the reaction mixture was stirred until deemed complete by TLC. MeOH (2.5 mL) was added to quench the reaction followed by addition of sat. aq. potassium sodium tartrate (18 mL). After vigorous stirring and phase separation, the aqueous phase was extracted with $Et_2O$ (4 × 5 mL). The combined organic phase was dried ($Na_2SO_4$), filtrated, concentrated in vacuo, and then kept under

high vacuum for 4 h. The resulting alcohol was purified by column chromatography ($SiO_2$, heptane → 25% EtOAc in heptane) to give the alcohol **S3** (484 mg, 1.68 mmol, 90%) as a colorless oil. $R_f$ (20% EtOAc in heptane, visualized by $KMnO_4$-stain) = 0.36; $[\alpha]_D^{25}$ +14.7 (*c* 1.5, $CHCl_3$); $^1$H NMR (400 MHz, $CDCl_3$) δ 3.76 – 3.69 (m, 1H), 3.56 (dd, *J* = 11.0, 3.6 Hz, 1H), 3.44 (dd, *J* = 11.0, 5.4 Hz, 1H), 1.87 (bs, 1H), 1.53 – 1.43 (aq, *J* = 6.8 Hz, 2H), 1.35 – 1.21 (m, 12H), 0.91 (s, 9H), 0.88 (t, *J* = 6.9 Hz, 3H), 0.09 (s, 6H); $^{13}$C{$^1$H} NMR (101 MHz, $CDCl_3$) δ 73.2, 66.5, 34.2, 32.0, 29.9, 29.7, 29.4, 26.0 (3C), 25.5, 22.8, 18.3, 14.2, -4.3, -4.4; HRESIMS *m/z* 311.2375 $[M + Na]^+$ (calcd for $C_{16}H_{36}NaO_2Si$, 311.2377).

**(*S*)-6-(3-((*tert*-butyldimethylsilyl)oxy)-2-oxoundecyl)-2-dimethyl-4*H*-1,3-dioxin-4-one (S6)**

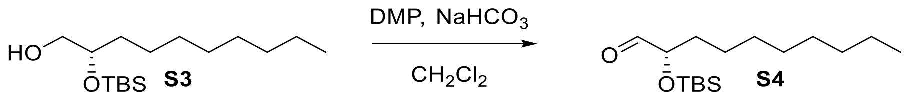


Alcohol **S3** (450 mg, 1.56 mmol, 1.00 equiv) was dissolved in $CH_2Cl_2$ (45 mL) and cooled to 0°C. Dess−Martin periodinane reagent (794 mg, 1.87 mmol, 1.2 equiv.) and $NaHCO_3$ (50 mg) were added in one portion. The reaction mixture was removed from the cooling bath and stirring was continued for ~4 h. The reaction was quenched by addition of a saturated solution of $Na_2S_2O_3$ (10 mL). The aqueous phase was extracted with $CH_2Cl_2$ (4 × 15 mL). The combined organic phase was dried ($Na_2SO_4$), filtrated and concentrated *in vacuo*. The crude product thus obtained was filtrated through a short plug of silica gel (0 → 10% EtOAc in heptane to remove the leftover and spent DMP-reagent and then concentrated *in vacuo*.

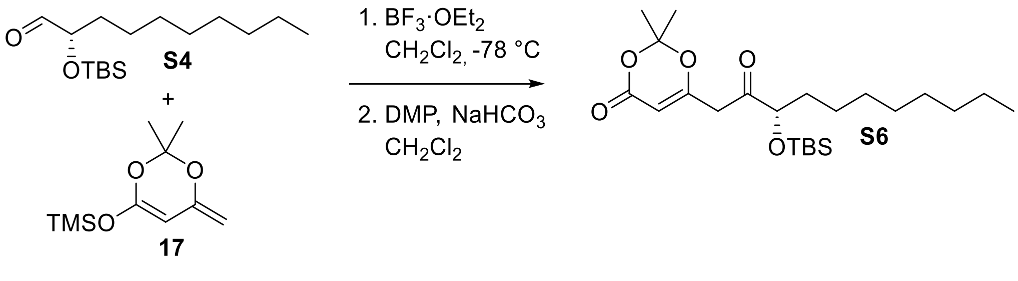


Aldehyde **S4** (385 mg, 1.34 mmol, 1.00 equiv.) was azeotropically dried with 2-MeTHF (2 × 5 mL) and then dissolved in $CH_2Cl_2$ (13 mL). Ketene acetal **17** (720 mg, 3.36 mmol, 2.50 equiv.) was added and the reaction mixture was cooled to -78 °C. Next, $BF_3·OEt_2$ (497 µL, 4.03 mmol, 3

equiv.) was added dropwise over 30 min. The reaction mixture was stirred for 1 hour and then quenched by the addition of phosphate buffer (12 mL, pH = 7), warmed to room temperature and then sat. aq. $NaHCO_3$ (25 mL) was added. The phases were separated and the aqueous phase was extracted with $CH_2Cl_2$ (3 × 10 mL). The combined organic phase was dried ($Na_2SO_4$), filtrated and concentrated in vacuo. The aldol product co-eluted with the hydrolized ketene acetal (i.e. 2,2,6-trimethyl-4*H*-1,3-dioxin-4-one) and the crude material was therefore directly taken up in $CH_2Cl_2$ (40 mL) and cooled to 0 °C. Next, the Dess−Martin periodinane reagent (680 mg, 1.60 mmol, 1.20 equiv.) and $NaHCO_3$ (100 mg, 1.19 mmol, 0.90 equiv.) were added and the flask was removed from the cooling bath. The reaction mixture was stirred until deemed complete by TLC analysis. The reaction was quenched by addition of a saturated solution of $Na_2S_2O_3$ (10 mL). The aqueous phase was extracted with $CH_2Cl_2$ (4 × 15 mL). The combined organic phase was dried ($Na_2SO_4$), filtrated and concentrated *in vacuo*. The crude product thus obtained was filtrated through a short plug of silica gel ($SiO_2$, 20% EtOAc in heptane) to yield ketone **S6** (319 mg, 0.75 mmol, 48% o3s) as a clear oil. $R_f$ (20% EtOAc in heptane, visualized by $KMnO_4$-stain) = 0.34; $[\alpha]_D^{25}$ -22.2 (*c* 1.0, $CHCl_3$); $^1H$ NMR (400 MHz, $CDCl_3$) δ 5.32 (s, 1H), 4.05 (t, *J* = 6.1 Hz, 1H), 3.48 (s, 2H), 1.71 (s, 3H), 1.70 (s, 3H), 1.64 (q, *J* = 7.3 Hz, 1H), 1.63 – 1.49 (m, 1H), 1.34 – 1.23 (m, 12H), 0.93 (s, 9H), 0.88 (t, *J* = 6.8 Hz, 3H), 0.09 (s, 3H), 0.07 (s, 3H); $^{13}C\{^1H\}$ NMR (101 MHz, $CDCl_3$) δ 207.1, 165.5, 160.9, 170.3, 97.1, 78.9, 41.8, 34.9, 32.0, 29.6, 29.5, 29.3, 25.9 (3C), 25.2, 25.2, 24.6, 22.8, 18.2, 14.2, -4.7, -4.8; HRESIMS *m*/*z* 449.2693 $[M + Na]^+$ (calcd for $C_{23}H_{42}NaO_5Si$, 449.2694).

**(*S*)-6-(1-((*tert*-butyldimethylsilyl)oxy)nonyl)-4-hydroxy-2*H*-pyran-2-one (S7)**

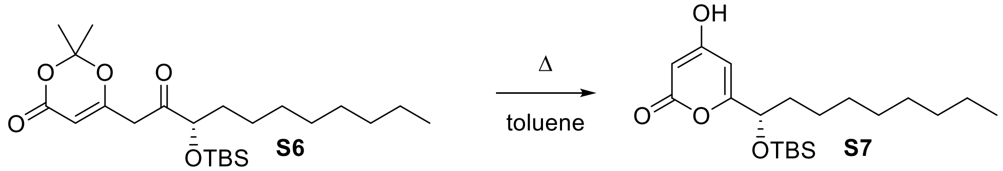


Ketone **S6** (302 mg, 0.71 mmol, 1.00 equiv.) was azeotroped with toluene (2 × 3 mL) and then dissolved in toluene (4.4 mL). This solution was added dropwise to a solution of boiling toluene (28 mL) over a period of 10 min. More toluene (2 × ~0.5 mL) was used to wash the flask and the washings were added to the refluxing reaction mixture. The solution was further refluxed for 45 min, cooled to room-temperature and concentrated *in vacuo*. The crude product thus obtained was

purified by flash column chromatography ($SiO_2$, 50% EtOAc in heptane, tailing. $KMnO_4$-stain.) to yield pyrone **S7** (182 mg, 0.49 mmol, 70%) as a yellow oil. $R_f$ (50% EtOAc in heptane, visualized by $KMnO_4$-stain) = 0.19; $[\alpha]_D^{25}$ -101.3 (*c* 1.6, $CHCl_3$); $^1$H NMR (400 MHz, $CDCl_3$) δ 10.75 (bs, 1H), 6.26 (dd, *J* = 2.2, 0.9 Hz, 1H), 5.58 (d, *J* = 2.2 Hz, 1H), 4.43 (t, *J* = 5.3 Hz, 1H), 1.80 – 1.61 (m, 2H), 1.50 – 1.12 (m, 12H), 0.92 (s, 9H), 0.87 (t, *J* = 6.9 Hz, 3H), 0.08 (s, 3H), 0.03 (s, 3H). 0.75 (bs, 1H), 6.26 (dd, *J* = 2.2, 0.9 Hz, 1H), 5.58 (d, *J* = 2.2 Hz, 1H), 4.43 (t, *J* = 5.3 Hz, 1H), 1.80 – 1.61 (m, 2H), 1.50 – 1.12 (m, 12H), 0.92 (s, 9H), 0.87 (t, *J* = 6.9 Hz, 3H), 0.08 (s, 3H), 0.03 (s, 3H); $^{13}C\{^1H\}$ NMR (101 MHz, $CDCl_3$) δ 172.6, 169.5, 167.6, 99.9, 90.3, 71.2, 36.4, 32.0, 29.6 (2C), 29.4, 25.9 (3C), 24.5, 22.8, 18.2, 14.3, -4.7, -4.9; HRESIMS *m/z* 391.2276 $[M + Na]^+$ (calcd for $C_{20}H_{36}NaO_4Si$, 391.2275).

**(*S*)-6-(1-((*tert*-butyldimethylsilyl)oxy)nonyl)-4-hydroxy-3-((phenylthio)methyl)-2*H*-pyran-2-one (S8)**

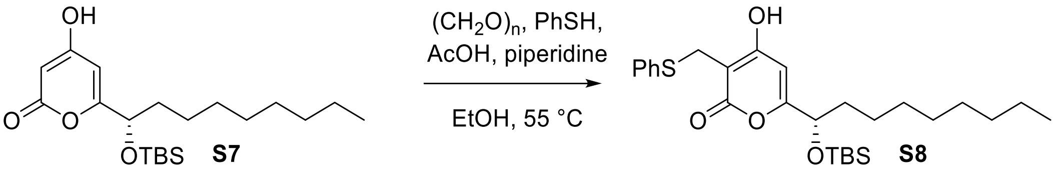


Pyrone **S7** (45.0 mg, 122 µmol, 1.00 equiv.) was dissolved in EtOH (6.0 mL) and then added to a suspension consisting of paraformaldehyde (6.0, 0.20 mmol as monomer, 1.6 equiv.), thiophenol (100 µL, 0.97 mmol, 8.0 equiv.), acetic acid (6.1 µL, 107 µmol, 0.87 equiv.) and piperidine (6.1 µL, 62 µmol, 0.51 equiv.) in EtOH (4.0 mL) at 55 °C (oil-bath temperature). The reaction was stirred for 22 hours at said temperature, cooled to room temperature and concentrated *in vacuo*. The crude product thus obtained was purified by flash column chromatography ($SiO_2$, 0 → 30% EtOAc in heptane, visualized by UV and $KMnO_4$-stain) to yield pyrone **S8** (50 mg, 0.10 mmol, 83%) as a yellow oil. $R_f$ (30% EtOAc in heptane, visualized by UV and $KMnO_4$-stain) = 0.30; $[\alpha]_D^{25}$ -49.2 (*c* 1.7, $CH_2Cl_2$); $^1$H NMR (400 MHz, $CDCl_3$) δ 7.41 – 7.37 (m, 2H), 7.29 – 7.19 (m, 3H), 6.13 (s, 1H), 4.36 (t, *J* = 5.3 Hz, 1H), 4.10 (s, 2H), 1.77 – 1.61 (m, 2H), 1.42 – 1.15 (m, 12H), 0.89 (s, 9H), 0.87 (t, *J* = 6.8 Hz, 3H), 0.05 (s, 3H), -0.01 (s, 3H); $^{13}C\{^1H\}$ NMR (101 MHz, $CDCl_3$) δ 167.8, 167.5, 164.9, 133.6, 130.6 (2C), 129.2 (2C), 127.4, 98.9, 97.7, 71.1, 36.3, 32.0, 29.6, 29.6,

29.4, 28.5, 25.9 (3C), 24.4, 22.8, 18.2, 14.3, -4.7, -4.9; HRESIMS *m*/*z* 513.2464 [M + Na]$^+$ (calcd for $C_{27}H_{42}NaO_4SSi$, 513.2465).

**(*S*)-6-(1-((*tert*-butyldimethylsilyl)oxy)nonyl)-4-methoxy-3-((phenylthio)methyl)-2*H*-pyran-2-one (S9)**

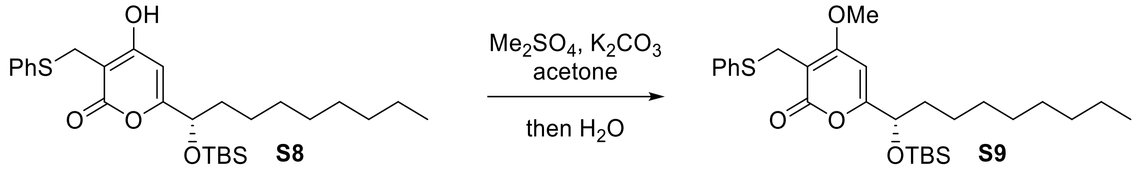


Pyrone **S8** (50 mg, 102 µmol, 1.00 equiv.) was dissolved in acetone (1.00 mL). Dimethyl sulfate (50 µL, 528 µmol, 5.2 equiv.) was added followed by $K_2CO_3$ (74 mg, 535 µmol, 5.3 equiv.). The reaction mixture was stirred for one hour, water (0.55 mL) was added and then the reaction mixture was vigorously stirred overnight. Sat. aq. $NH_4Cl$ (2 mL) was added and the reaction mixture was extracted with $CH_2Cl_2$ (5 × 1 mL). The combined organic phase was dried ($Na_2SO_4$), filtrated and concentrated *in vacuo*. The crude material thus obtained was purified by flash column chromatography ($SiO_2$, heptane → 30% EtOAc in heptane. UV and $KMnO_4$-stain) to yield pyrone **S9** (43.0 mg, 85.2 µmol, 84%) as a clear oil. $R_f$ (30% EtOAc in heptane, visualized by $KMnO_4$-stain) = 0.45; $[\alpha]_D^{25}$ -61.3 (*c* 2.9, $CH_2Cl_2$); $^1H$ NMR (400 MHz, $CDCl_3$) δ 7.47 – 7.43 (m, 2H), 7.29 – 7.16 (m, 3H), 6.29 (s, 1H), 4.49 – 4.41 (m, 1H), 3.98 (s, 2H), 3.70 (s, 3H), 1.82 – 1.64 (m, 2H), 1.39 – 1.18 (m, 12H), 0.94 (s, 9H), 0.88 (t, *J* = 6.8 Hz, 3H), 0.10 (s, 3H), 0.04 (s, 3H); $^{13}C\{^1H\}$ NMR (101 MHz, $CDCl_3$) δ 168.8, 167.3, 163.8, 136.5, 131.6 (2C), 128.7 (2C), 126.7, 101.7, 92.4, 71.4, 56.4, 36.6, 32.0, 29.6, 29.6, 29.4, 28.6, 25.9 (3C), 24.6, 22.8, 18.3, 14.3, -4.7, -4.8; HRESIMS *m*/*z* 527.2621 [M + Na]$^+$ (calcd for $C_{28}H_{44}NaO_4SSi$, 527.2622).

**(*S*)-6-(1-((*tert*-butyldimethylsilyl)oxy)nonyl)-3-(hydroxymethyl)-4-methoxy-2*H*-pyran-2-one (S11)**

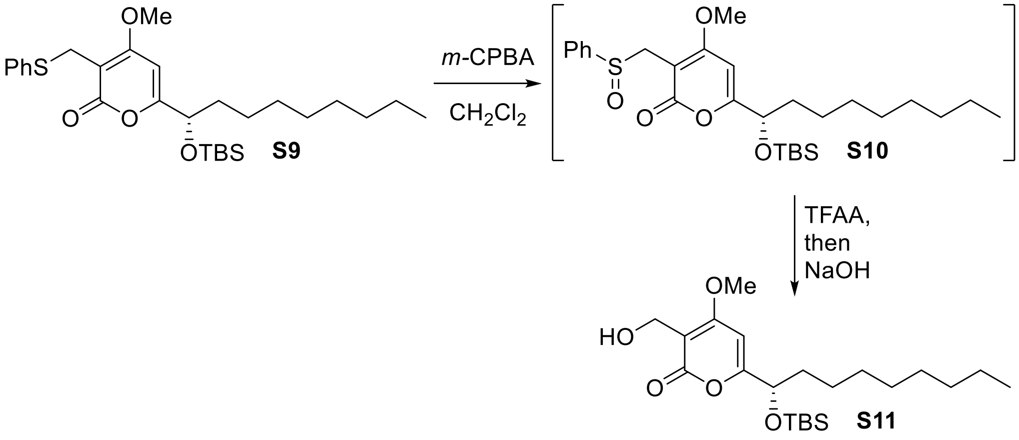


Pyrone **S9** (42 mg, 83.2 µmol, 1.00 equiv.) was dissolved in $CH_2Cl_2$ (1.0 mL) and cooled to 0 °C. *m*-CPBA (≤77%, 18.6 mg, 83.2 µmol, 1.00 equiv.) was dissolved in a minimum amount of $CH_2Cl_2$ (~0.25 mL) and the resulting solution was then added dropwise and slowly – *while carefully monitoring the oxidation progress using TLC analysis* (Starting-material: $R_f$ = 0.49 in 30% EtOAc in heptane, sulfoxide products: $R_f$ = 0.06 in 30% EtOAc in heptane). When deemed almost complete, the reaction mixture was stirred for 15 min before being quenched by the addition of sat. aq. $NaHCO_3$ (2 mL). The phases were separated and the aqueous phase was further extracted with $CH_2Cl_2$ (5 × 1 mL). The combined organic phase was dried ($Na_2SO_4$), filtrated and concentrated *in vacuo*. The crude material was purified with flash column chromatography (30 → 100% EtOAc in heptane) to yield the sulfoxide products (39 mg), which was immediately taken forward in the next reaction.

The obtained material **S10** was azeotroped with toluene (3 × 2 mL), dissolved in $CH_2Cl_2$ (3 mL, dry, stabilized with amylene *and not EtOH*) and cooled to 0 °C. TFAA (42 µL, 63.5 mg, 0.30 mmol) was added and the reaction mixture was stirred for 40 min. Then aqueous 1 M NaOH (0.75 mL) and THF (4.5 mL) were added. The reaction mixture was warmed to room temperature and stirred for 2 hours. Then the phases were separated and the aqueous phase was further extracted with EtOAc (4 × 1 mL). The combined organic phases were dried ($Na_2SO_4$), filtrated and concentrated *in vacuo*. The crude material was purified with flash column chromatography ($SiO_2$, 30% EtOAc in heptane) to yield **S11** (18 mg, 43.6 µmol, 52% o2s). $R_f$ (30% EtOAc in heptane, visualized by $KMnO_4$-stain) = 0.18; $[\alpha]_D^{20}$ -90.9 (*c* 0.2, MeOH); $^1H$ NMR (400 MHz, $CDCl_3$) δ

6.40 (s, 1H), 4.55 (d, *J* = 6.5 Hz, 2H), 4.46 (dd, *J* = 6.5, 4.2 Hz, 1H), 3.90 (s, 3H), 2.87 (t, *J* = 6.8 Hz, 1H), 1.82 – 1.62 (m, 2H), 1.33 – 1.22 (m, 12H), 0.94 (s, 9H), 0.87 (t, *J* = 6.7 Hz, 3H), 0.11 (s, 3H), 0.04 (s, 3H); $^{13}C\{^1H\}$ NMR (101 MHz, $CDCl_3$) δ 169.5, 167.0, 165.0, 104.1, 92.7, 71.4, 56.6, 54.9, 36.6, 32.0, 29.6 (2C), 29.4, 25.9 (3C), 24.5, 22.8, 18.3, 14.3, -4.7, -4.8; HRESIMS *m/z* 435.2537 $[M + Na]^+$ (calcd for $C_{22}H_{40}NaO_5Si$, 435.2537).

**(*S*)-3-(hydroxymethyl)-6-(1-hydroxynonyl)-4-methoxy-2H-pyran-2-one (2)**

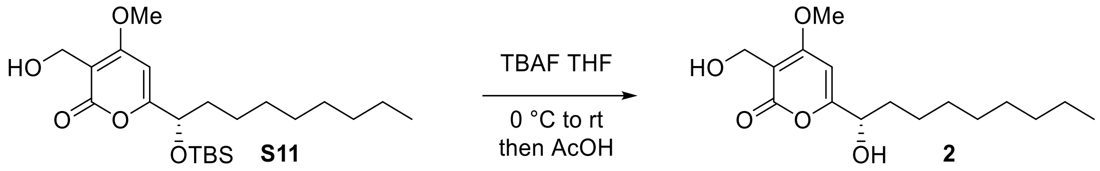


TBS-protected **S11** (18 mg, 43.6 µmol) was dissolved in THF (0.6 mL) and the cooled to 0 °C. TBAF (1 M in THF, 109 µL, 109 µmol, 2.5 equiv.) and the reaction mixture was stirred until deemed complete by TLC. The reaction was quenched by the addition of phosphate buffer (1 mL, pH = 7) and brine (0.2 mL). The reaction mixture was extracted with EtOAc (5 × 0.5 mL), dried ($Na_2SO_4$), filtrated and concentrated *in vacuo*. The material thus obtained was purified by flash column chromatography (50% EtOAc in heptane → 100% EtOAc) through a short column, yielding dothideopyrone F (**2**) as a clear oil (12 mg, 92%). $R_f$ (EtOAc, visualized by UV and $KMnO_4$-stain) = 0.38; $[\alpha]_D^{21}$ -109.2 (*c* 0.05, MeOH); Litt:[2] $[\alpha]_D^{21}$ -118.7 (*c* 0.05, MeOH); $^1H$ NMR (400 MHz, MeOH-$d_4$) δ 6.64 (s, 1H), 4.44 (s, 2H), 4.39 (dd, *J* = 7.9, 4.7 Hz, 1H), 4.00 (s, 3H), 1.90 – 1.77 (m, 1H), 1.73 – 1.62 (m, 1H), 1.52 – 1.24 (m, 12H), 0.90 (t, *J* = 6.5 Hz, 3H); $^{13}C\{^1H\}$ NMR (101 MHz, MeOH-$d_4$) δ 169.3, 165.4, 103.4, 93.1, 70.1, 56.2, 52.6, 34.9, 31.6, 29.2, 29.1, 29.0, 24.8, 22.3, 13.0; HRESIMS *m/z* 321.1673 $[M + Na]^+$ (calcd for $C_{16}H_{26}NaO_5$, 321.1672).

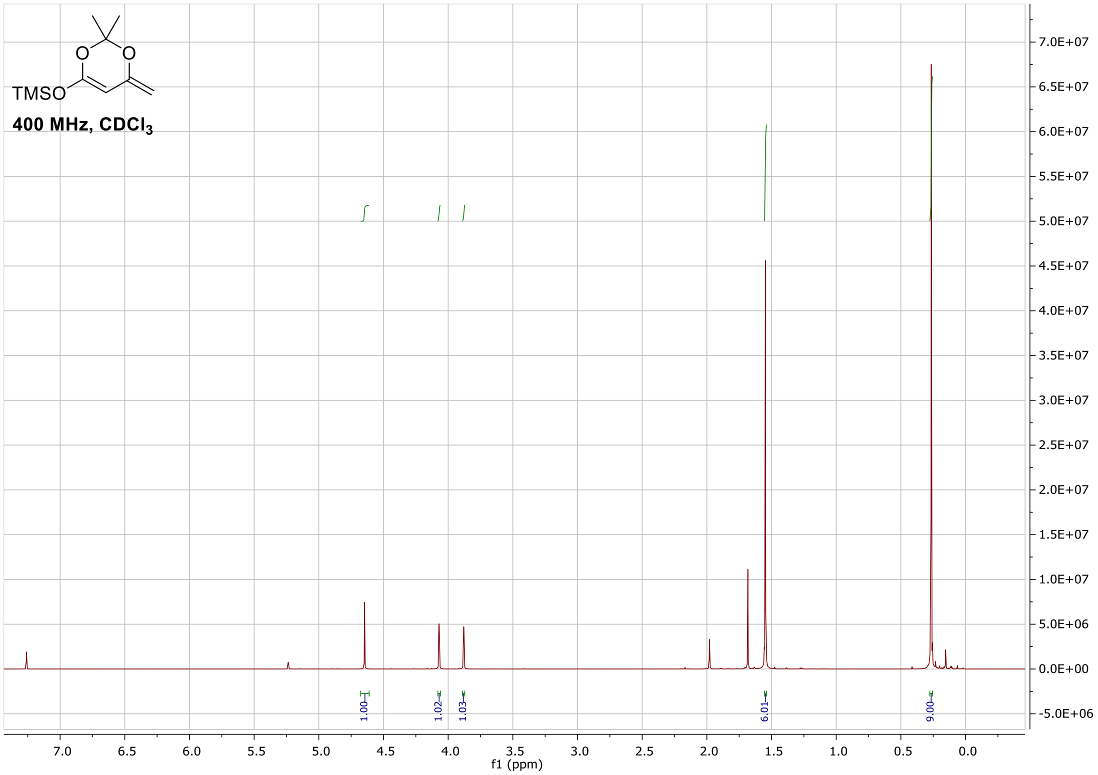


**Figure S-1** $^{1}$H-NMR spectrum of compound **17**.

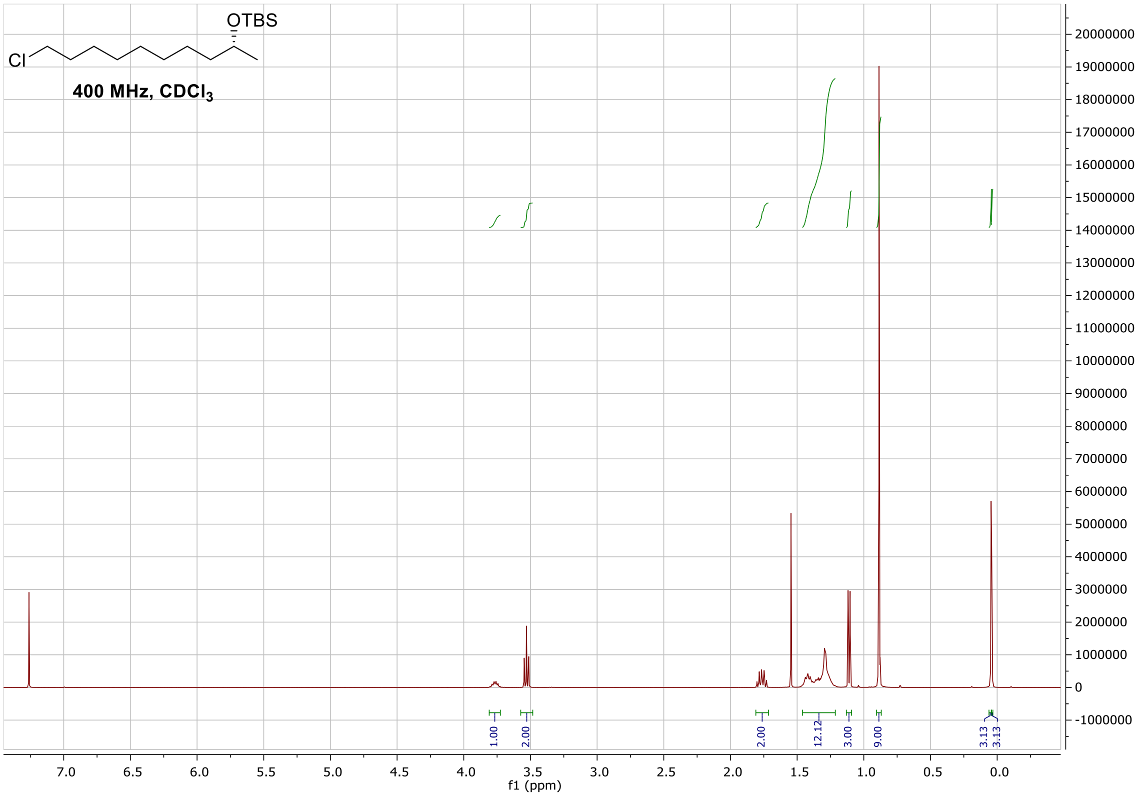


**Figure S-2** $^{1}$H-NMR spectrum of compound **11**.

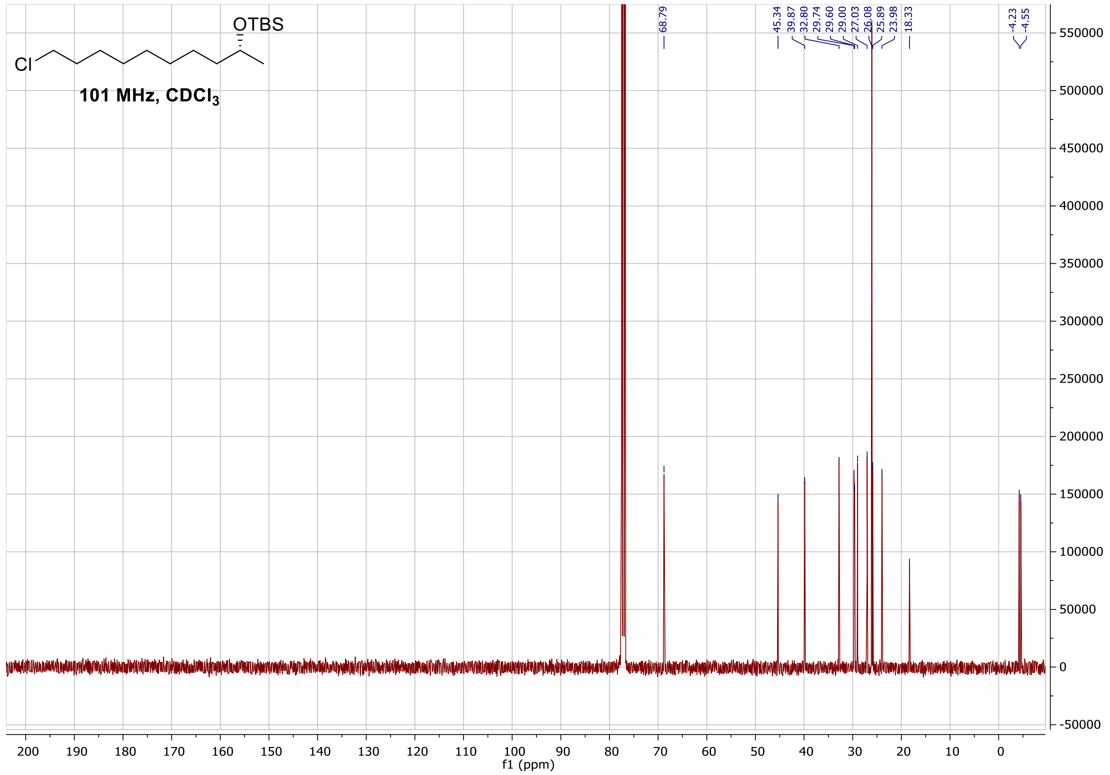


**Figure S-3** $^{13}$C-NMR spectrum of compound **11**.

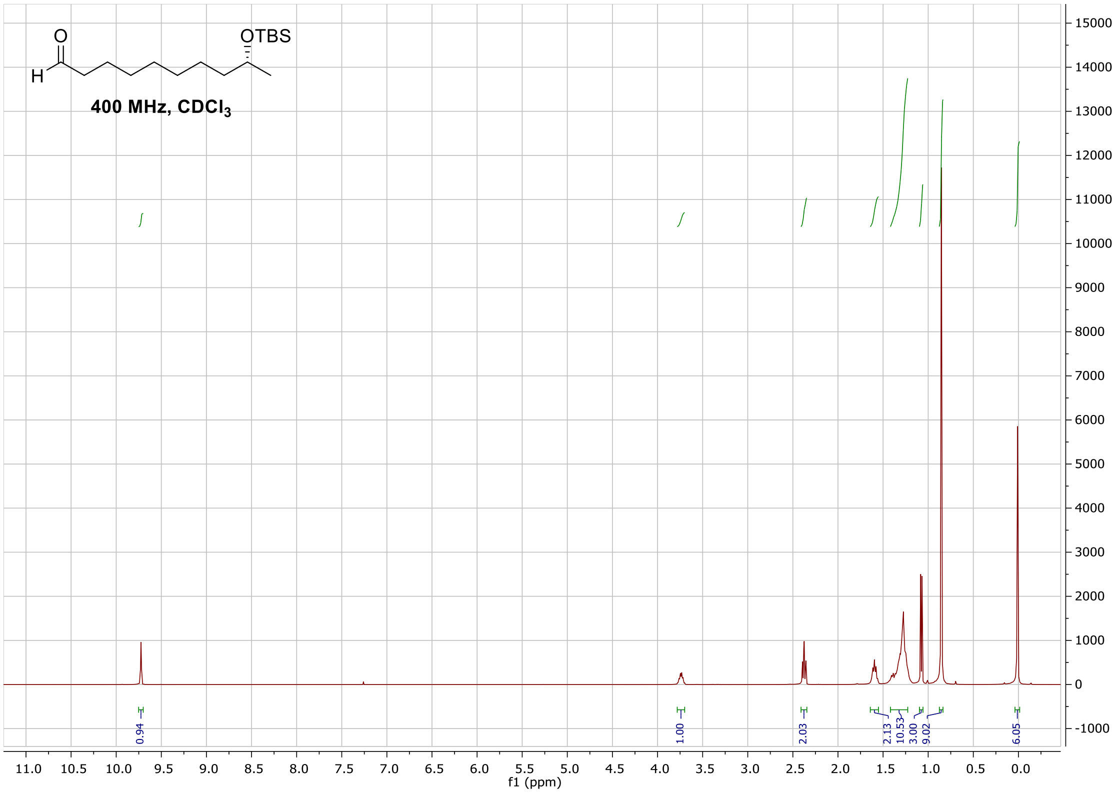


**Figure S-4** $^{1}$H-NMR spectrum of compound **12**.

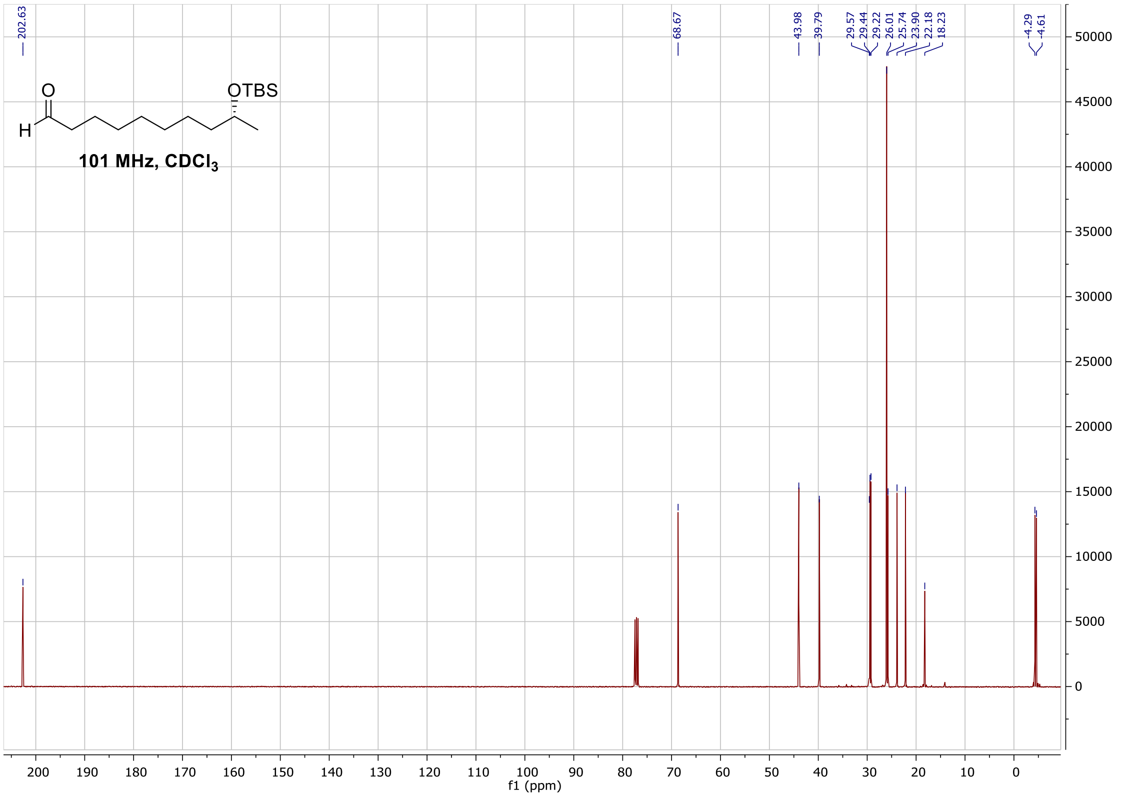


**Figure S-5** $^{13}$C-NMR spectrum of compound **12**.

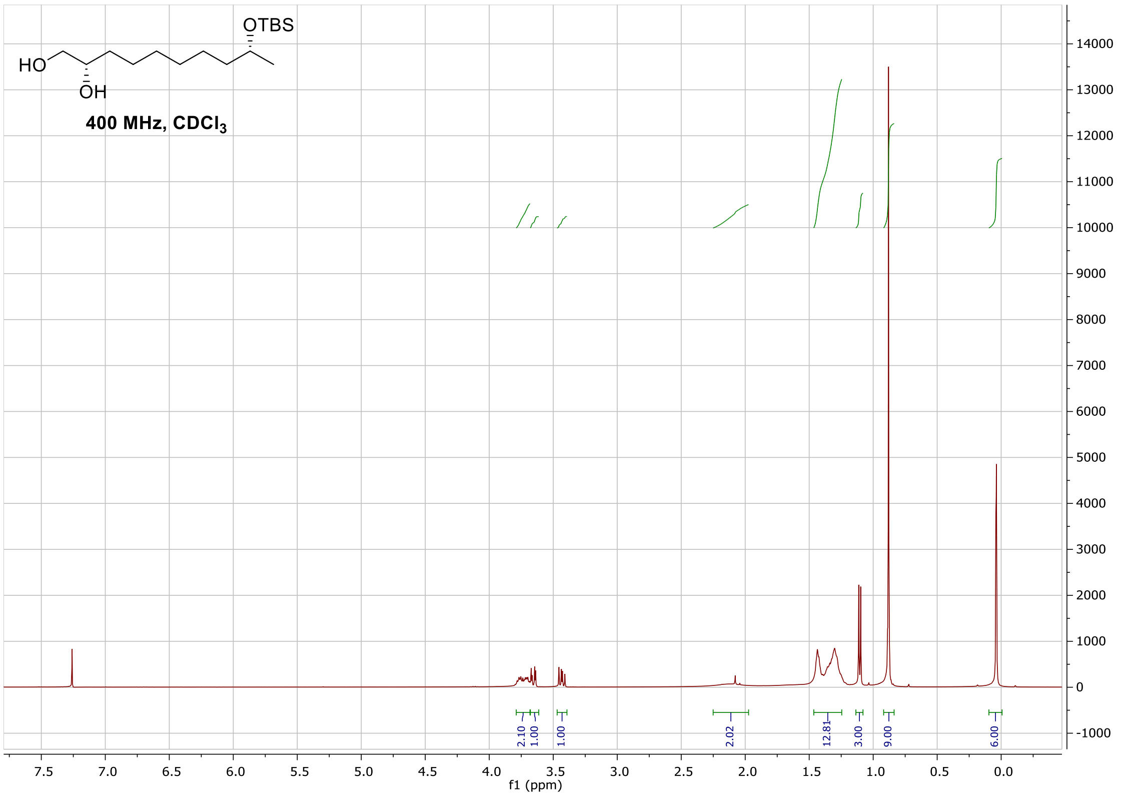


**Figure S-6** $^{1}$H-NMR spectrum of compound **13**.

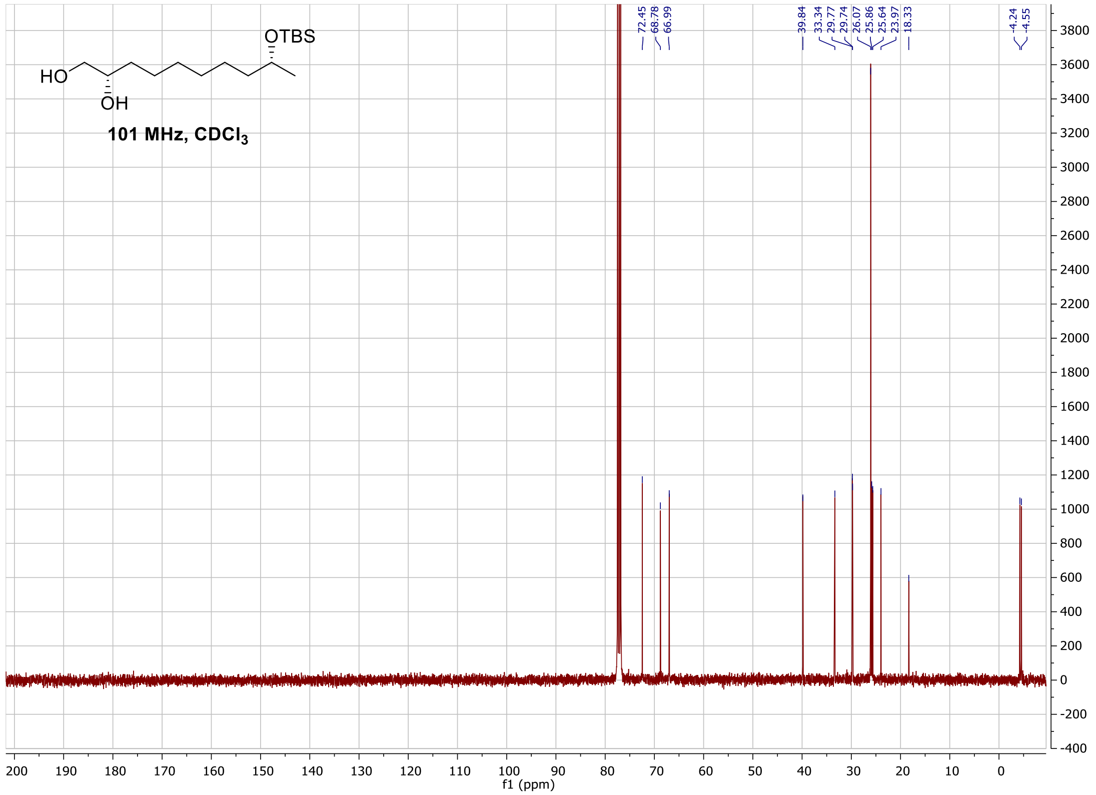


**Figure S-7** $^{13}$C-NMR spectrum of compound **13**.

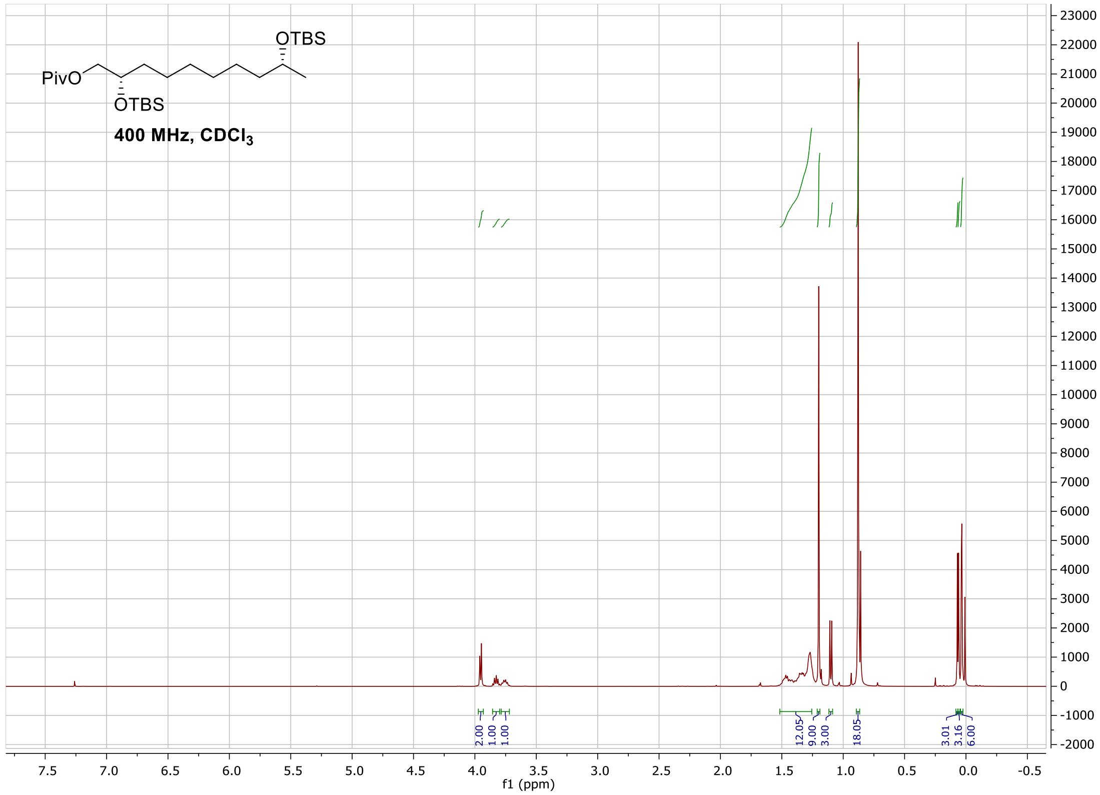


**Figure S-8** $^{1}$H-NMR spectrum of compound **14**.

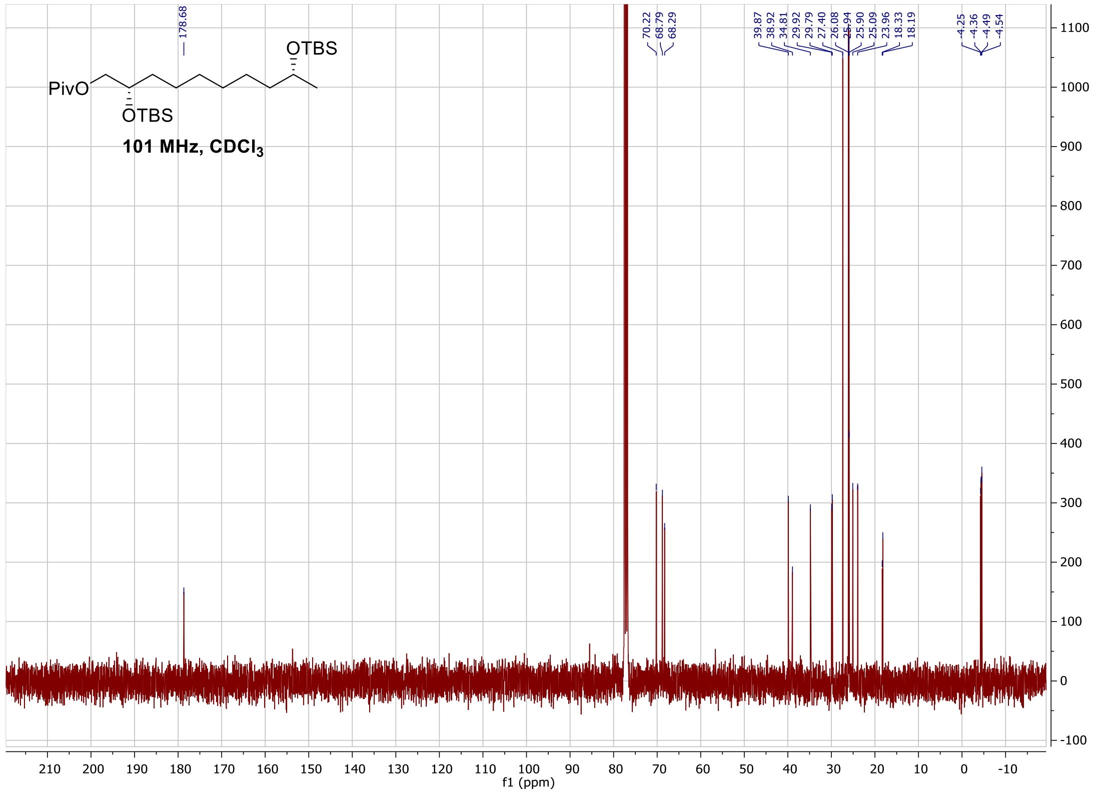


**Figure S-9** $^{13}$C-NMR spectrum of compound **14**.

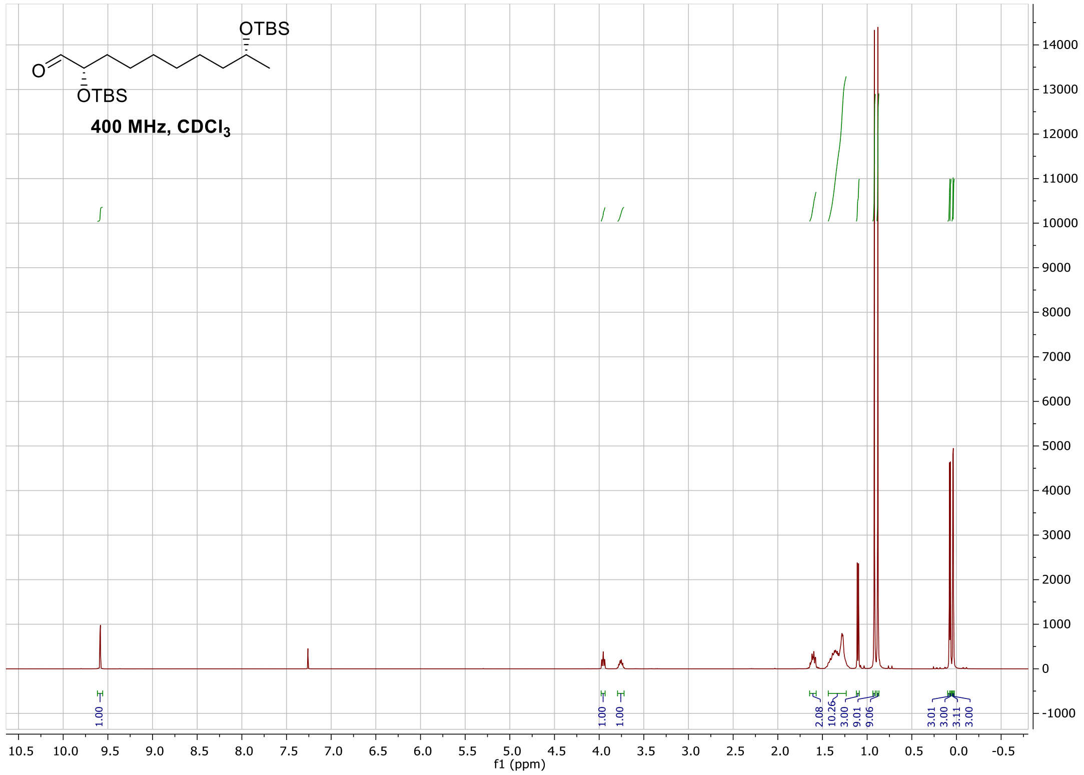


**Figure S-10** $^{1}$H-NMR spectrum of compound **16**.

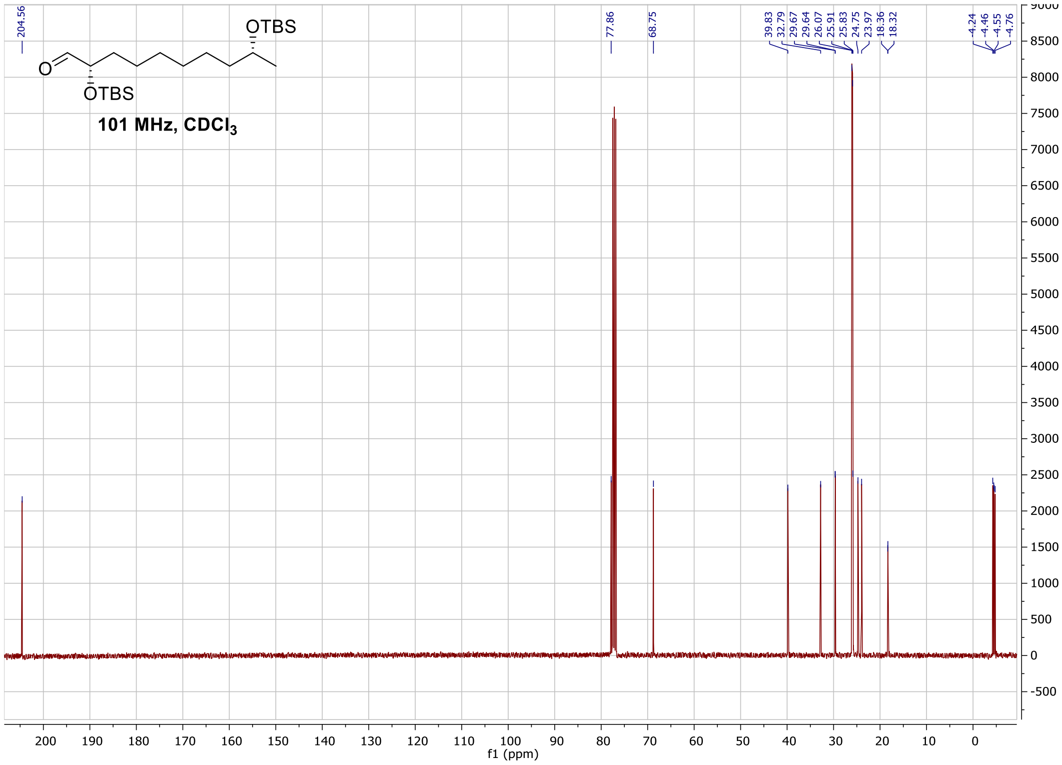


**Figure S-11** $^{13}$C-NMR spectrum of compound **16**.

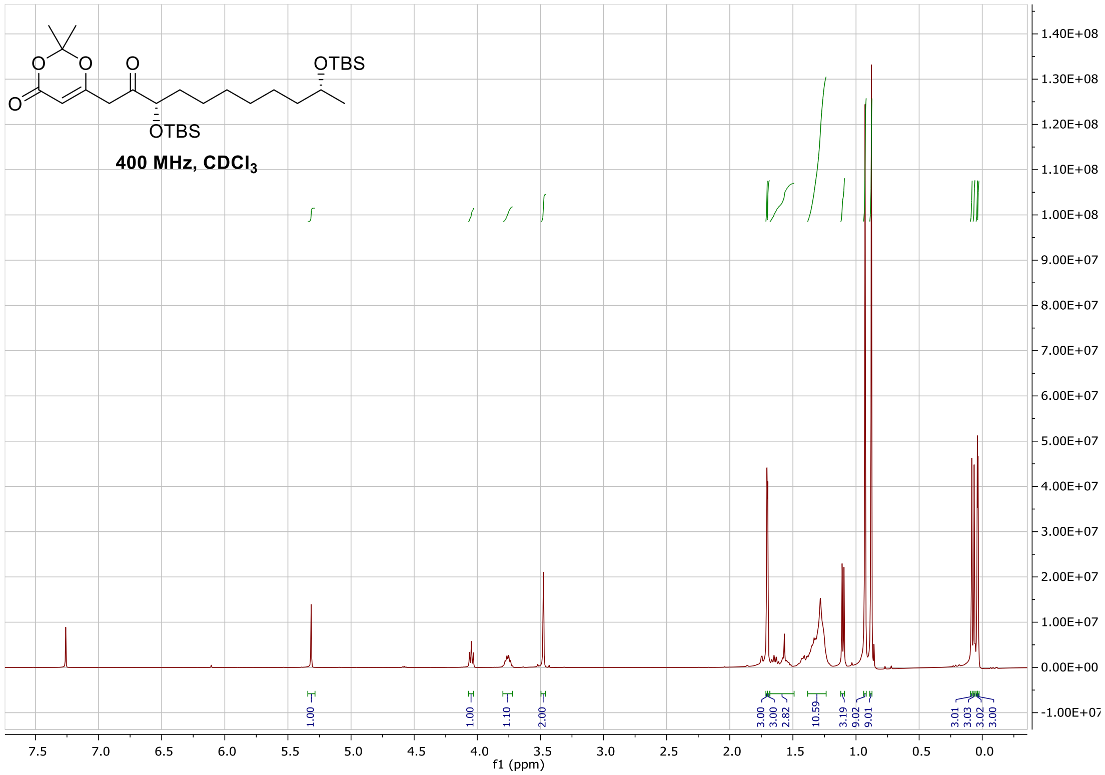


**Figure S-12** $^{1}$H-NMR spectrum of compound **19**.

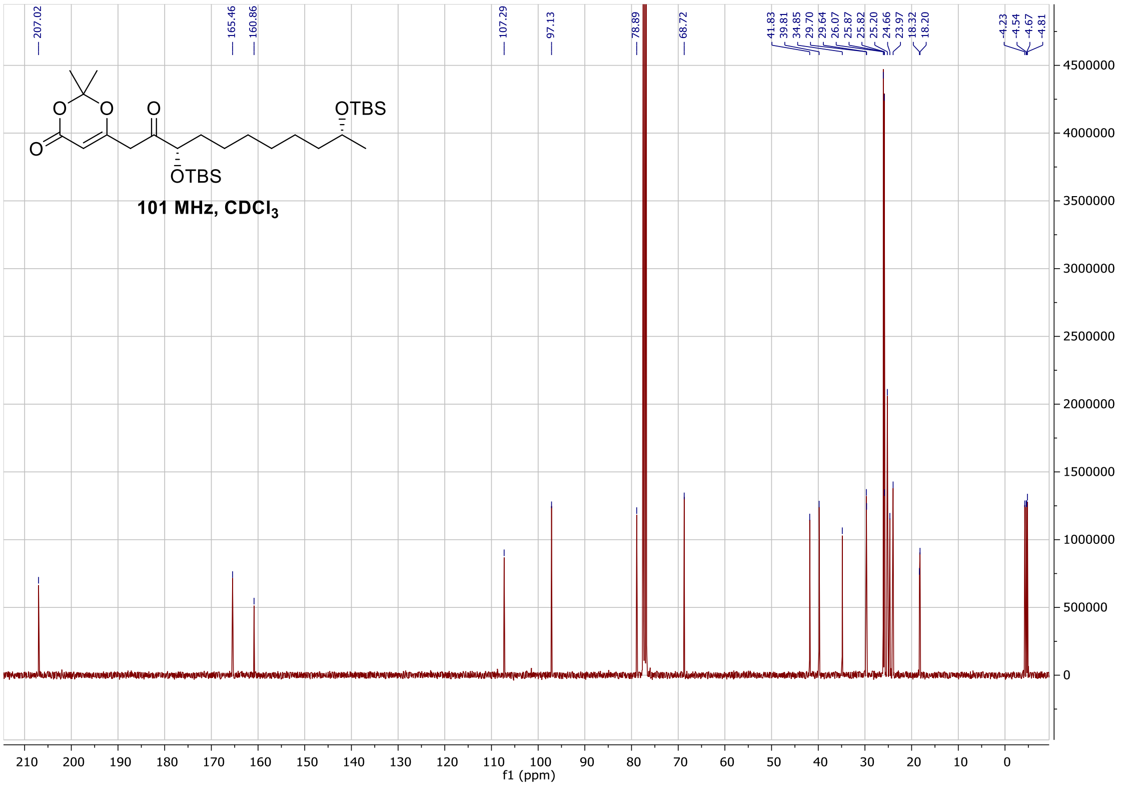


**Figure S-13** $^{13}$C-NMR spectrum of compound **19**.

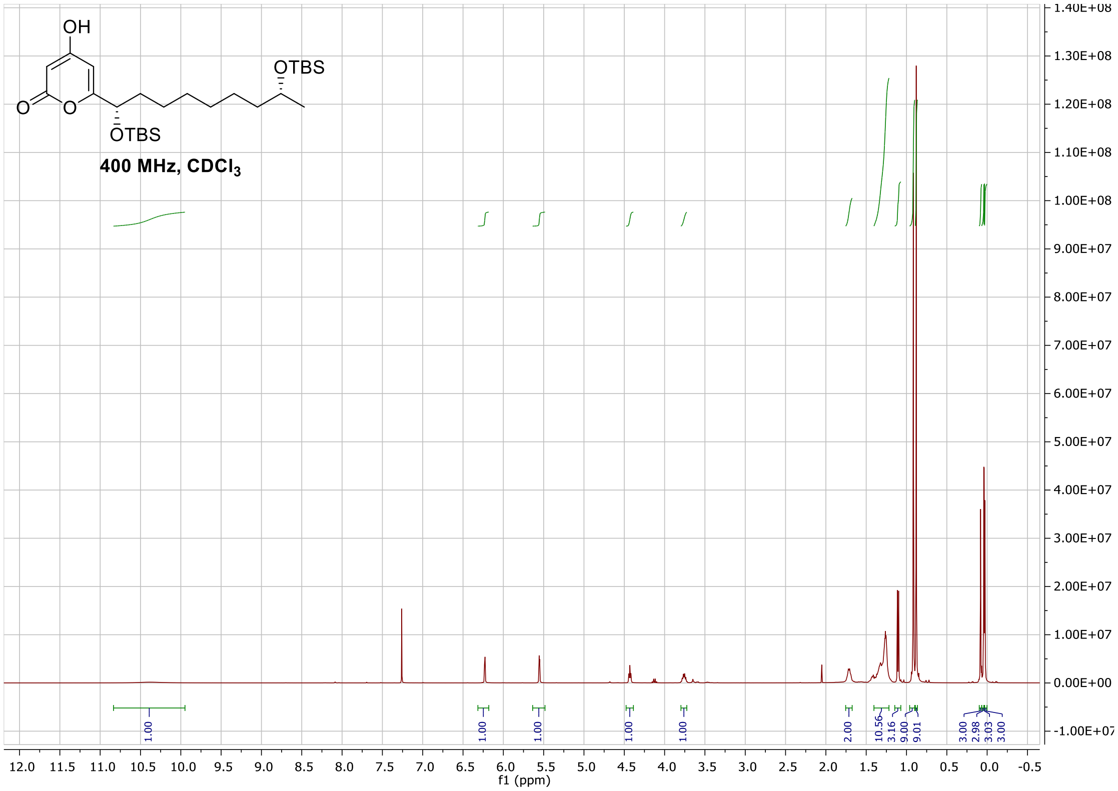


**Figure S-14** $^{1}$H-NMR spectrum of compound **20**.

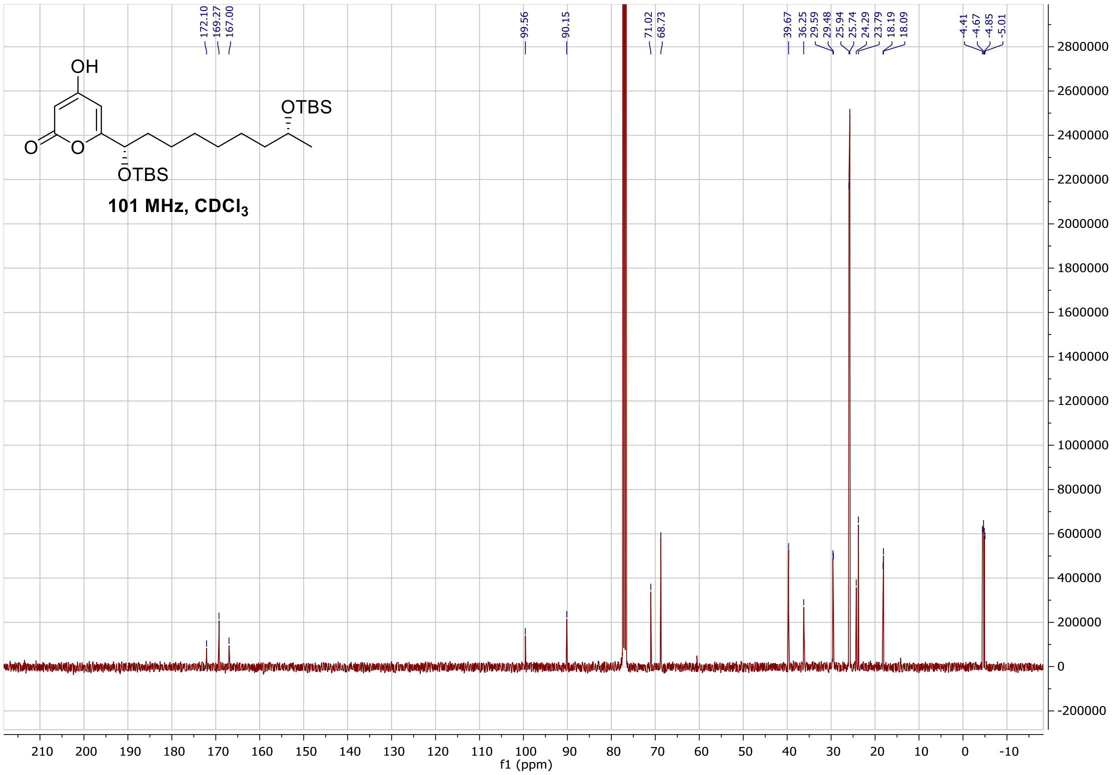


**Figure S-15** $^{13}$C-NMR spectrum of compound **20**.

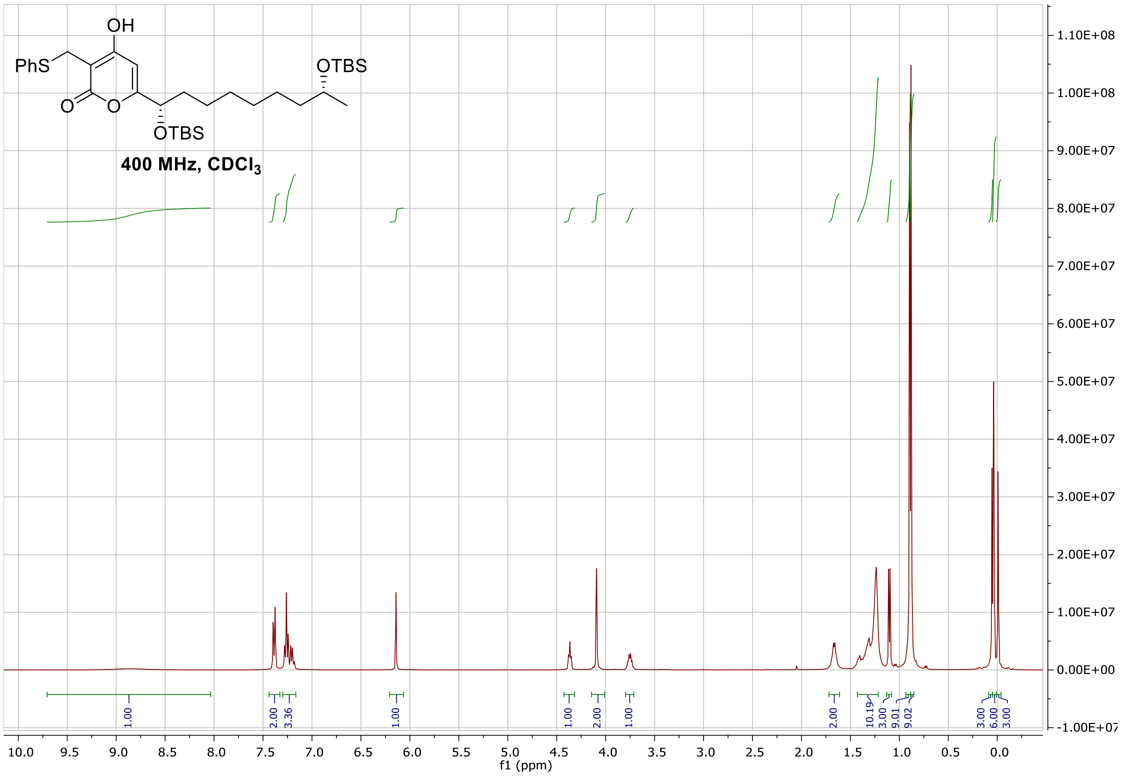


**Figure S-16** $^{1}$H-NMR spectrum of compound **21**.

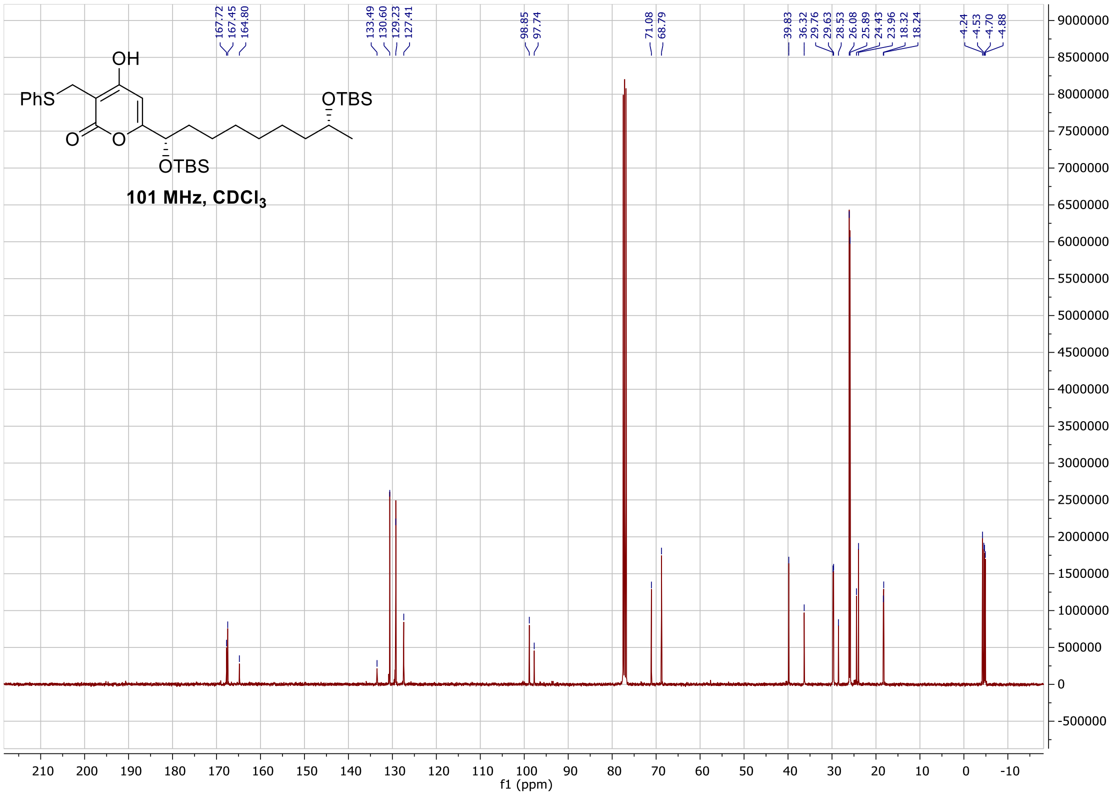


**Figure S-17** $^{13}$C-NMR spectrum of compound **21**.

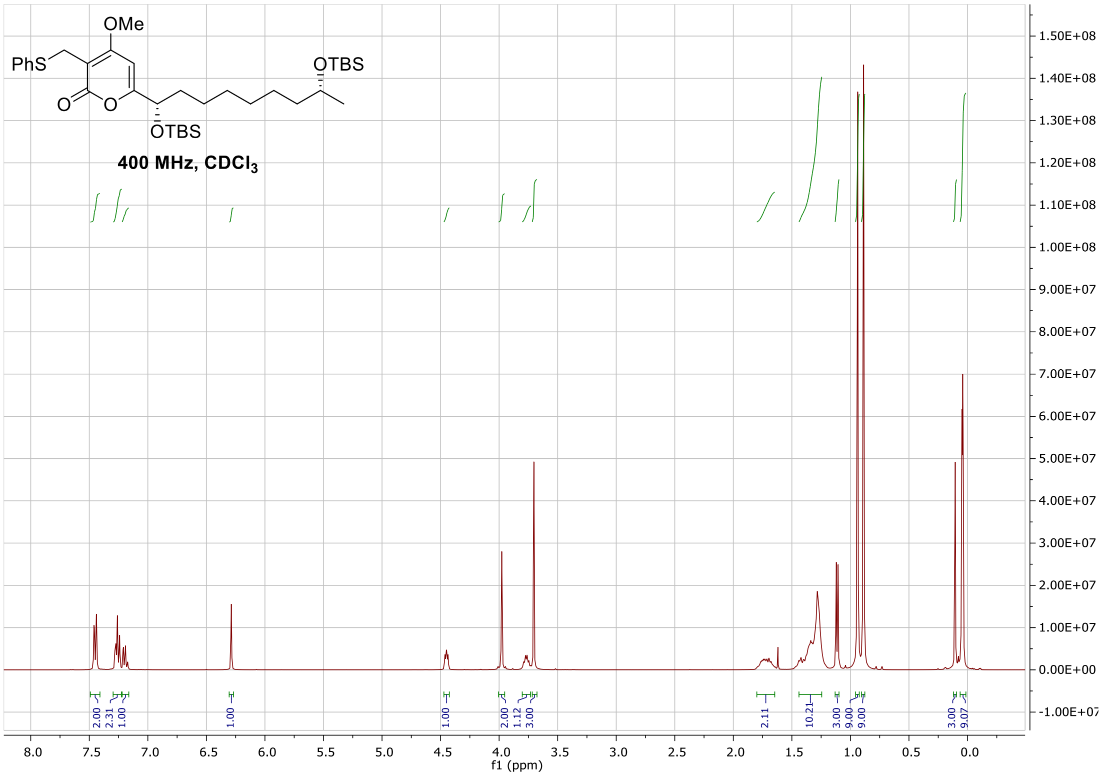


**Figure S-18** $^{1}$H-NMR spectrum of compound **22**.

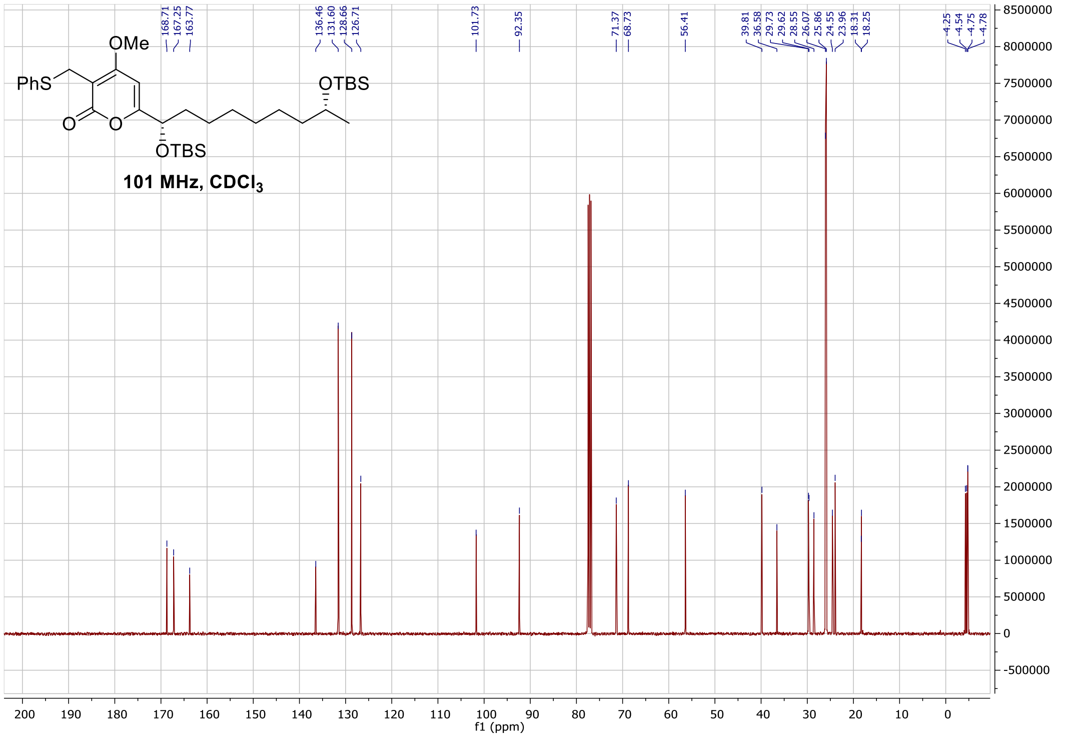


**Figure S-19** $^{13}$C-NMR spectrum of compound **22**.

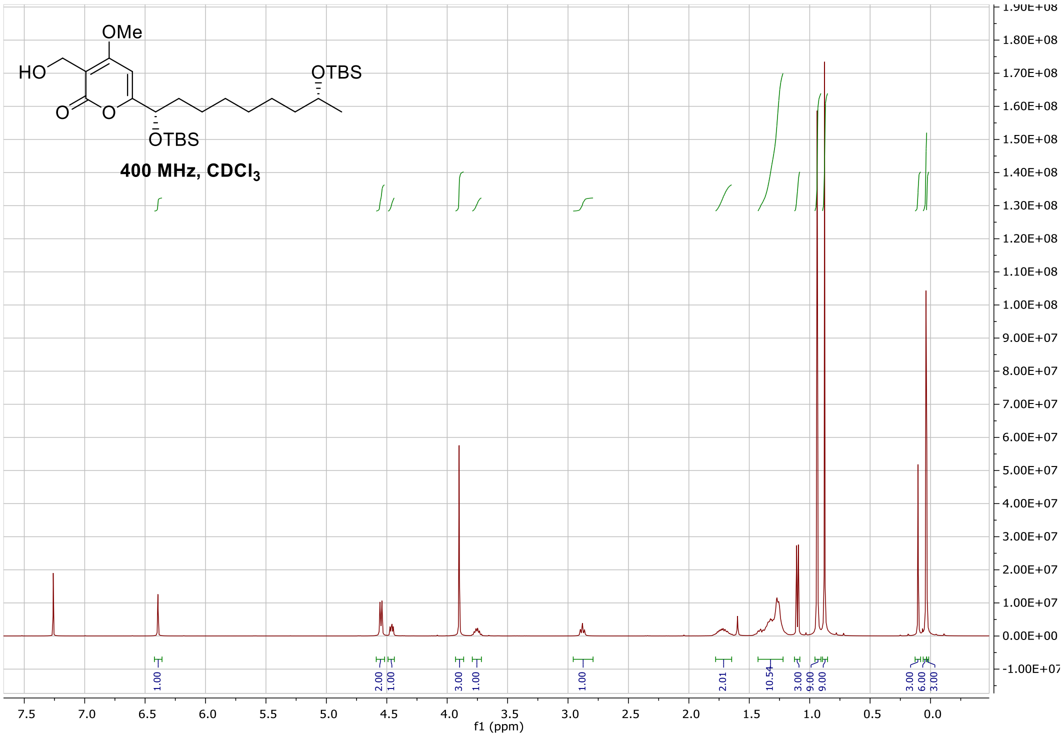


**Figure S-20** $^{1}$H-NMR spectrum of compound **24**.

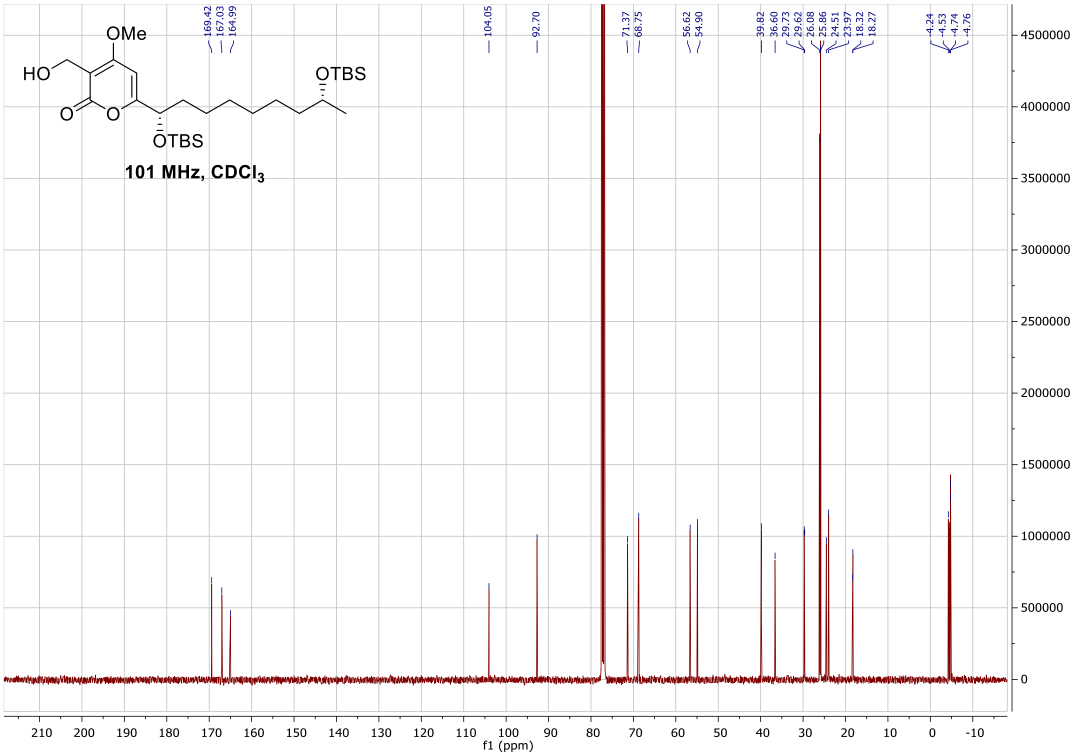


**Figure S-21** $^{13}$C-NMR spectrum of compound **24**.

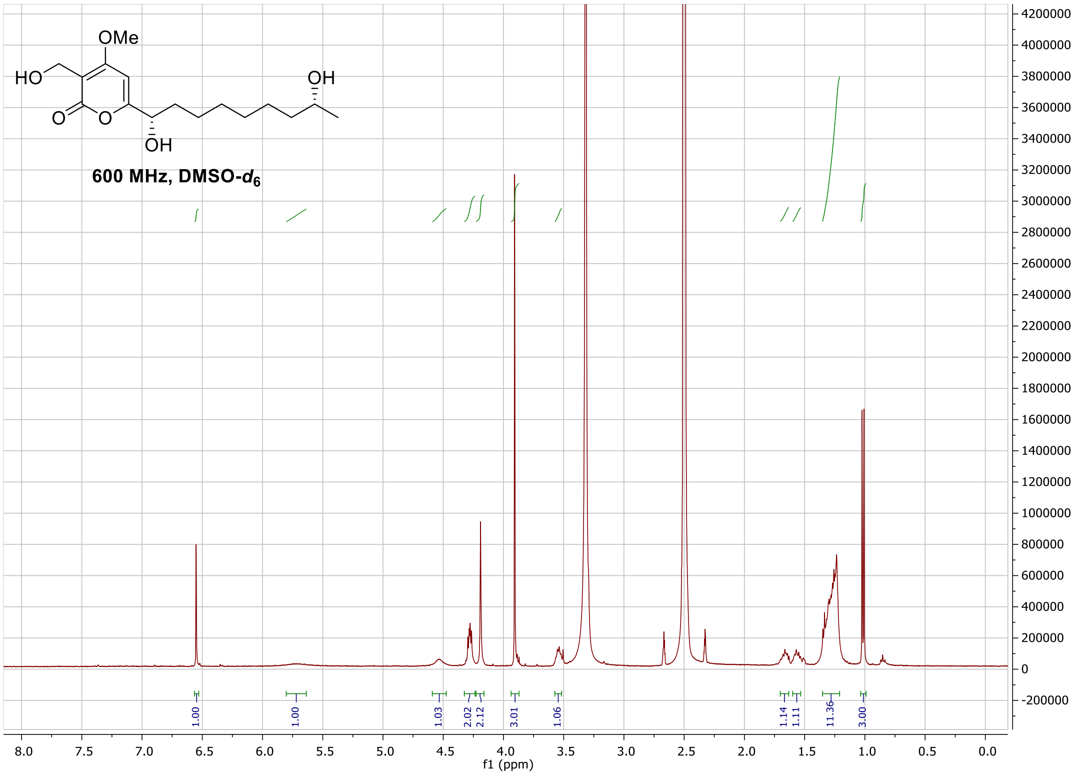


**Figure S-22** $^{1}$H-NMR spectrum of dothideopyrone E (**1**).

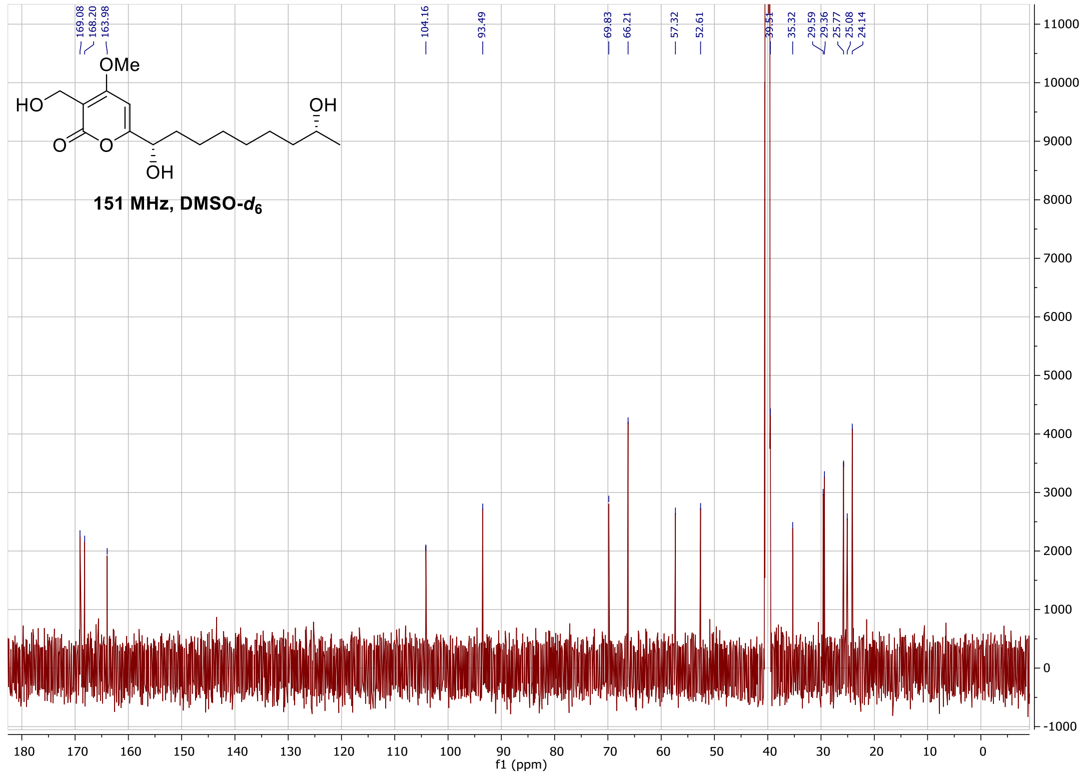


**Figure S-23** $^{13}$C-NMR spectrum of dothideopyrone E (**1**).

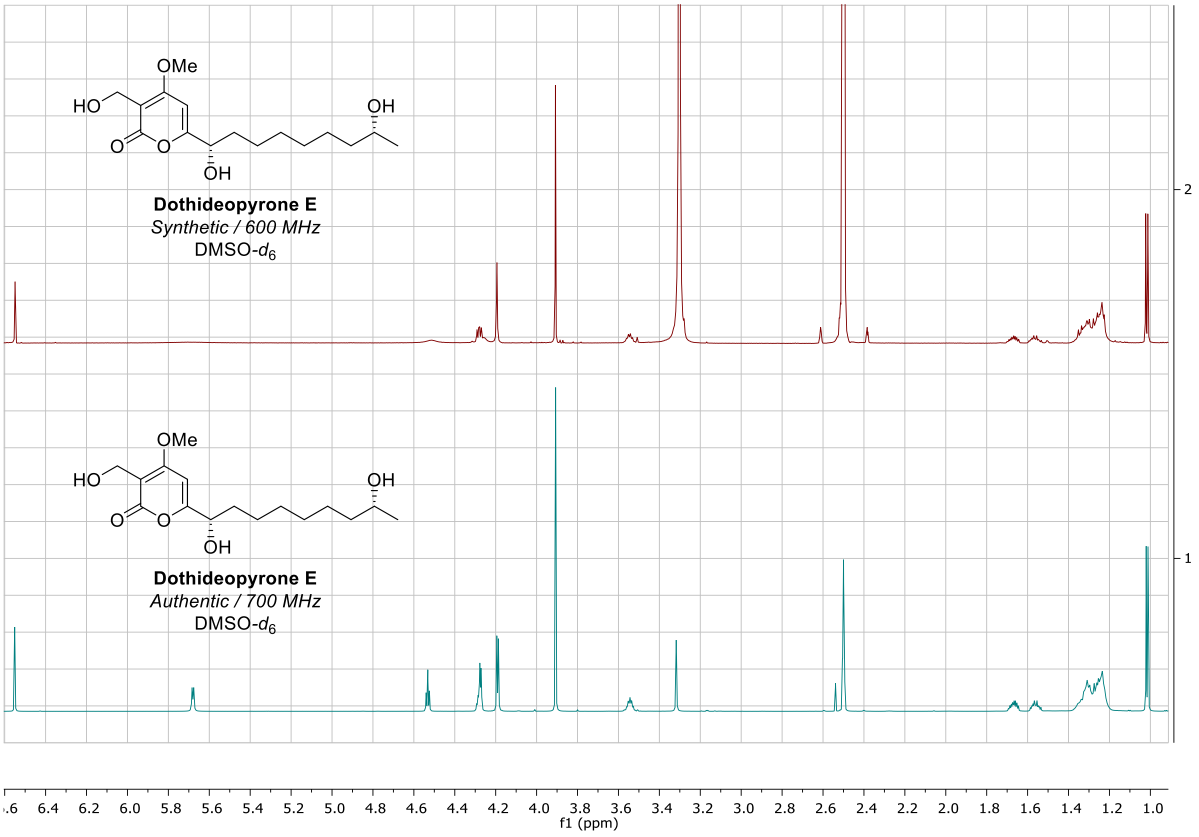


**Figure S-24** Direct comparison between the $^{1}$H-NMR spectrum of compound synthetic and authentic dothideopyrone E (**1**) in DMSO-$d_6$.

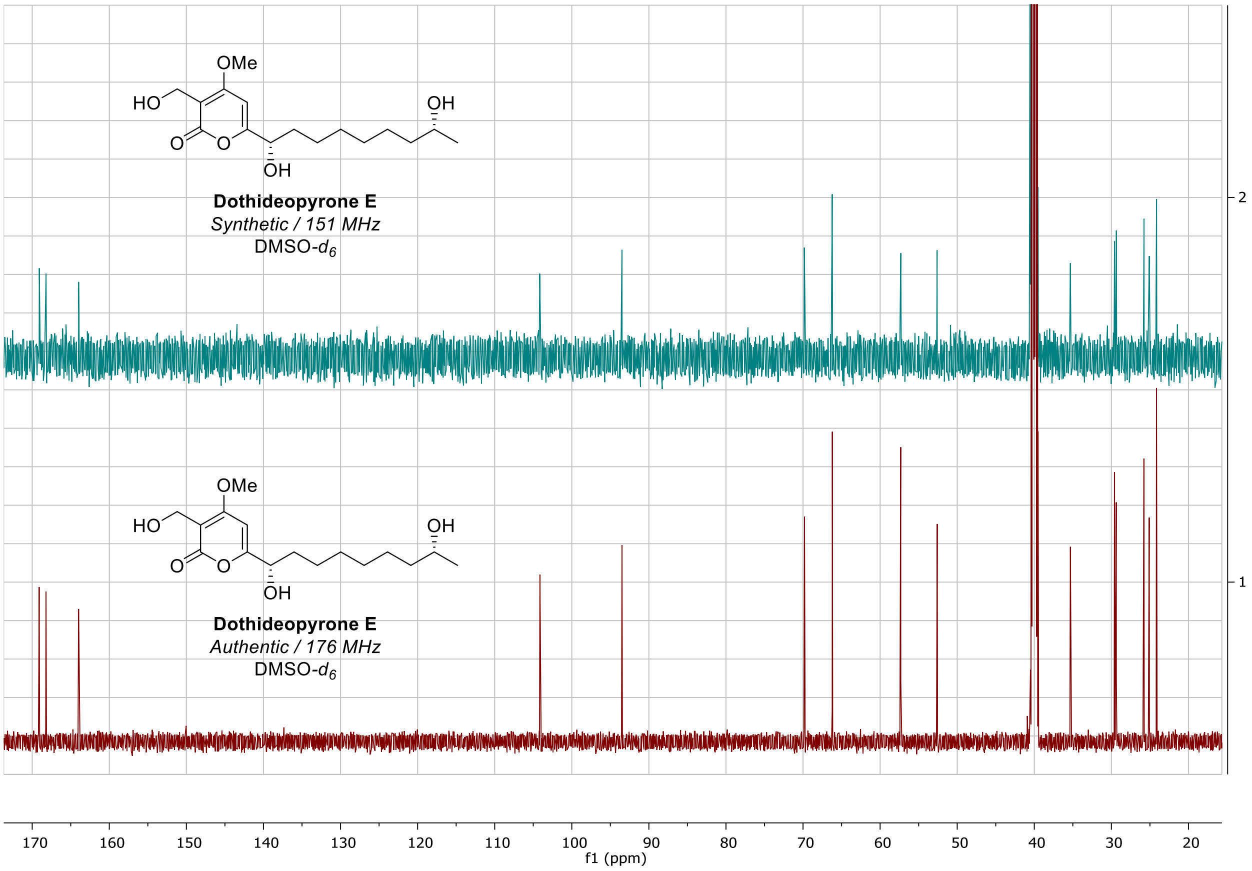


**Figure S-25** Direct comparison between $^{13}$C-NMR spectrum of compound synthetic and authentic dothideopyrone E (**1**) in DMSO-$d_6$.

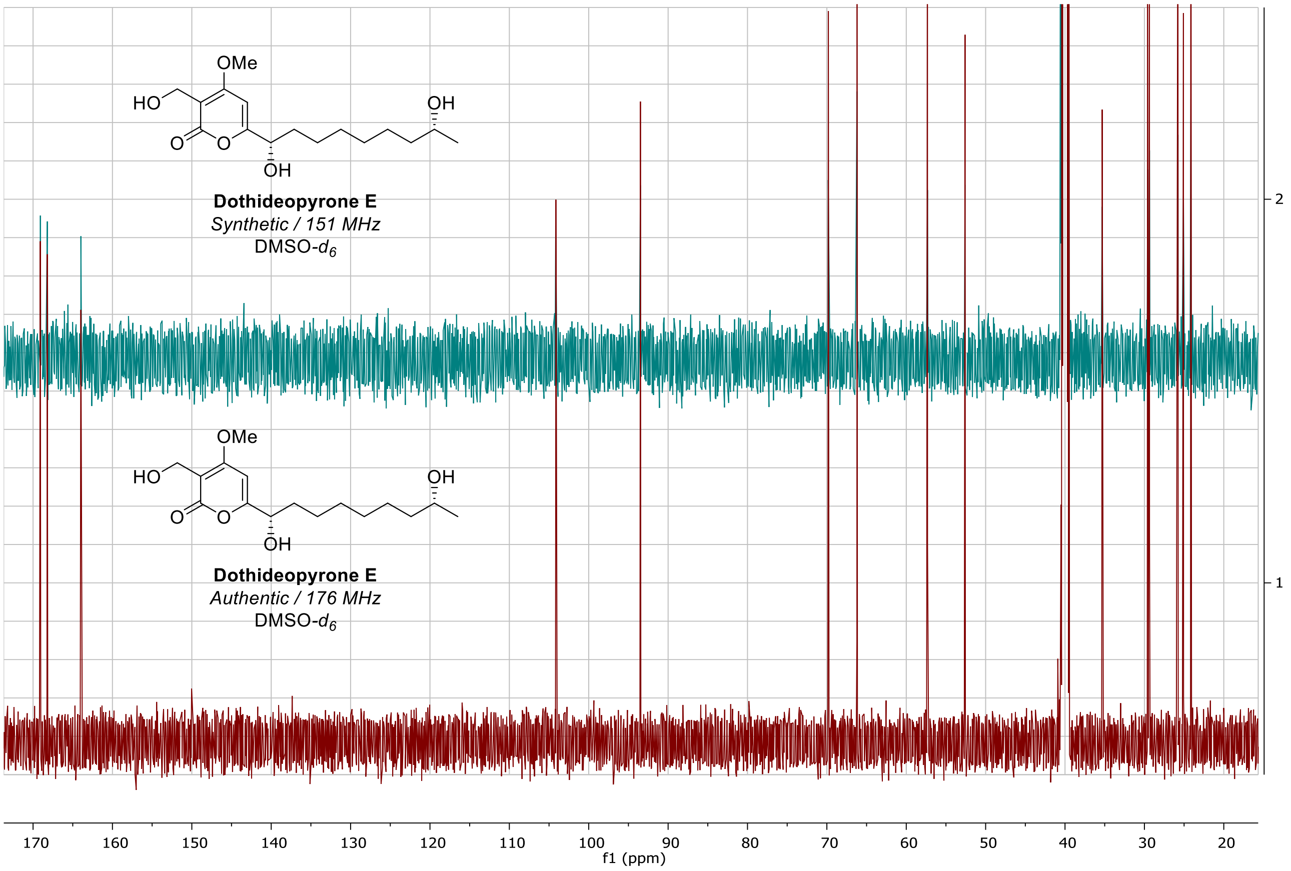


**Figure S-26** Direct comparison between $^{13}$C-NMR spectrum of compound synthetic and authentic dothideopyrone E (**1**) in DMSO-$d_6$ – increased intensity.

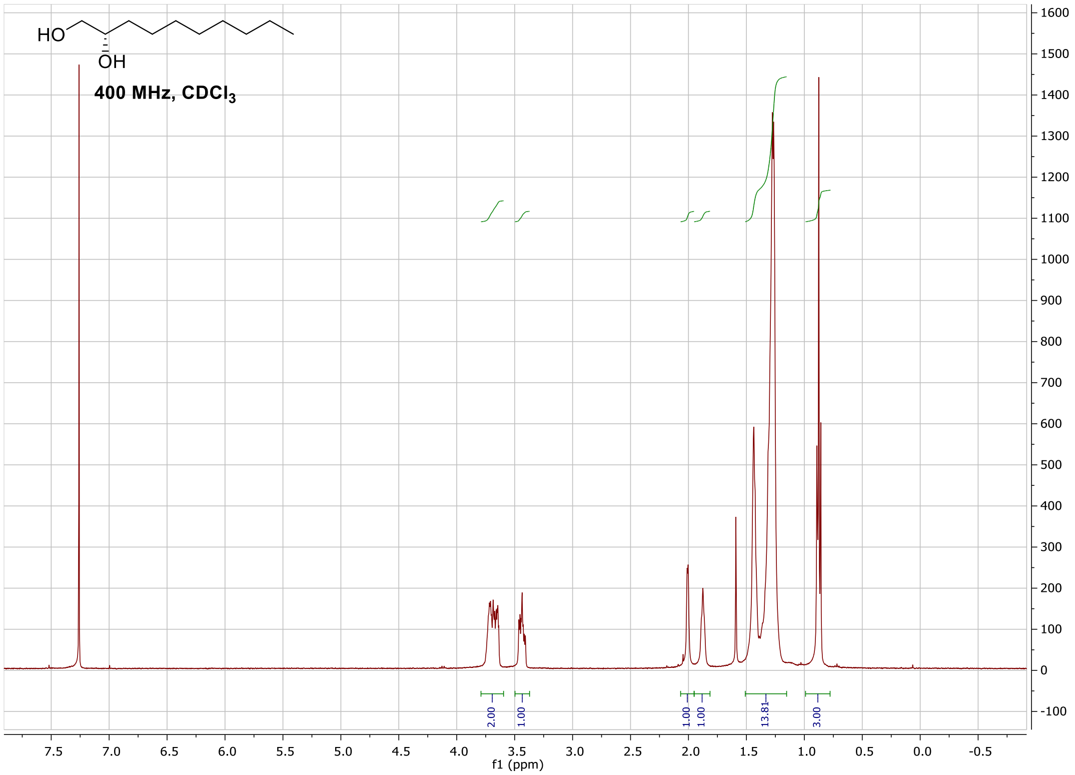


**Figure S-27** $^{1}$H-NMR spectrum of compound **26**.

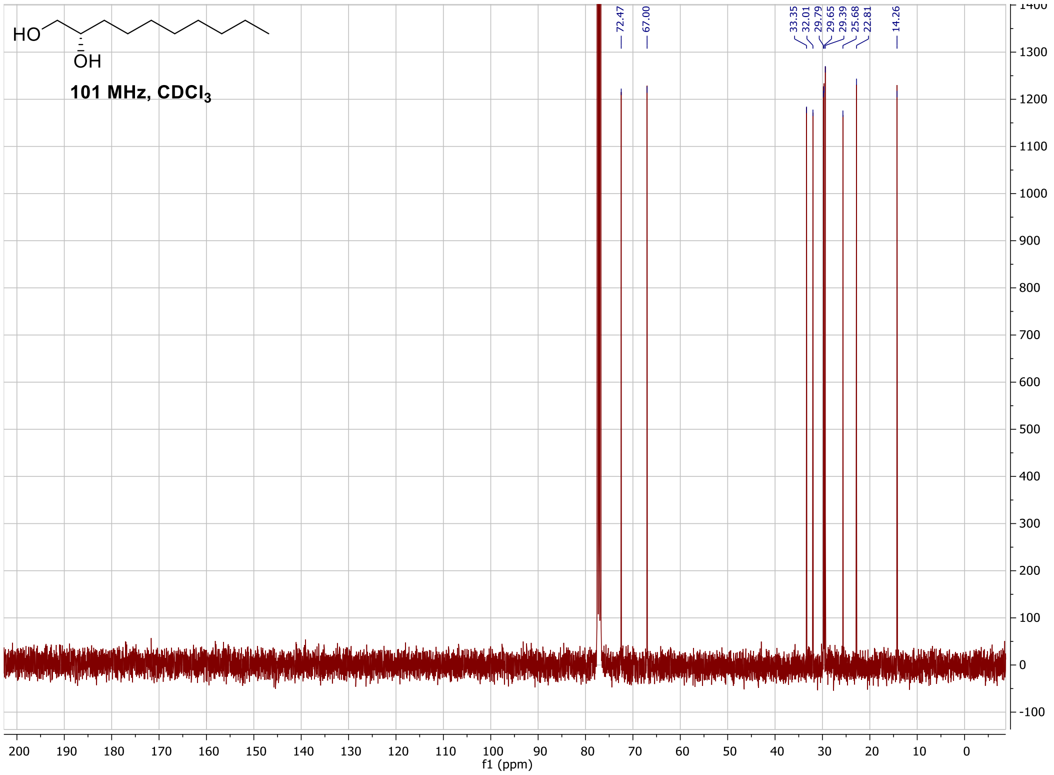


**Figure S-28** $^{13}$C-NMR spectrum of compound **26**.

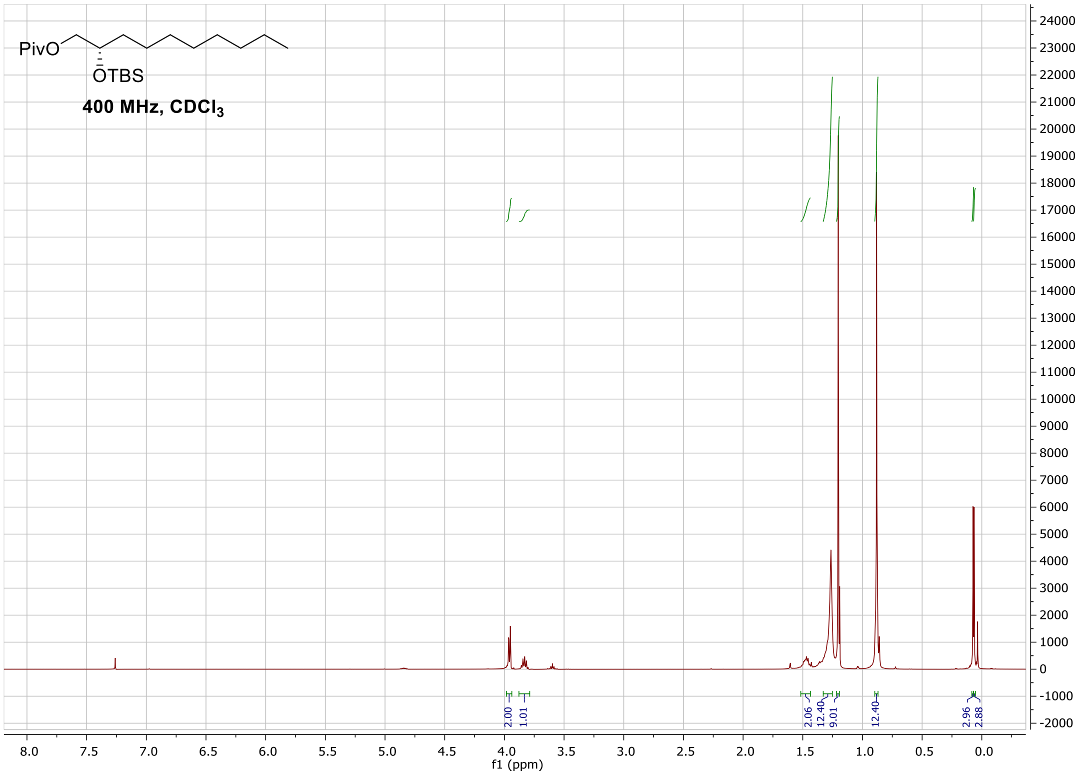


**Figure S-29** $^{1}$H-NMR spectrum of compound **S2**.

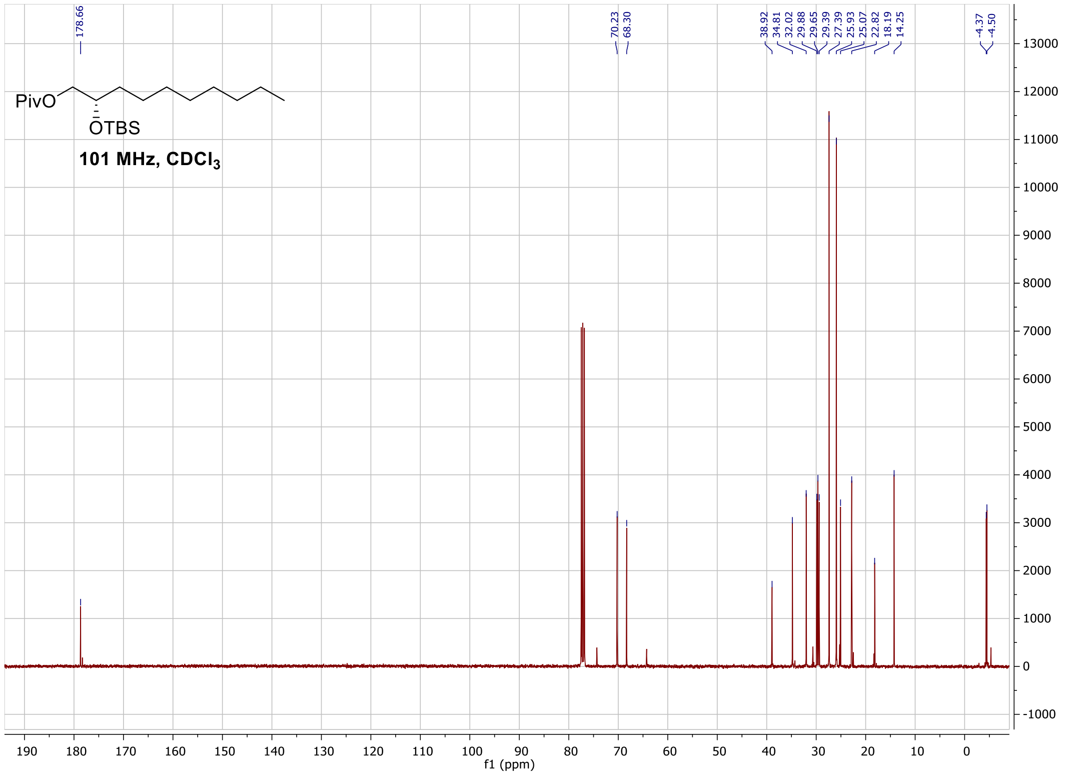


**Figure S-30** $^{13}$C-NMR spectrum of compound **S2**.

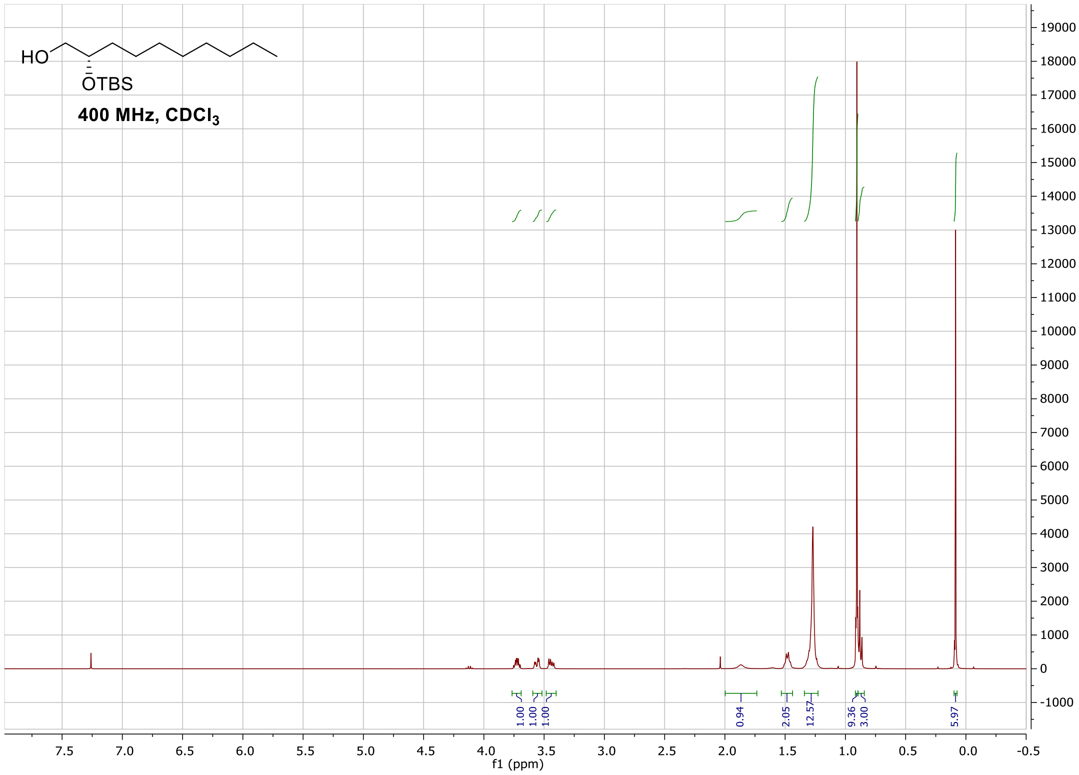


**Figure S-31** $^{1}$H-NMR spectrum of compound **S3**.

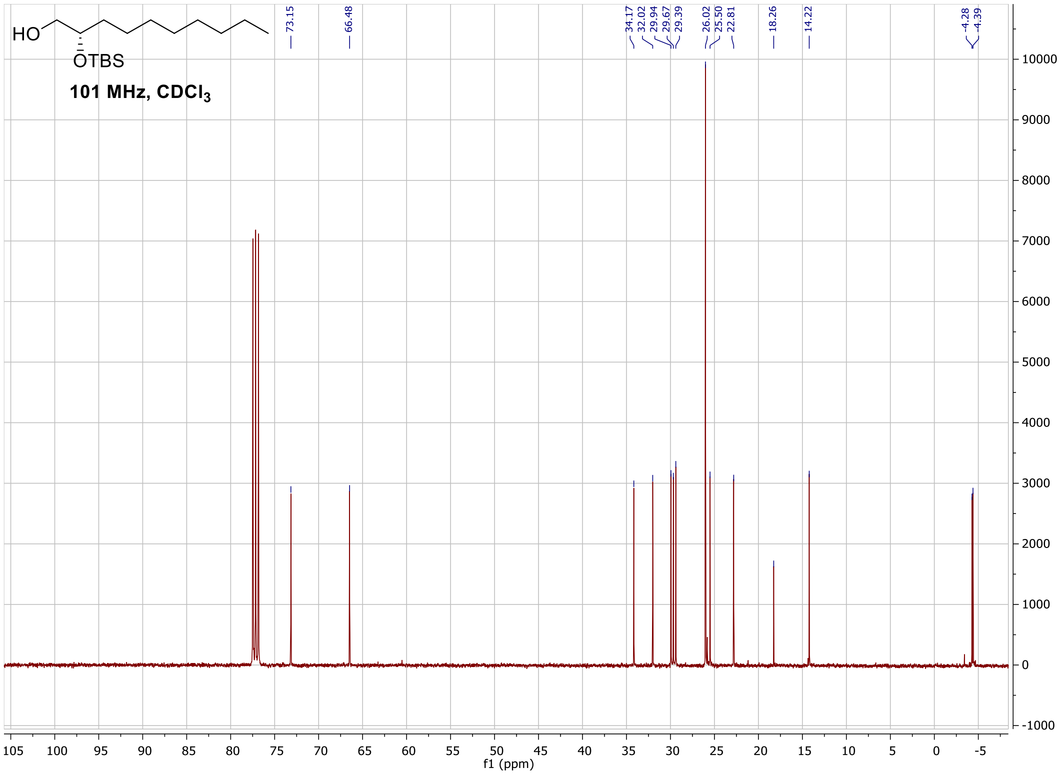


**Figure S-32** $^{13}$C-NMR spectrum of compound **S3**.

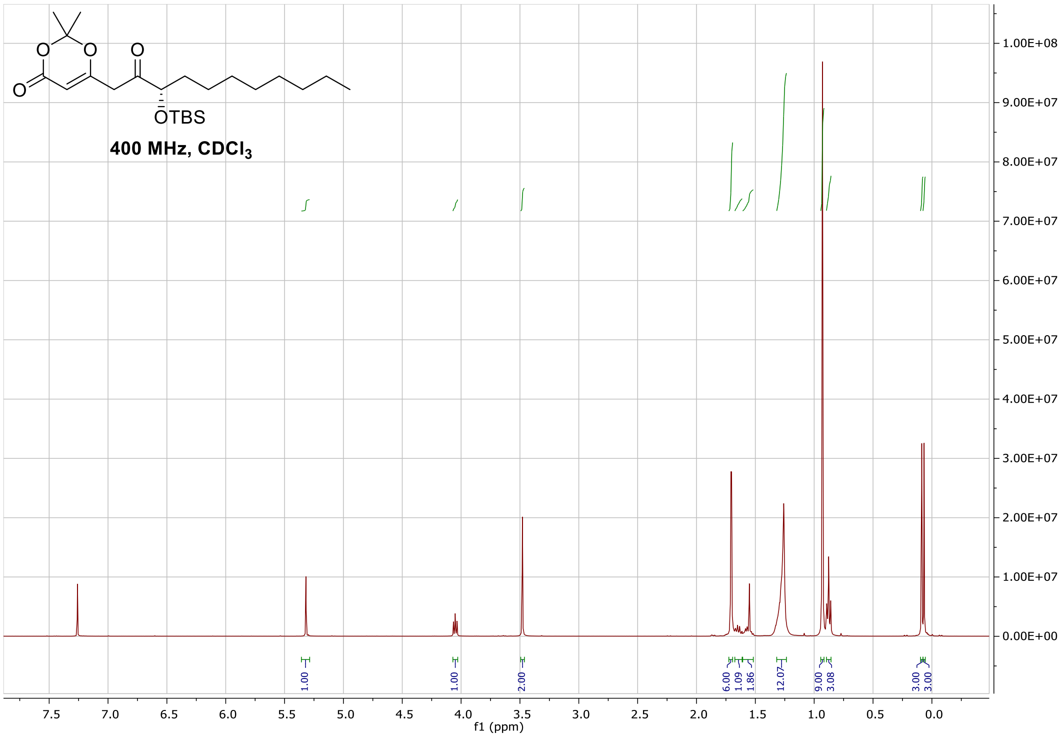


**Figure S-33** $^{1}$H-NMR spectrum of compound **S6**.

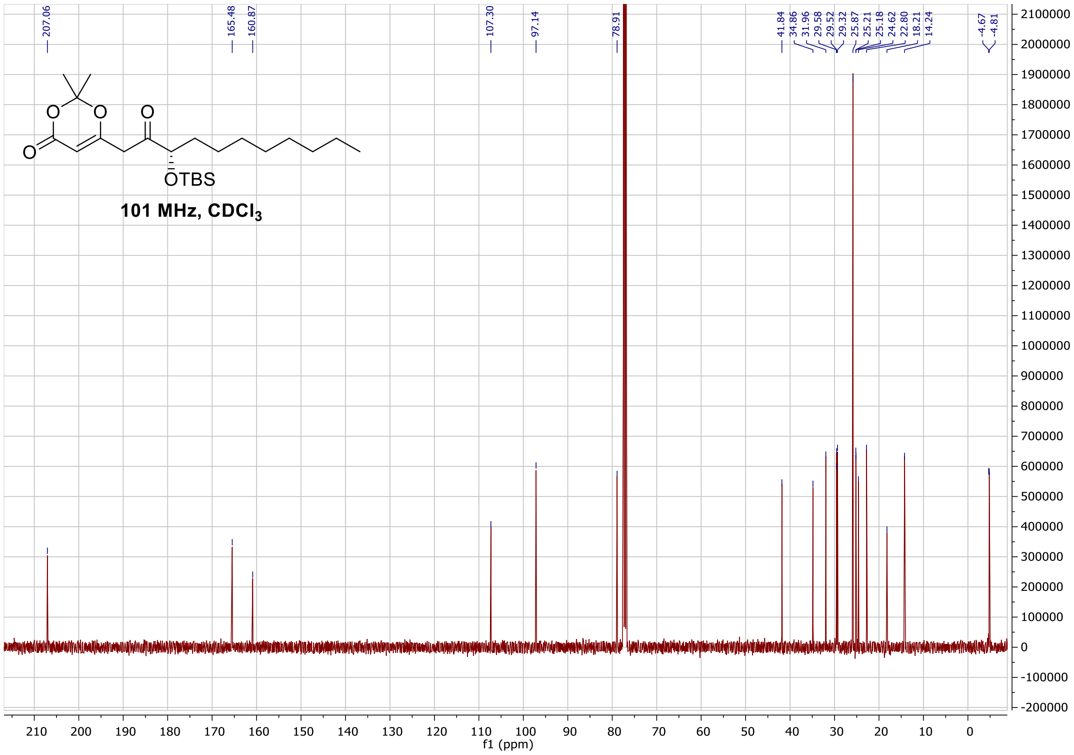


**Figure S-34** $^{13}$C-NMR spectrum of compound **S6**.

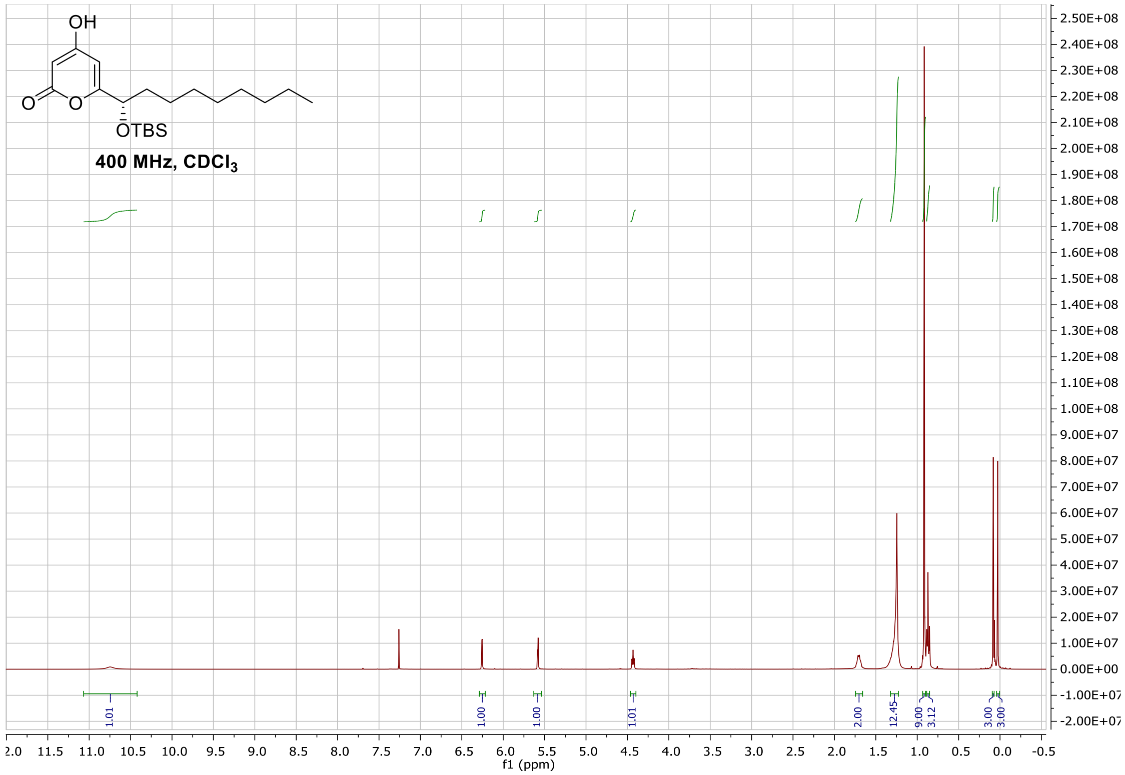


**Figure S-35** $^{1}$H-NMR spectrum of compound **S7**.

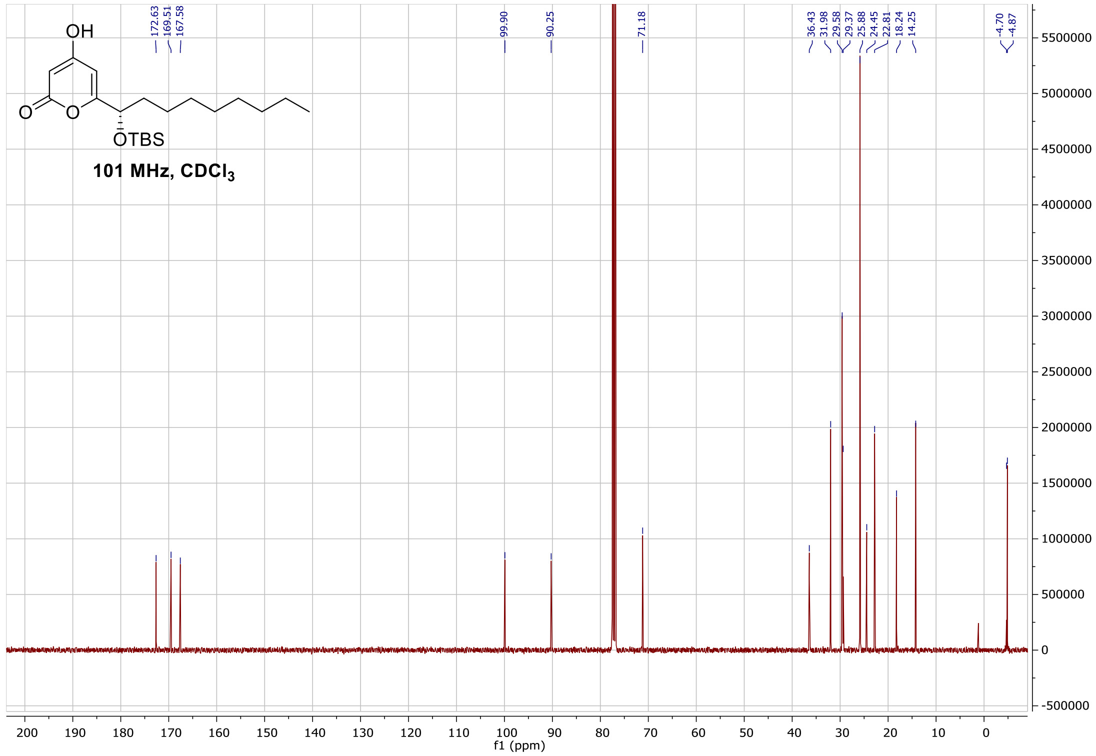


**Figure S-36** $^{13}$C-NMR spectrum of compound **S7**.

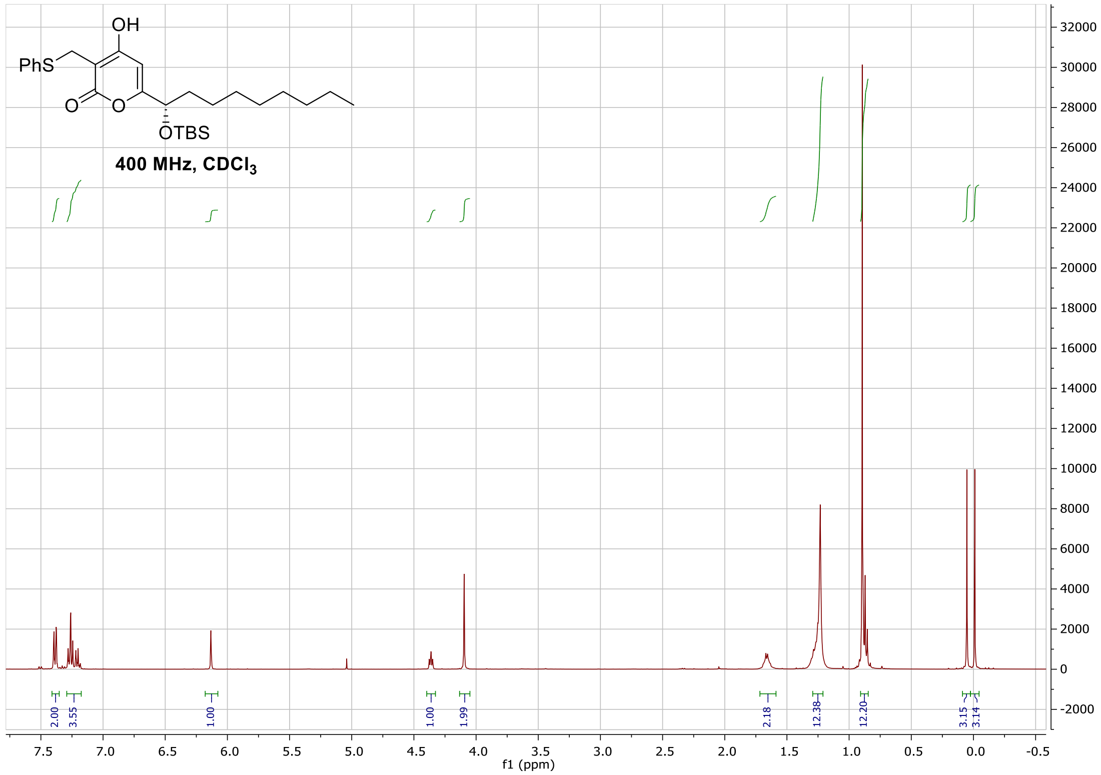

**Figure S-37** $^{1}$H-NMR spectrum of compound **S8**.

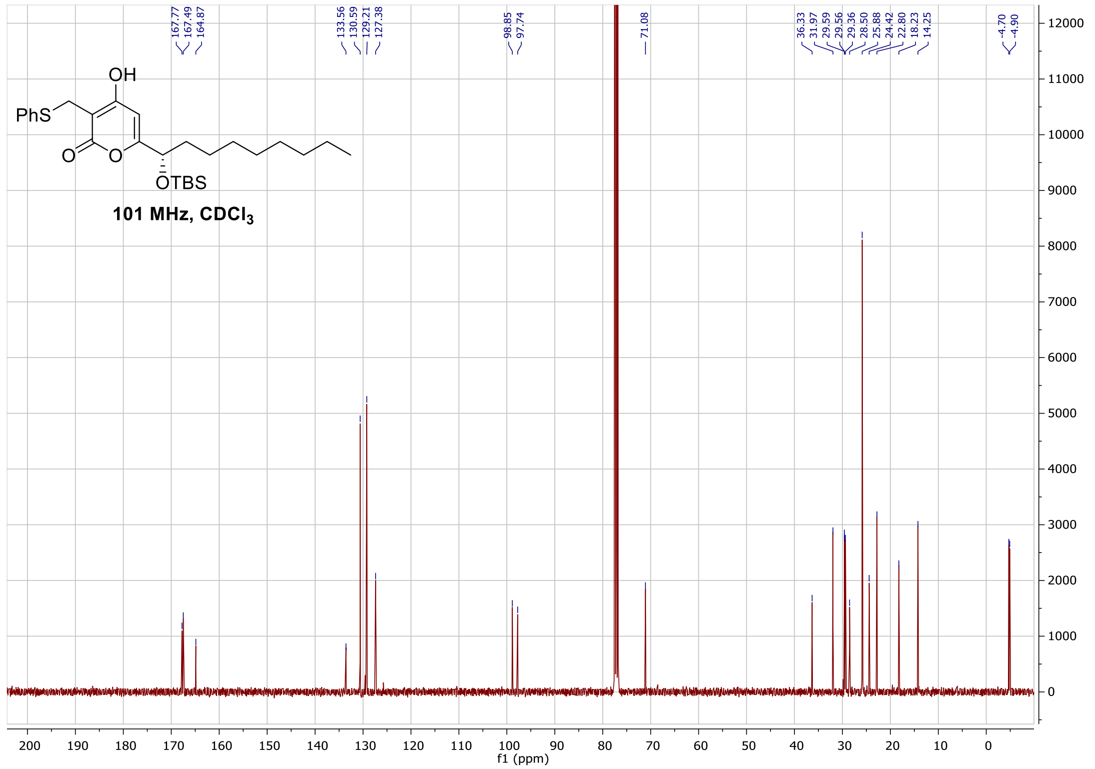

**Figure S-38** $^{13}$C-NMR spectrum of compound **S8**.

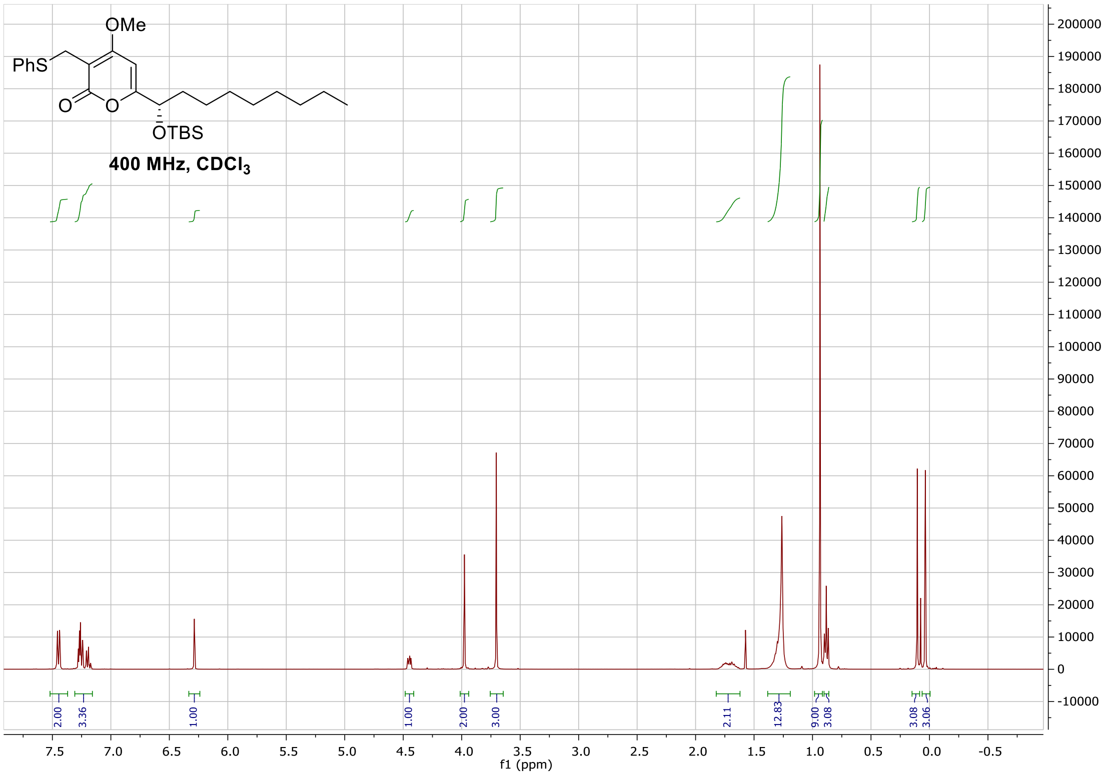


**Figure S-39** $^{1}$H-NMR spectrum of compound **S9**.

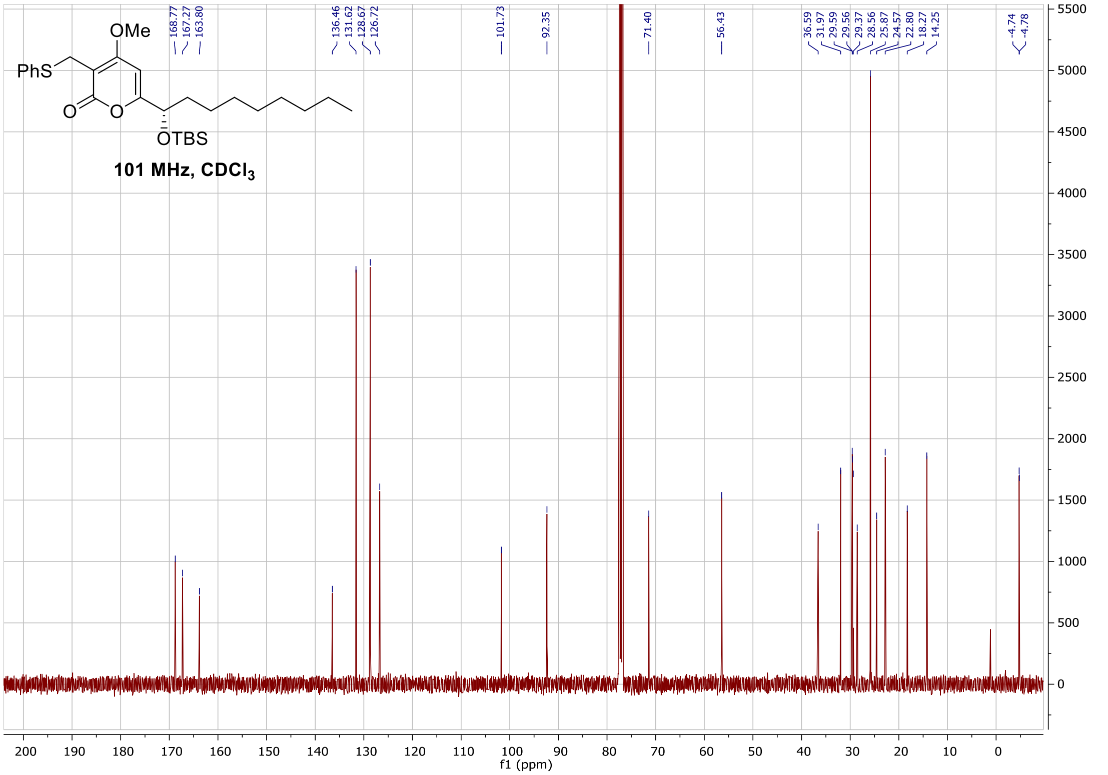


**Figure S-40** $^{13}$C-NMR spectrum of compound **S9**.

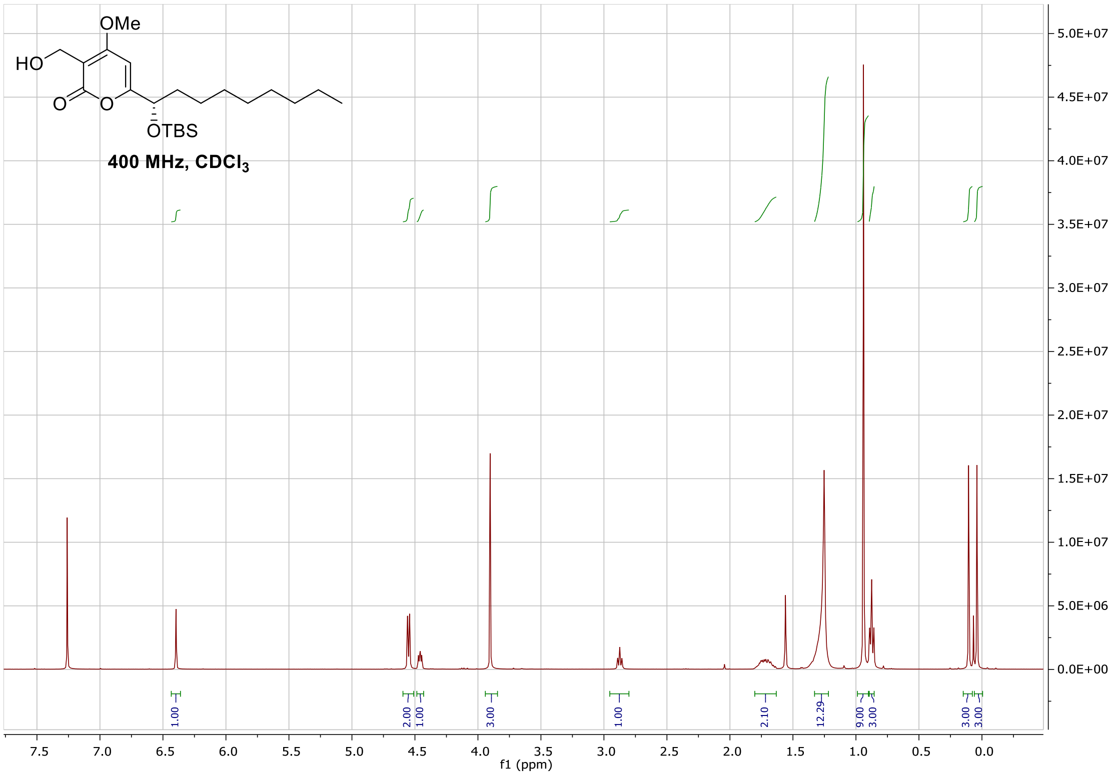


**Figure S-41** $^{1}$H-NMR spectrum of compound **S11**.

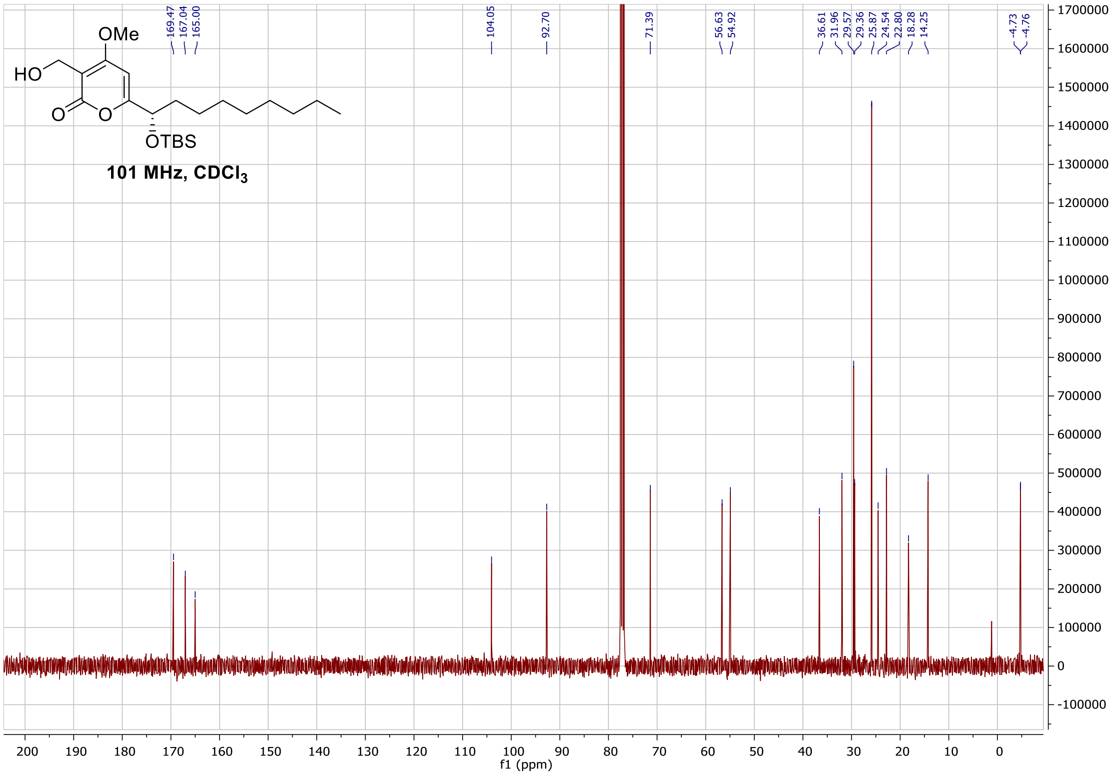


**Figure S-42** $^{13}$C-NMR spectrum of compound **S11**.

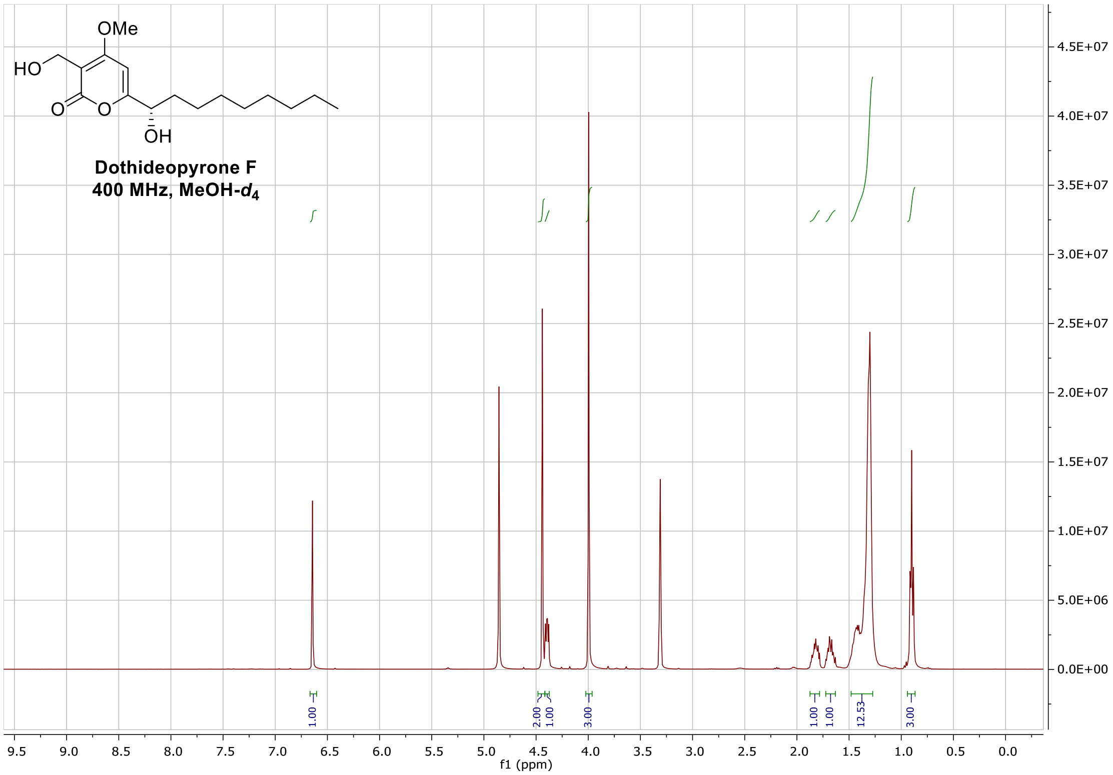


**Figure S-43** $^{1}$H-NMR spectrum of dothideopyrone F (**2**).

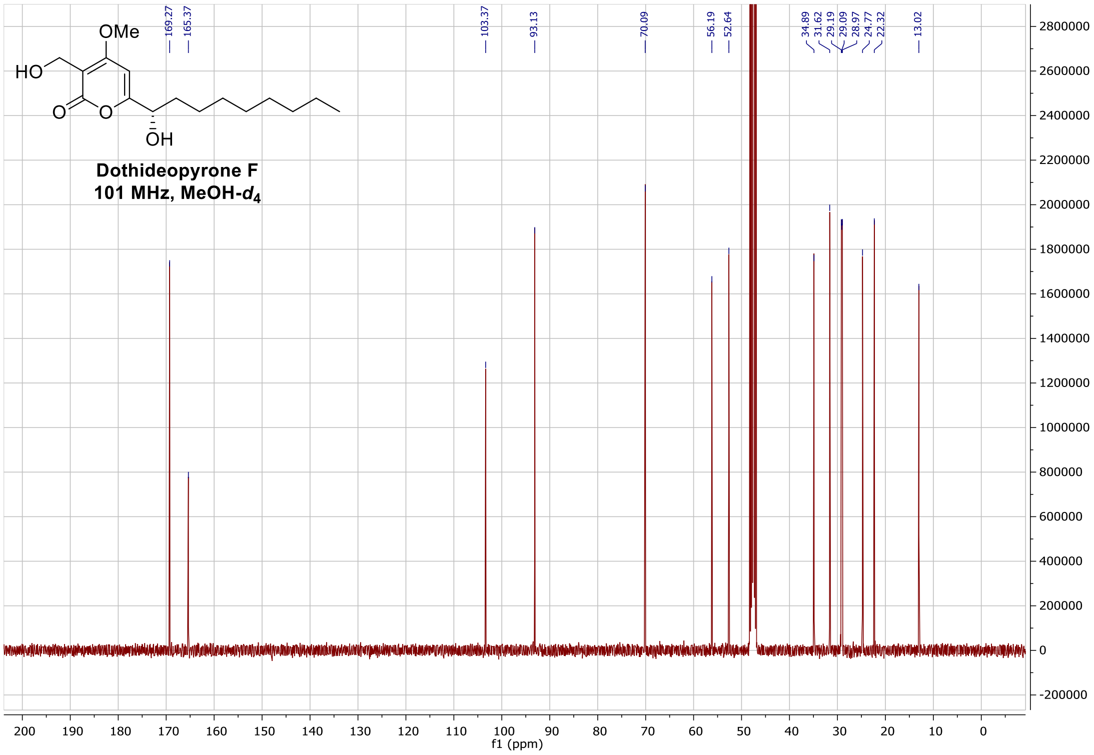


**Figure S-44** $^{13}$C-NMR spectrum of dothideopyrone F (**2**).

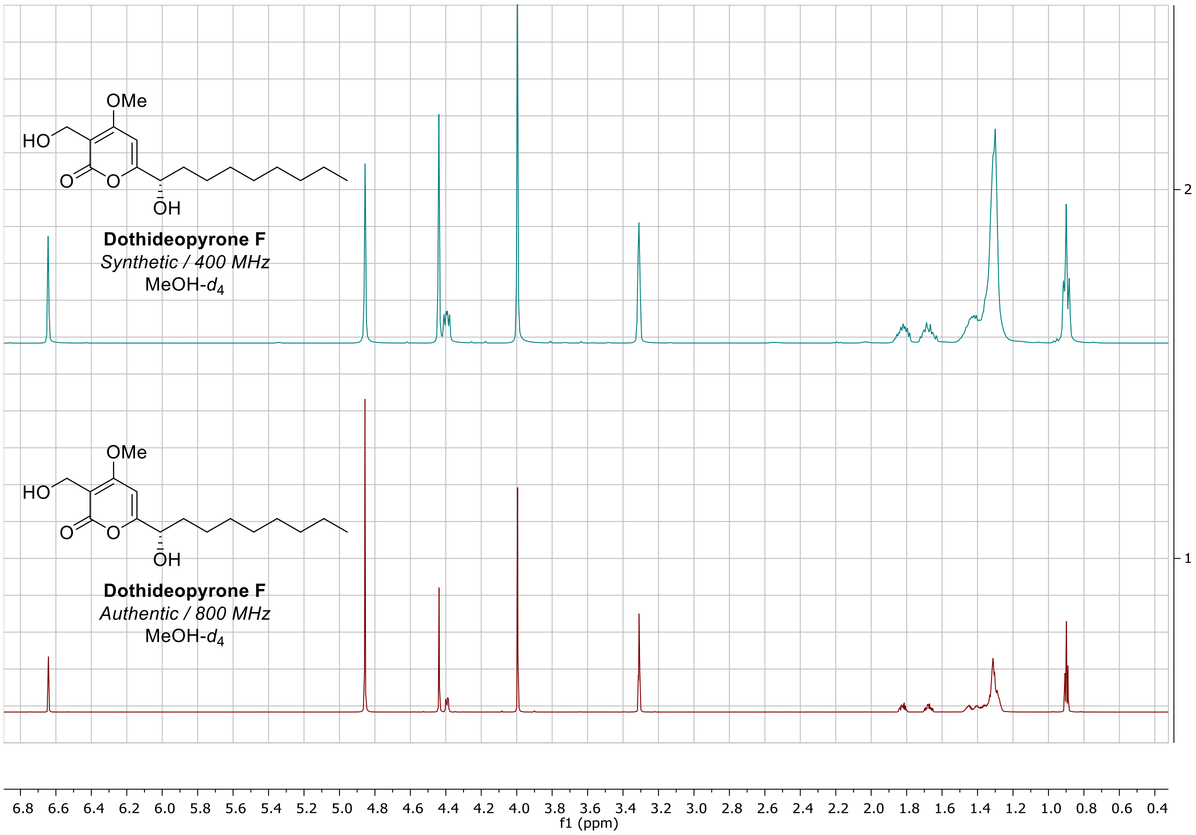


**Figure S-45** Direct comparison between the $^{1}$H-NMR spectrum of compound synthetic and authentic dothideopyrone F (**2**) in MeOH-$d_4$.

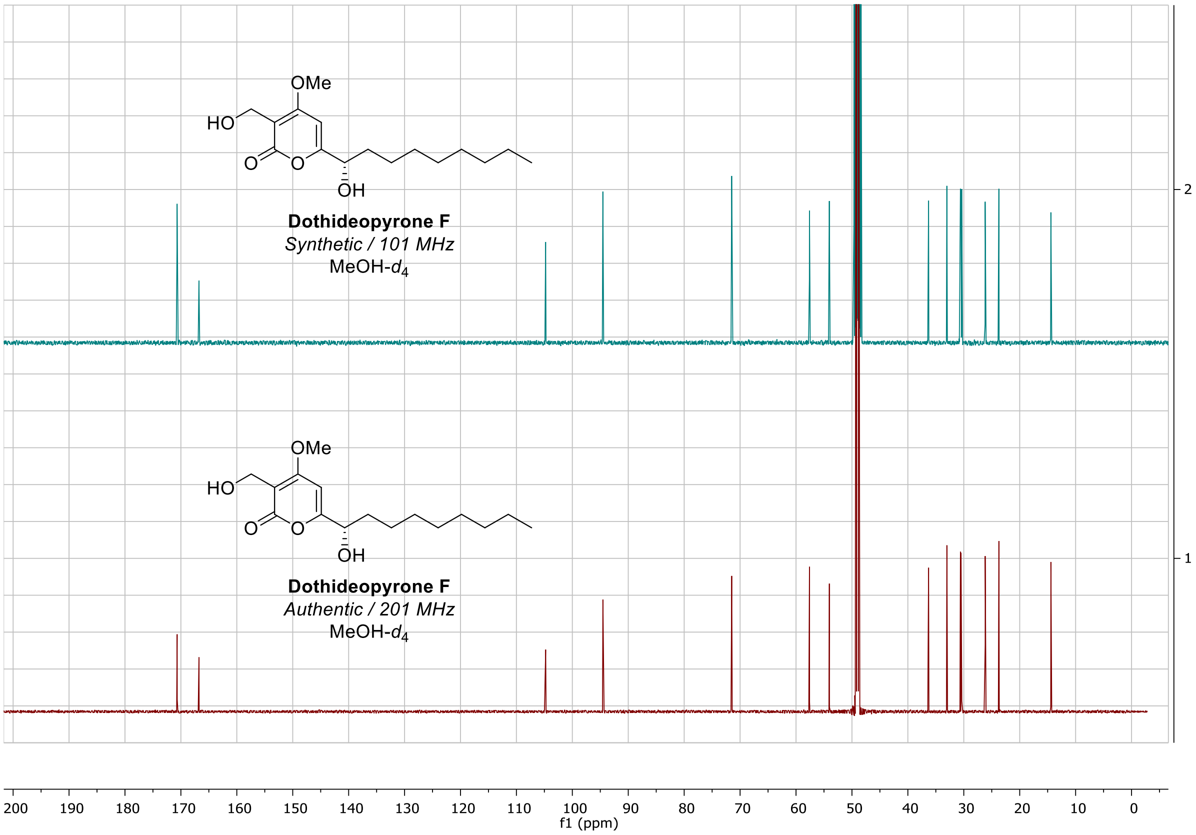


**Figure S-46** Direct comparison between the $^{13}$C-NMR spectrum of compound synthetic and authentic dothideopyrone F (**2**) in MeOH-$d_4$.

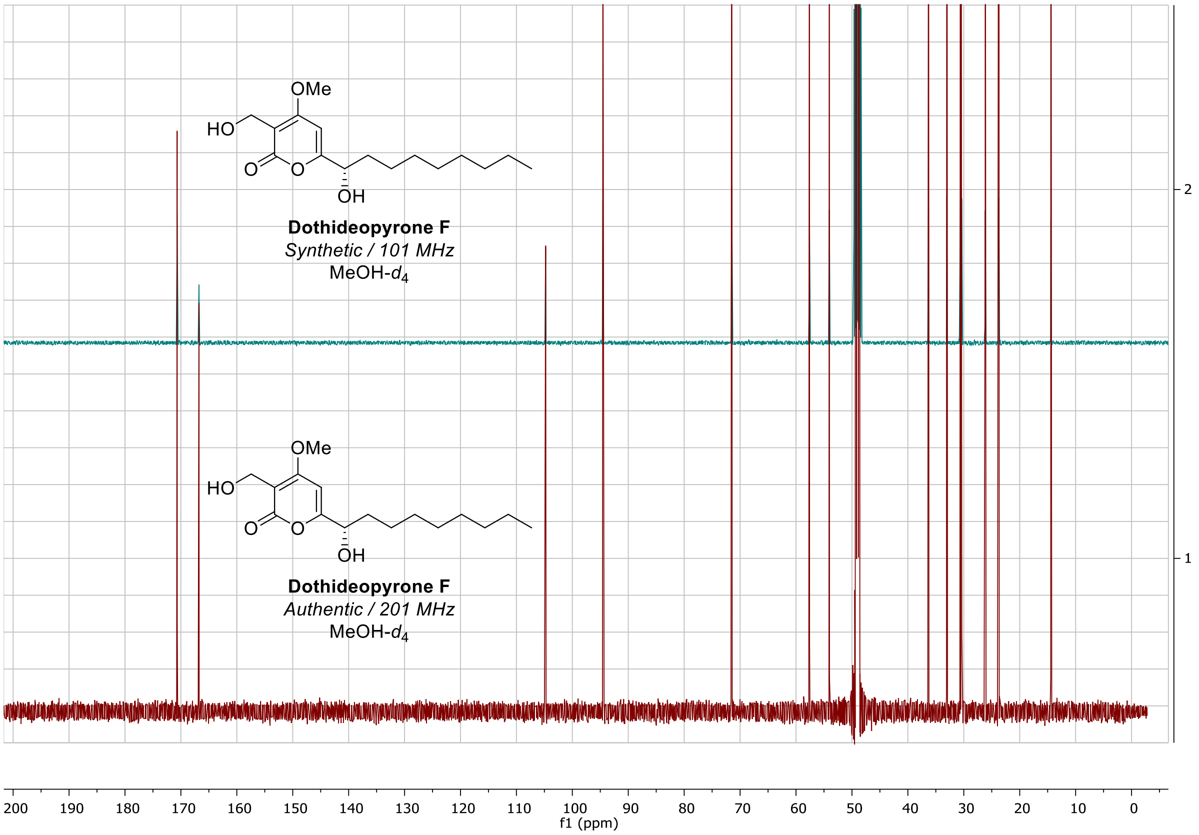


**Figure S-47** Direct comparison between $^{13}$C-NMR spectrum of compound synthetic and authentic dothideopyrone F (**2**) in MeOH-$d_4$ – increased intensity.

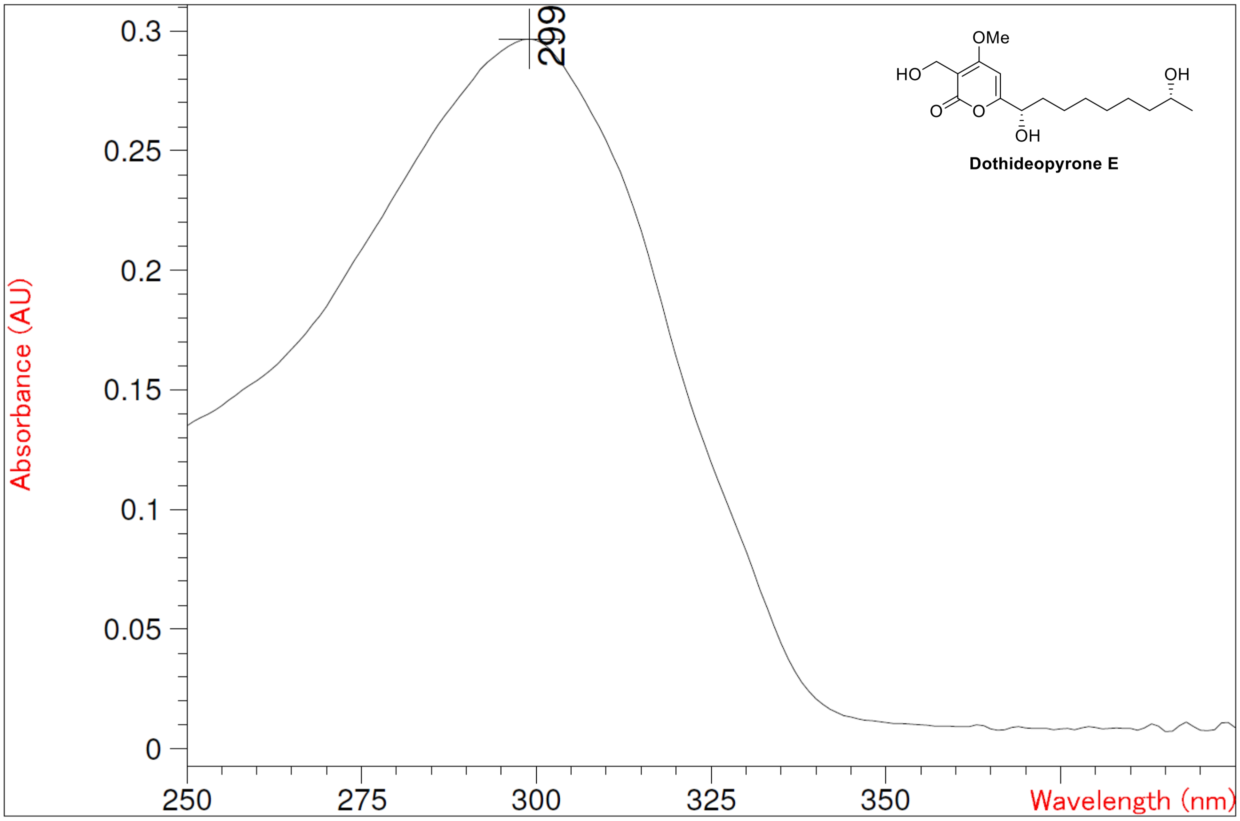


**Figure S-48** UV-Vis of synthetic dothideopyrone E (**1**) in MeOH.

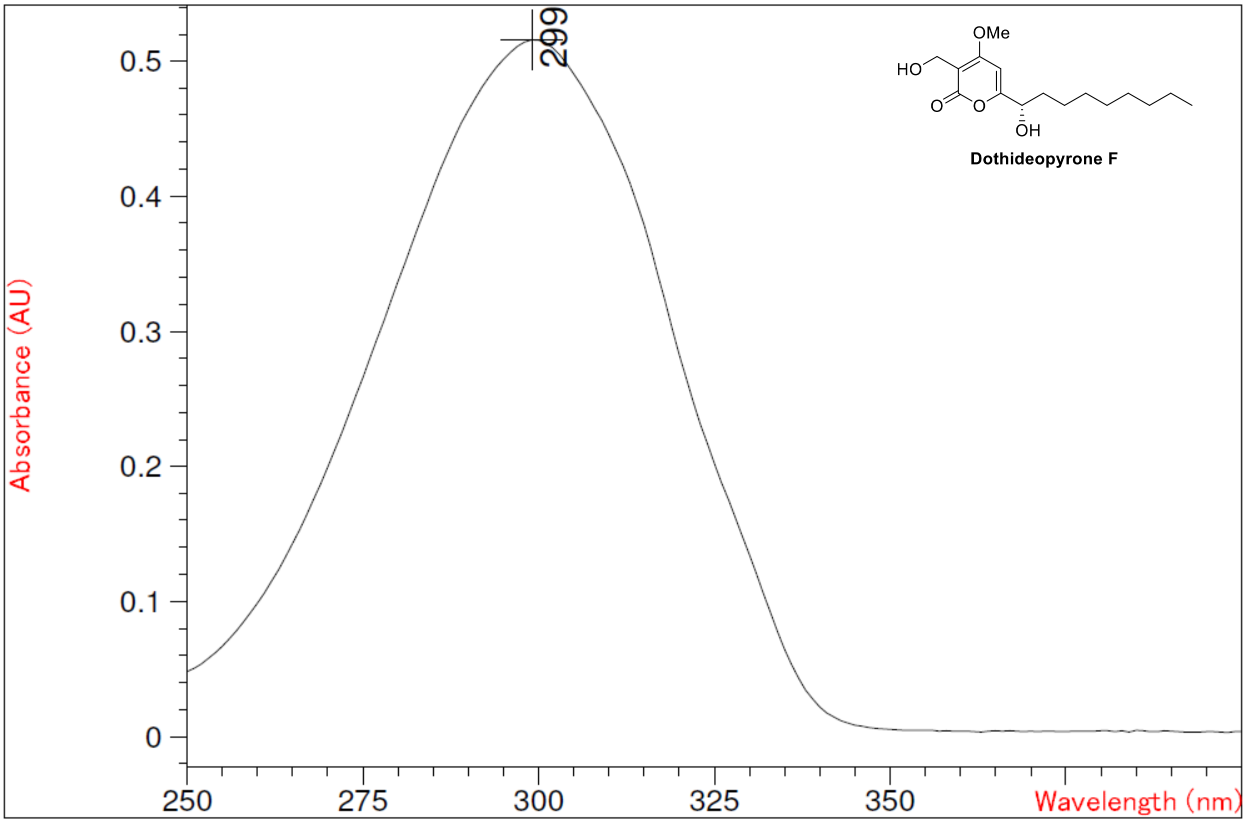


**Figure S-49** UV-Vis of synthetic dothideopyrone F (**2**) in MeOH.

Additional Info : Peak(s) manually integrated

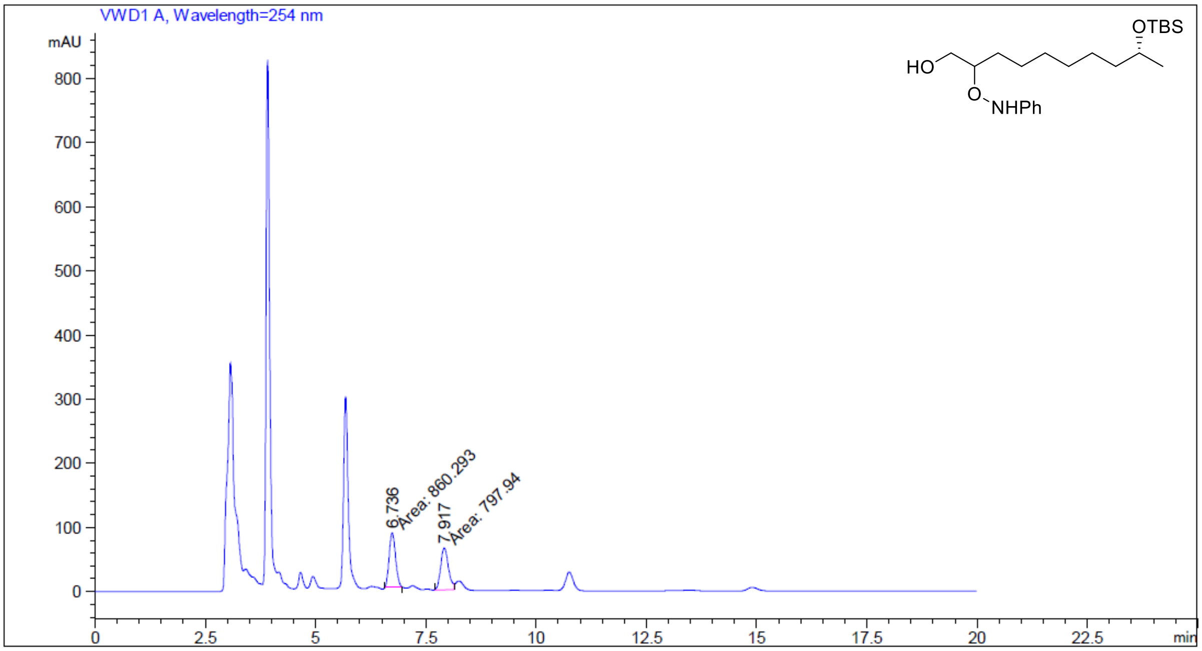


=====================================================================

Area Percent Report

=====================================================================

Sorted By : Signal
Multiplier : 1.0000
Dilution : 1.0000
Use Multiplier & Dilution Factor with ISTDs

Signal 1: VWD1 A, Wavelength=254 nm

| Peak # | RetTime [min] | Type | Width [min] | Area [mAU*s] | Height [mAU] | Area % |
|---|---|---|---|---|---|---|
| 1 | 6.736 | MM | 0.1678 | 860.29279 | 85.44933 | 51.8801 |
| 2 | 7.917 | MM | 0.2036 | 797.93988 | 65.31302 | 48.1199 |

Totals : 1658.23267 150.76234

**Figure S-50** HPLC chromatogram of 1:1 diastereomeric **S13** (AD-H, 2% *i*PrOH in hexane, 1 mL/min).

Additional Info : Peak(s) manually integrated

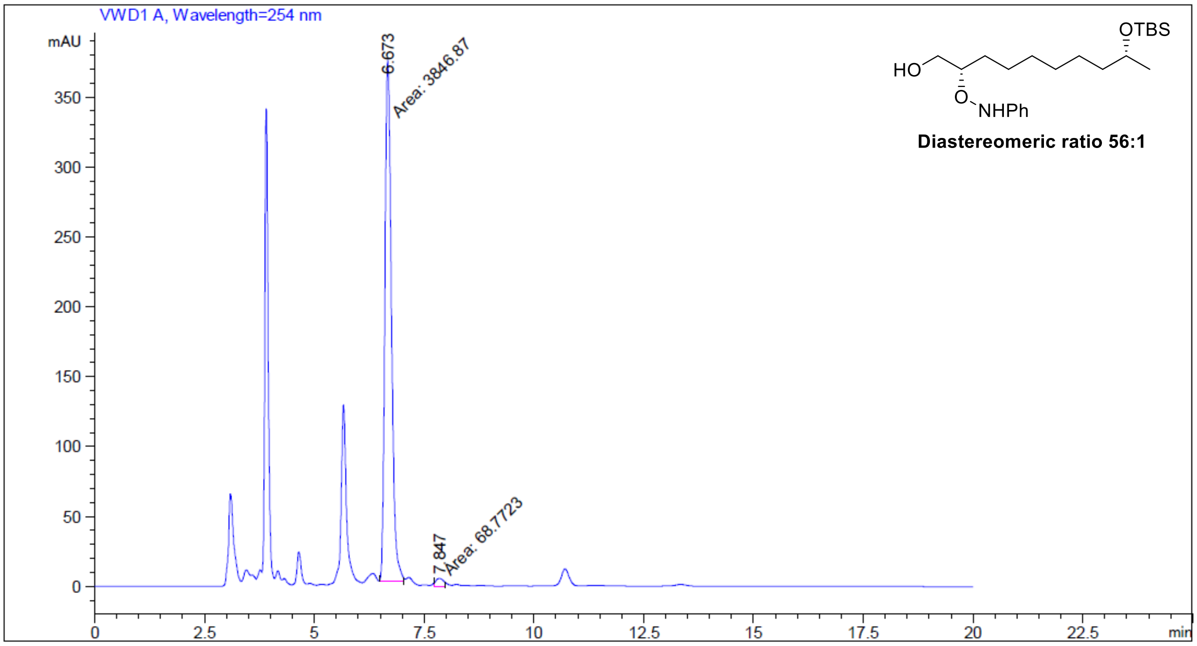


==================================================================

Area Percent Report

==================================================================

Sorted By : Signal
Multiplier : 1.0000
Dilution : 1.0000
Use Multiplier & Dilution Factor with ISTDs

Signal 1: VWD1 A, Wavelength=254 nm

| Peak # | RetTime [min] | Type | Width [min] | Area [mAU*s] | Height [mAU] | Area % |
|---|---|---|---|---|---|---|
| 1 | 6.673 | MM | 0.1717 | 3846.87256 | 373.36621 | 98.2437 |
| 2 | 7.847 | MM | 0.2036 | 68.77229 | 5.63063 | 1.7563 |

Totals : 3915.64485 378.99684

**Figure S-51** HPLC chromatogram of enantioenriched **S13** (AD-H, 2% *i*PrOH in hexane, 1 mL/min).

Sample Info : 1 ml/min, AD-H column, 5% iPrOH i heksan, UV-254 nm

Additional Info : Peak(s) manually integrated

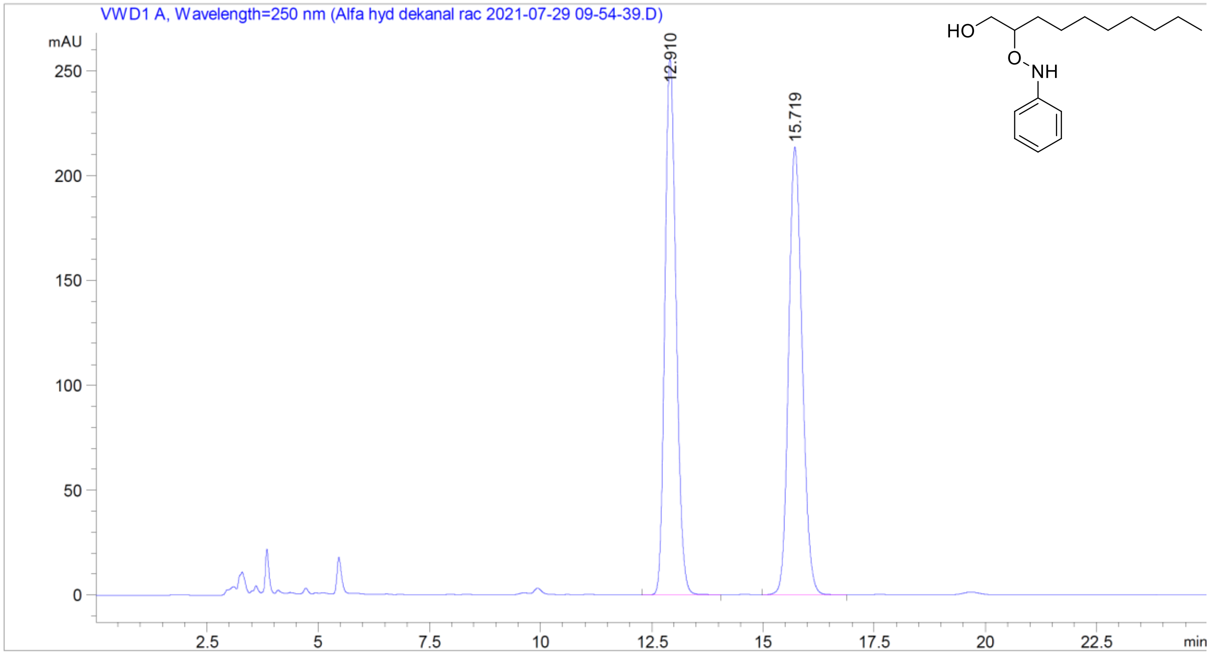


=====================================================================

Area Percent Report

=====================================================================

Sorted By : Signal
Multiplier : 1.0000
Dilution : 1.0000
Use Multiplier & Dilution Factor with ISTDs

Signal 1: VWD1 A, Wavelength=250 nm

| Peak # | RetTime [min] | Type | Width [min] | Area [mAU*s] | Height [mAU] | Area % |
|---|---|---|---|---|---|---|
| 1 | 12.910 | BB | 0.2723 | 4471.89795 | 255.31281 | 49.7230 |
| 2 | 15.719 | BB | 0.3291 | 4521.72559 | 213.45422 | 50.2770 |

Totals : 8993.62354 468.76703

=====================================================================

**Figure S-52** HPLC chromatogram of racemate **26b** (AD-H, 5% *i*PrOH in hexane, 1 mL/min).

Sample Info      : 1 ml/min, AD-H column, 5% iPrOH i heksan, UV-254 nm

Additional Info : Peak(s) manually integrated

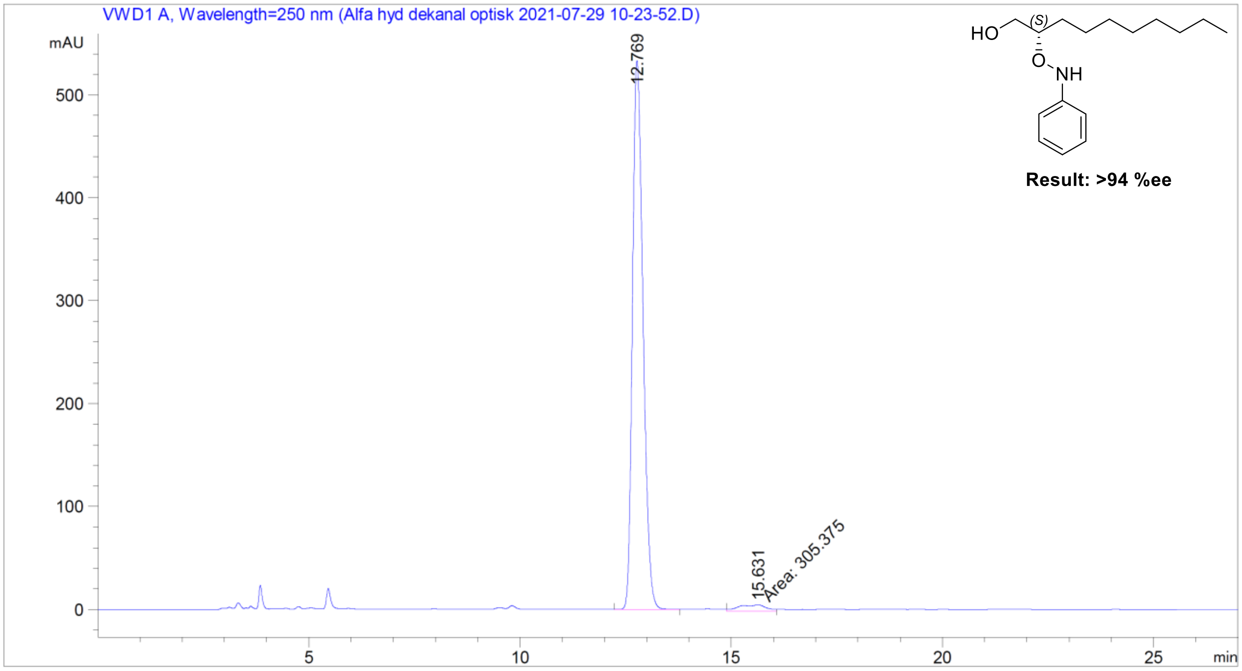


=====================================================================

Area Percent Report

=====================================================================

Sorted By : Signal

Multiplier : 1.0000

Dilution : 1.0000

Use Multiplier & Dilution Factor with ISTDs

Signal 1: VWD1 A, Wavelength=250 nm

| Peak # | RetTime [min] | Type | Width [min] | Area [mAU*s] | Height [mAU] | Area % |
|---|---|---|---|---|---|---|
| 1 | 12.769 | BB | 0.2705 | 9246.89063 | 532.84497 | 96.8031 |
| 2 | 15.631 | MM | 0.7922 | 305.37540 | 6.42502 | 3.1969 |
| Totals : | | | | 9552.26602 | 539.26999 | |

=====================================================================

**Figure S-53** HPLC chromatogram of optically active **26b** (AD-H, 5% *i*PrOH in hexane, 1 mL/min).

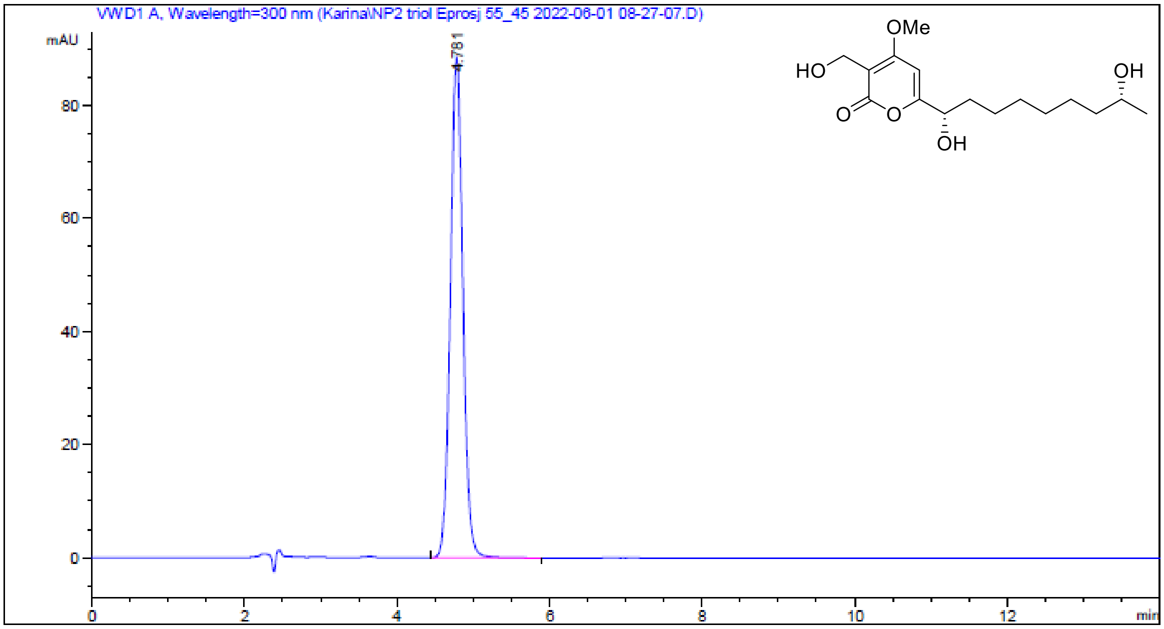


```
=====================================================================
                         Area Percent Report
=====================================================================

Sorted By             :      Signal
Multiplier            :      1.0000
Dilution              :      1.0000
Use Multiplier & Dilution Factor with ISTDs


Signal 1: VWD1 A, Wavelength=300 nm

Peak RetTime Type  Width     Area      Height     Area
  #   [min]        [min]   [mAU*s]     [mAU]        %
----|-------|----|-------|----------|----------|--------|
   1   4.781 BB    0.1748 1007.14160   88.48376 100.0000
```

**Figure S-54** HPLC chromatogram of dothideopyrone E (**1**).

## Chromatogram and Results

### Injection Details

| | | | |
|---|---|---|---|
| *Injection Name:* | **20220601_Marius_triol gradient 10-75%** | *Run Time (min):* | **9,00** |
| *Vial Number:* | **BB1** | *Injection Volume:* | **1,00** |
| *Injection Type:* | **Unknown** | *Channel:* | **UV_VIS_1** |
| *Calibration Level:* | | *Wavelength:* | **300** |
| *Instrument Method:* | **Gradient** | *Bandwidth:* | **n.a.** |
| *Processing Method:* | **Area** | *Dilution Factor:* | **1,0000** |
| *Injection Date/Time:* | **02.jun.22 15:01** | *Sample Weight:* | **1,0000** |

### Chromatogram

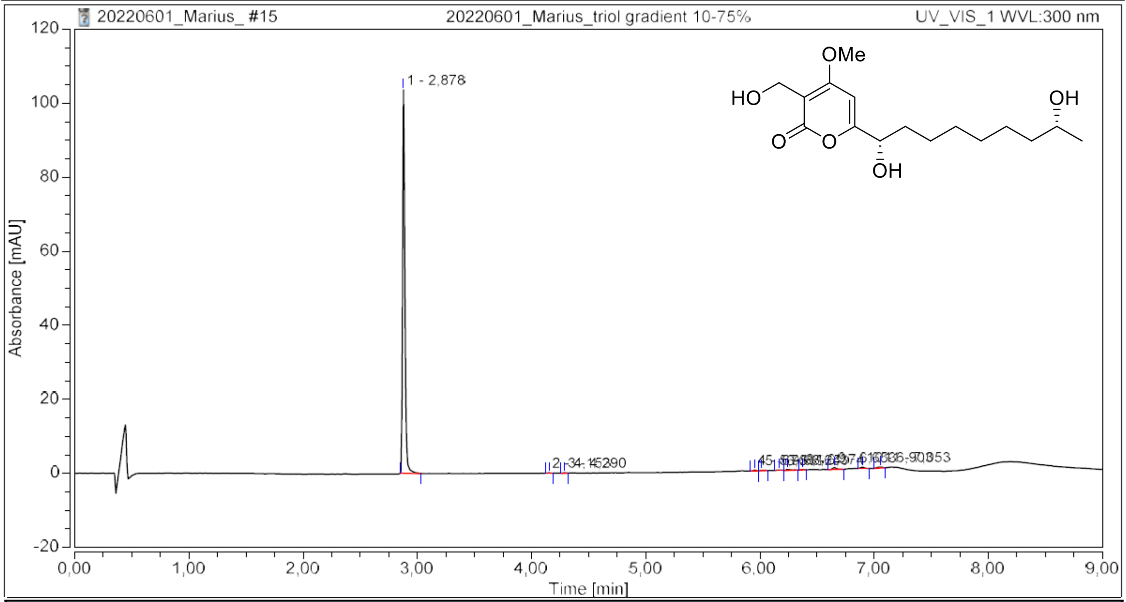


### Integration Results

| No. | Peak Name | Retention Time min | Area mAU*min | Height mAU | Relative Area % | Relative Height % | Amount n.a. |
|---|---|---|---|---|---|---|---|
| 1 | | 2,878 | 2,449 | 103,606 | 96,11 | 97,66 | n.a. |
| 2 | | 4,153 | 0,005 | 0,169 | 0,18 | 0,16 | n.a. |
| 3 | | 4,290 | 0,004 | 0,142 | 0,14 | 0,13 | n.a. |
| 4 | | 5,953 | 0,007 | 0,231 | 0,27 | 0,22 | n.a. |
| 5 | | 6,015 | 0,007 | 0,158 | 0,26 | 0,15 | n.a. |
| 6 | | 6,165 | 0,006 | 0,184 | 0,25 | 0,17 | n.a. |
| 7 | | 6,240 | 0,012 | 0,217 | 0,48 | 0,20 | n.a. |
| 8 | | 6,374 | 0,005 | 0,170 | 0,19 | 0,16 | n.a. |
| 9 | | 6,653 | 0,023 | 0,538 | 0,91 | 0,51 | n.a. |
| 10 | | 6,903 | 0,015 | 0,367 | 0,58 | 0,35 | n.a. |
| 11 | | 7,053 | 0,016 | 0,302 | 0,63 | 0,29 | n.a. |
| **Total:** | | | **2,548** | **106,085** | **100,00** | **100,00** | |

**Figure S-55** HPLC chromatogram of dothideopyrone E (**1**).

**Acquisition Parameter**

| | | | | | |
|---|---|---|---|---|---|
| Source Type | ESI | Set Capillary | 3500 V | Set Nebulizer | 0.3 Bar |
| Focus | Not active | Set End Plate Offset | -500 V | Set Dry Heater | 200 °C |
| Scan Begin | 50 m/z | Set Charging Voltage | 2000 V | Set Dry Gas | 4.0 l/min |
| Scan End | 1500 m/z | Set Corona | 0 nA | Set Divert Valve | Waste |
| | | | | Set APCI Heater | 0 °C |

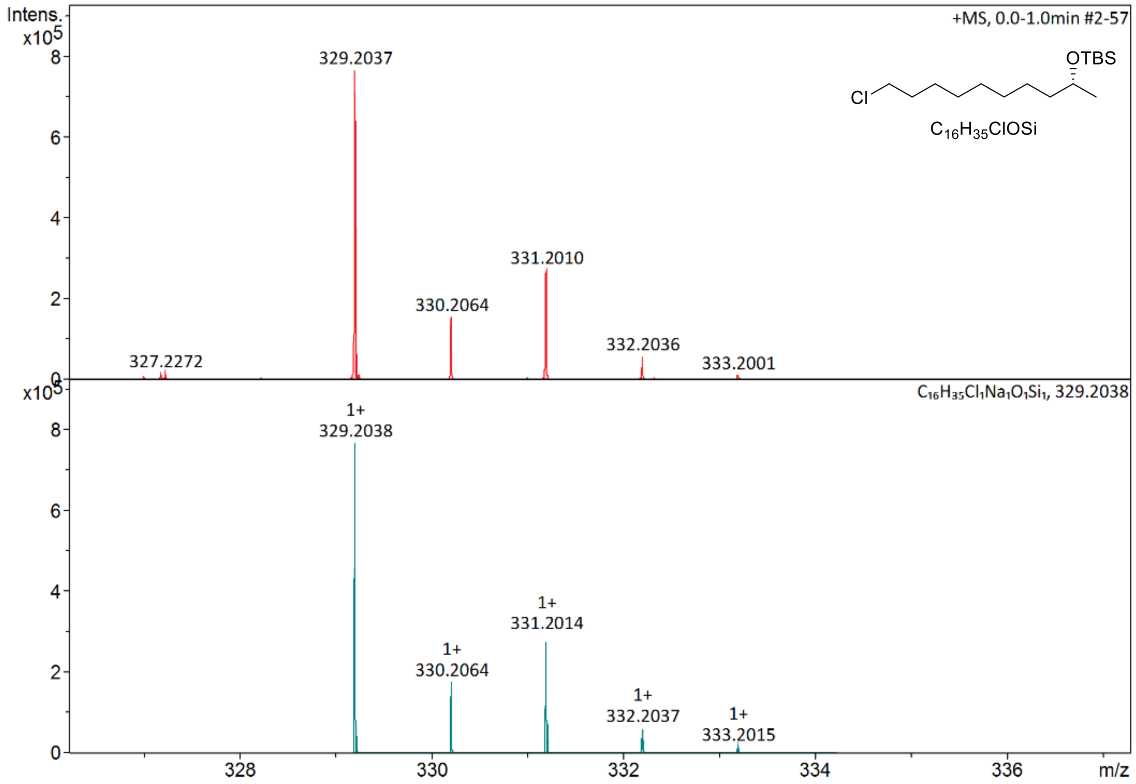


| Meas. m/z | Ion Formula | m/z | err [ppm] |
|---|---|---|---|
| 329.2037 | C16H35ClNaOSi | 329.2038 | 0.2 |

**Figure S-56** HRMS spectrum of compound **11**.

**Acquisition Parameter**

| | | | | | |
|---|---|---|---|---|---|
| Source Type | ESI | Set Capillary | 3500 V | Set Nebulizer | 0.5 Bar |
| Focus | Not active | Set End Plate Offset | -500 V | Set Dry Heater | 200 °C |
| Scan Begin | 50 m/z | Set Charging Voltage | 2000 V | Set Dry Gas | 4.0 l/min |
| Scan End | 1500 m/z | Set Corona | 0 nA | Set Divert Valve | Waste |
| | | | | Set APCI Heater | 0 °C |

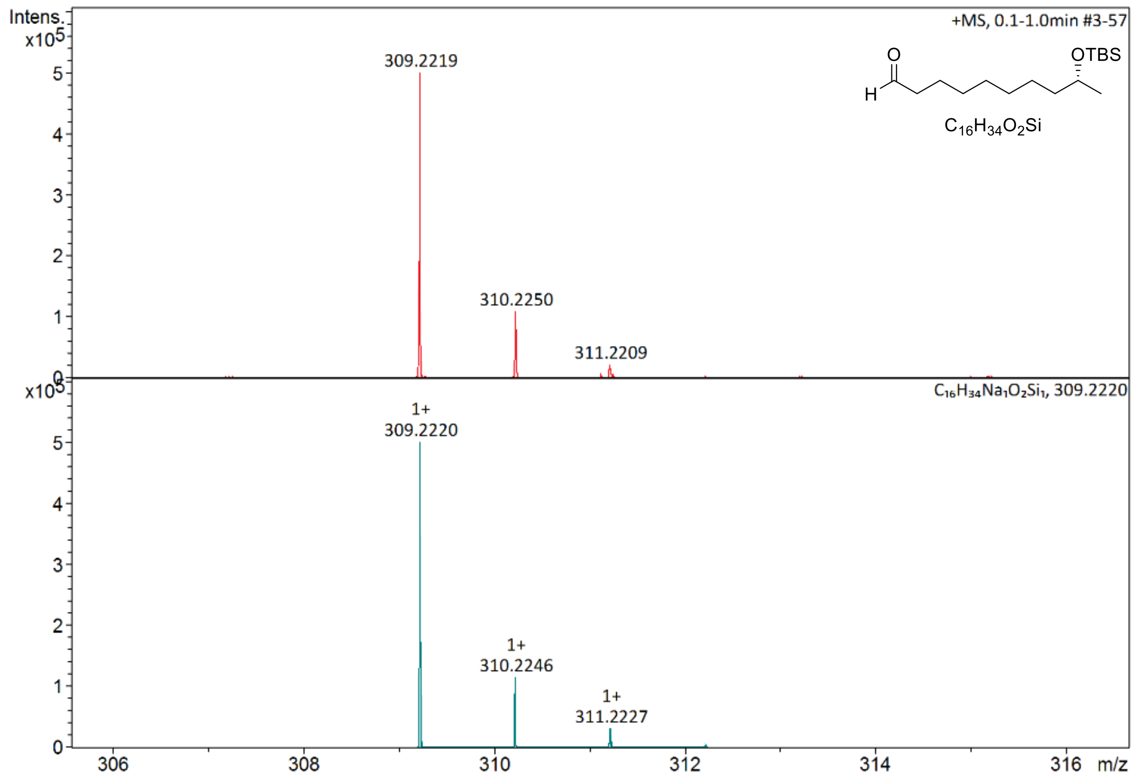


| Meas. m/z | Ion Formula | m/z | err [ppm] |
|---|---|---|---|
| 309.2219 | C14H29N6Si | 309.2217 | -0.5 |
| | C16H34NaO2Si | 309.2220 | 0.4 |

**Figure S-57** HRMS spectrum of compound **12**.

**Acquisition Parameter**

| | | | | | |
|---|---|---|---|---|---|
| Source Type | ESI | Set Capillary | 3500 V | Set Nebulizer | 0.5 Bar |
| Focus | Not active | Set End Plate Offset | -500 V | Set Dry Heater | 200 °C |
| Scan Begin | 50 m/z | Set Charging Voltage | 2000 V | Set Dry Gas | 4.0 l/min |
| Scan End | 1500 m/z | Set Corona | 0 nA | Set Divert Valve | Waste |
| | | | | Set APCI Heater | 0 °C |

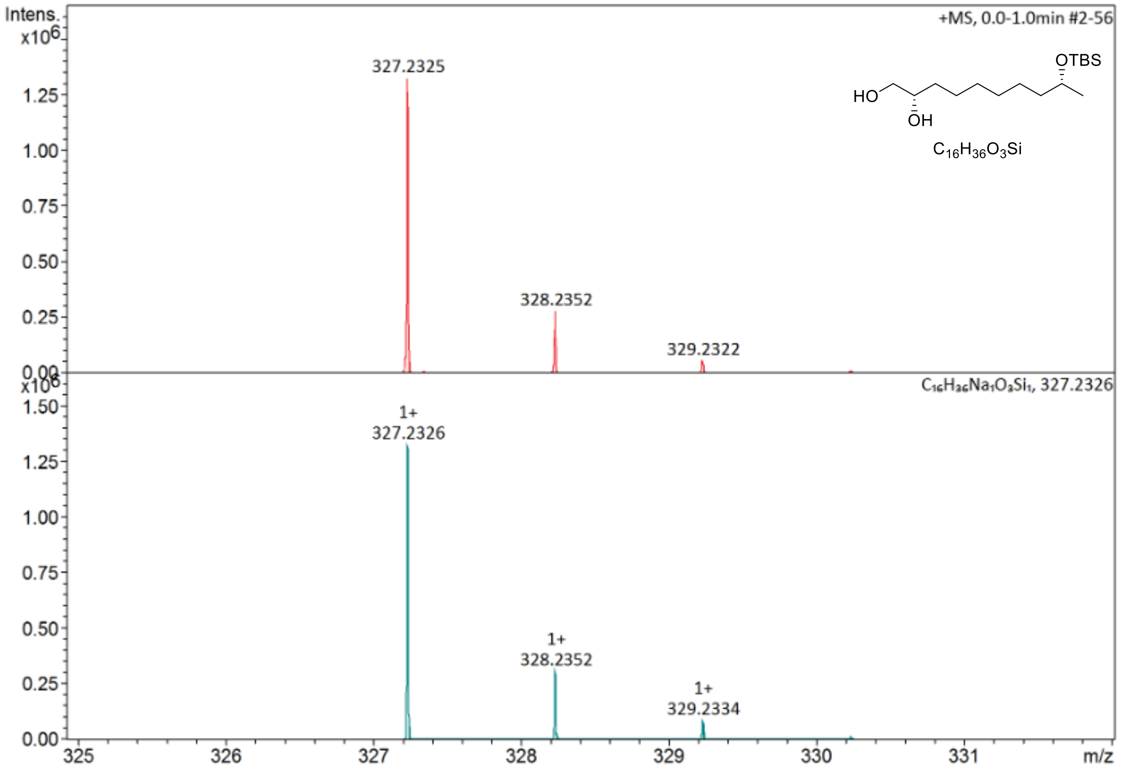


| Meas. m/z | Ion Formula | m/z | err [ppm] |
|---|---|---|---|
| 327.2325 | C16H36NaO3Si | 327.2326 | 0.2 |

**Figure S-58** HRMS spectrum of compound **13**.

**Acquisition Parameter**

| | | | | | |
|---|---|---|---|---|---|
| Source Type | ESI | Set Capillary | 3500 V | Set Nebulizer | 0.3 Bar |
| Focus | Not active | Set End Plate Offset | -500 V | Set Dry Heater | 200 °C |
| Scan Begin | 50 m/z | Set Charging Voltage | 2000 V | Set Dry Gas | 4.0 l/min |
| Scan End | 1500 m/z | Set Corona | 0 nA | Set Divert Valve | Waste |
| | | | | Set APCI Heater | 0 °C |

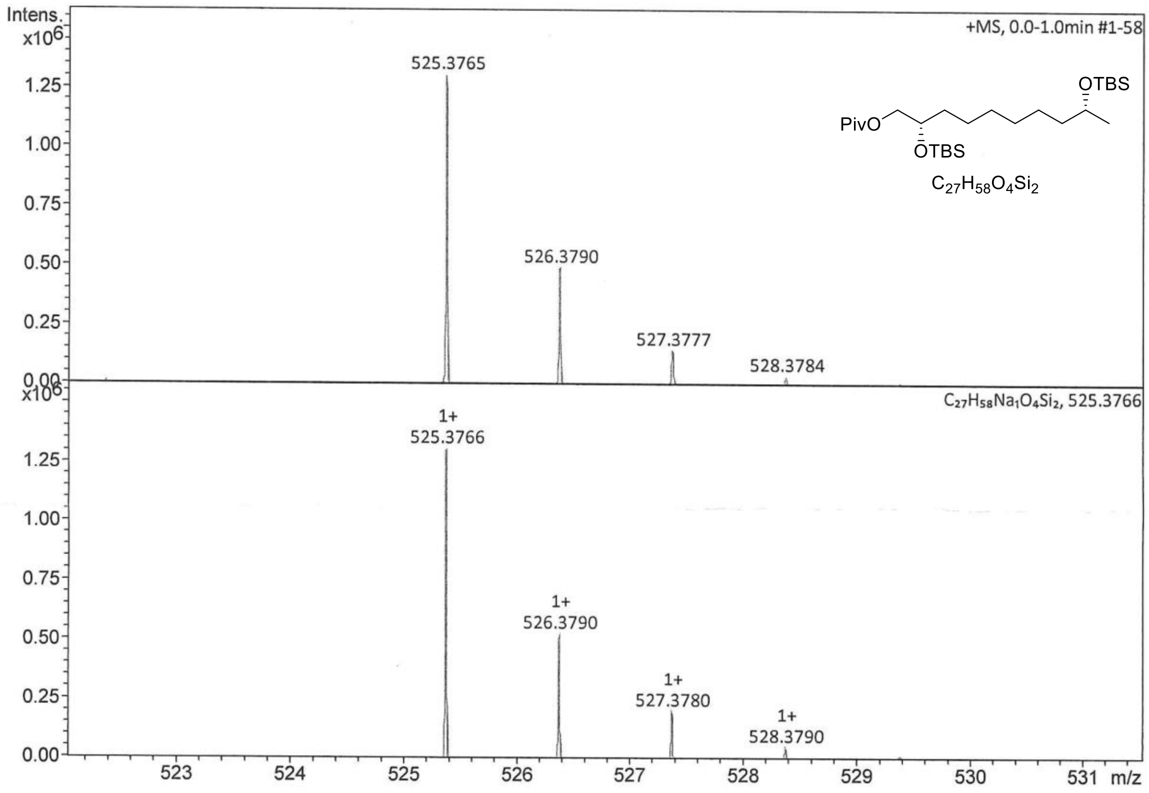


| Meas. m/z | Ion Formula | m/z | err [ppm] |
|---|---|---|---|
| 525.3765 | C33H53O3Si | 525.3758 | -1.3 |
| | C27H45N10O | 525.3772 | 1.3 |
| | C27H58NaO4Si2 | 525.3766 | 0.1 |
| | C29H50N4NaO3 | 525.3775 | 1.8 |
| | C28H54NaO7 | 525.3762 | -0.7 |

**Figure S-59** HRMS spectrum of compound **14**.

## Acquisition Parameter

| | | | | | |
|---|---|---|---|---|---|
| Source Type | ESI | Set Capillary | 3500 V | Set Nebulizer | 0.3 Bar |
| Focus | Not active | Set End Plate Offset | -500 V | Set Dry Heater | 200 °C |
| Scan Begin | 50 m/z | Set Charging Voltage | 2000 V | Set Dry Gas | 4.0 l/min |
| Scan End | 1500 m/z | Set Corona | 0 nA | Set Divert Valve | Waste |
| | | | | Set APCI Heater | 0 °C |

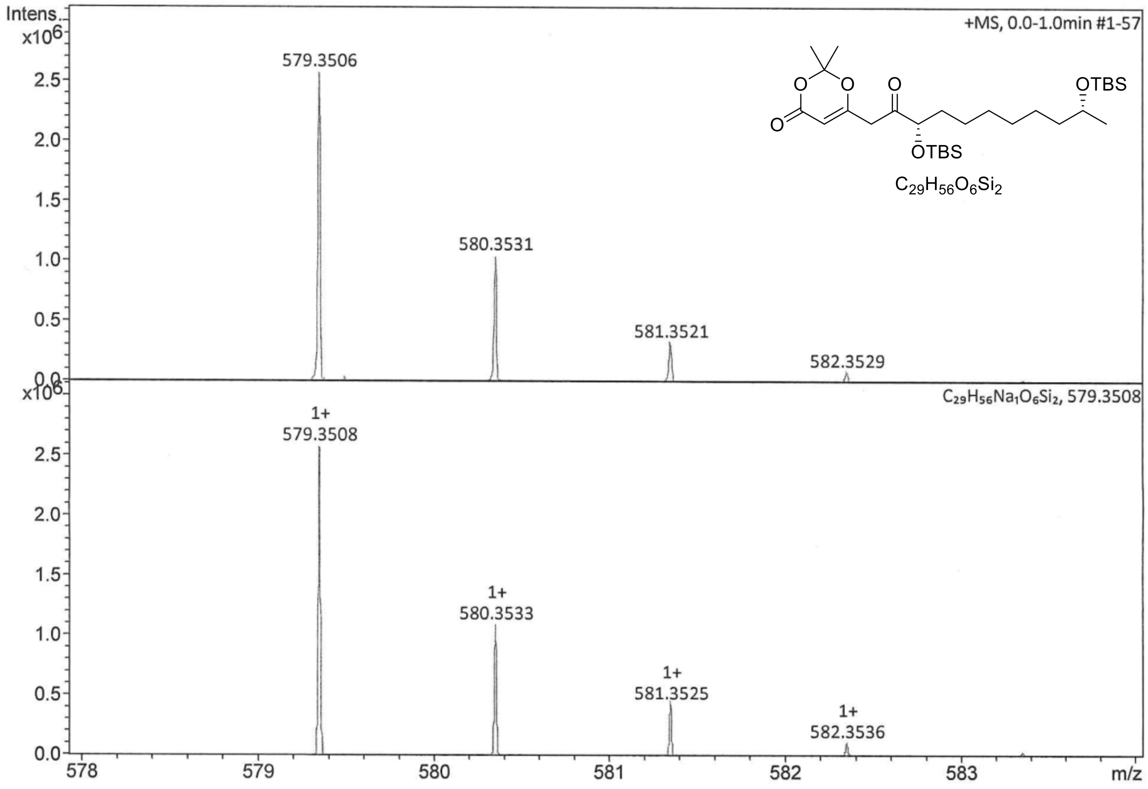


| Meas. m/z | Ion Formula | m/z | err [ppm] |
|---|---|---|---|
| 242.2841 | C16H36N | 242.2842 | 0.5 |
| 521.3087 | C32H45O4Si | 521.3082 | -1.1 |
| | C33H41N4Si | 521.3095 | 1.4 |
| | C26H37N10O2 | 521.3095 | 1.5 |
| | C25H41N6O6 | 521.3082 | -1.0 |
| | C26H50NaO5Si2 | 521.3089 | 0.3 |
| | C27H46NaO8 | 521.3085 | -0.5 |
| 579.3506 | C35H51O5Si | 579.3500 | -1.0 |
| | C36H47N4OSi | 579.3514 | 1.3 |
| | C29H43N10O3 | 579.3514 | 1.4 |
| | C29H56NaO6Si2 | 579.3508 | 0.3 |
| | C31H48N4NaO5 | 579.3517 | 1.9 |
| | C30H52NaO9 | 579.3504 | -0.4 |
| 1135.7124 | C56H107N6O10Si4 | 1135.7120 | -0.4 |
| | C57H103N10O6Si4 | 1135.7134 | 0.8 |
| | C55H104N10NaO6Si4 | 1135.7110 | -1.3 |
| | C58H112NaO12Si4 | 1135.7123 | -0.1 |
| | C56H100N14NaO2Si4 | 1135.7123 | -0.1 |
| | C59H108N4NaO8Si4 | 1135.7136 | 1.1 |

**Figure S-60** HRMS spectrum of compound **19**.

**Acquisition Parameter**

| | | | | | |
|---|---|---|---|---|---|
| Source Type | ESI | Set Capillary | 3500 V | Set Nebulizer | 0.3 Bar |
| Focus | Not active | Set End Plate Offset | -500 V | Set Dry Heater | 200 °C |
| Scan Begin | 50 m/z | Set Charging Voltage | 2000 V | Set Dry Gas | 4.0 l/min |
| Scan End | 1500 m/z | Set Corona | 0 nA | Set Divert Valve | Waste |
| | | | | Set APCI Heater | 0 °C |

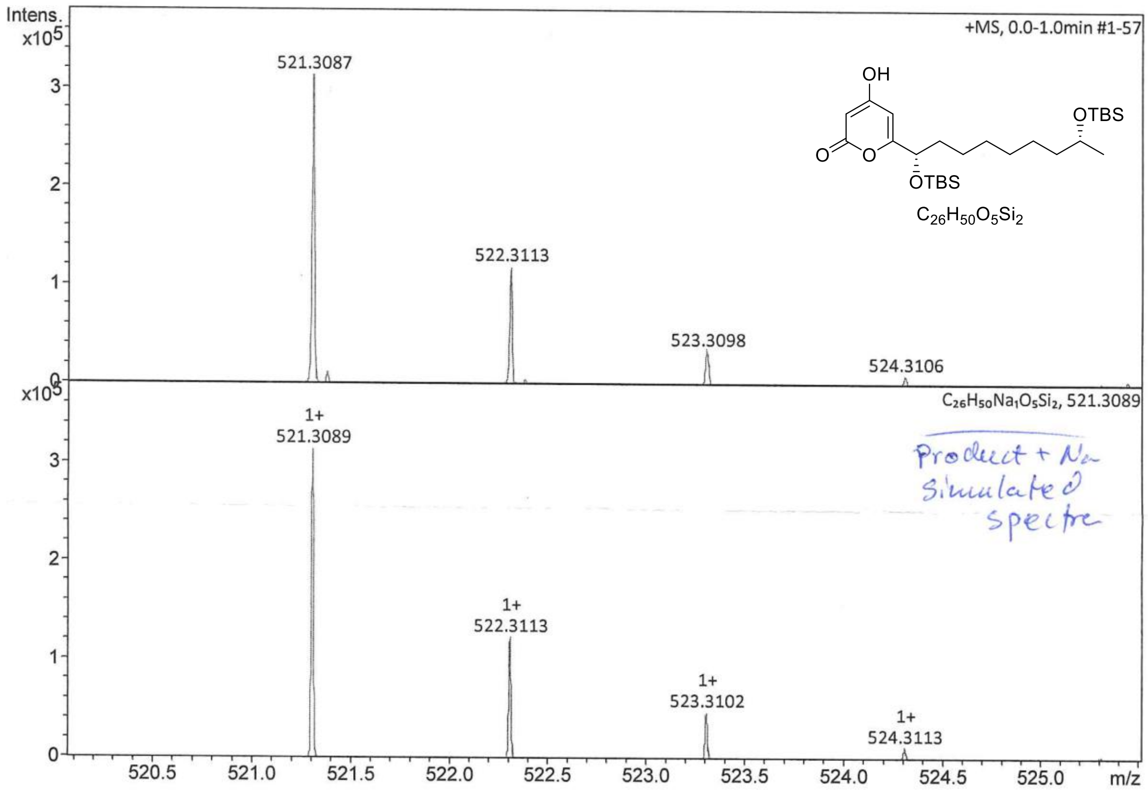


| Meas. m/z | Ion Formula | m/z | err [ppm] |
|---|---|---|---|
| 408.3084 | C20H38N7O2 | 408.3081 | -0.6 |
| | C27H42NSi | 408.3081 | -0.7 |
| | C22H43NNaO4 | 408.3084 | 0.1 |
| 521.3087 | C24H45N6O3Si2 | 521.3086 | -0.2 |
| | C25H41N6O6 | 521.3082 | -1.0 |
| | C17H41N12O5Si | 521.3087 | -0.2 |
| | C26H50NaO5Si2 | 521.3089 | 0.3 |
| | C27H46NaO8 | 521.3085 | -0.5 |
| | C19H46N6NaO7Si | 521.3089 | 0.4 |
| 1001.6215 | C60H86N2NaO9 | 1001.6226 | 1.1 |

**Figure S-61** HRMS spectrum of compound **20**.

## Acquisition Parameter

| | | | | | |
|---|---|---|---|---|---|
| Source Type | ESI | Set Capillary | 3500 V | Set Nebulizer | 0.3 Bar |
| Focus | Not active | Set End Plate Offset | -500 V | Set Dry Heater | 200 °C |
| Scan Begin | 50 m/z | Set Charging Voltage | 2000 V | Set Dry Gas | 4.0 l/min |
| Scan End | 1500 m/z | Set Corona | 0 nA | Set Divert Valve | Waste |
| | | | | Set APCI Heater | 0 °C |

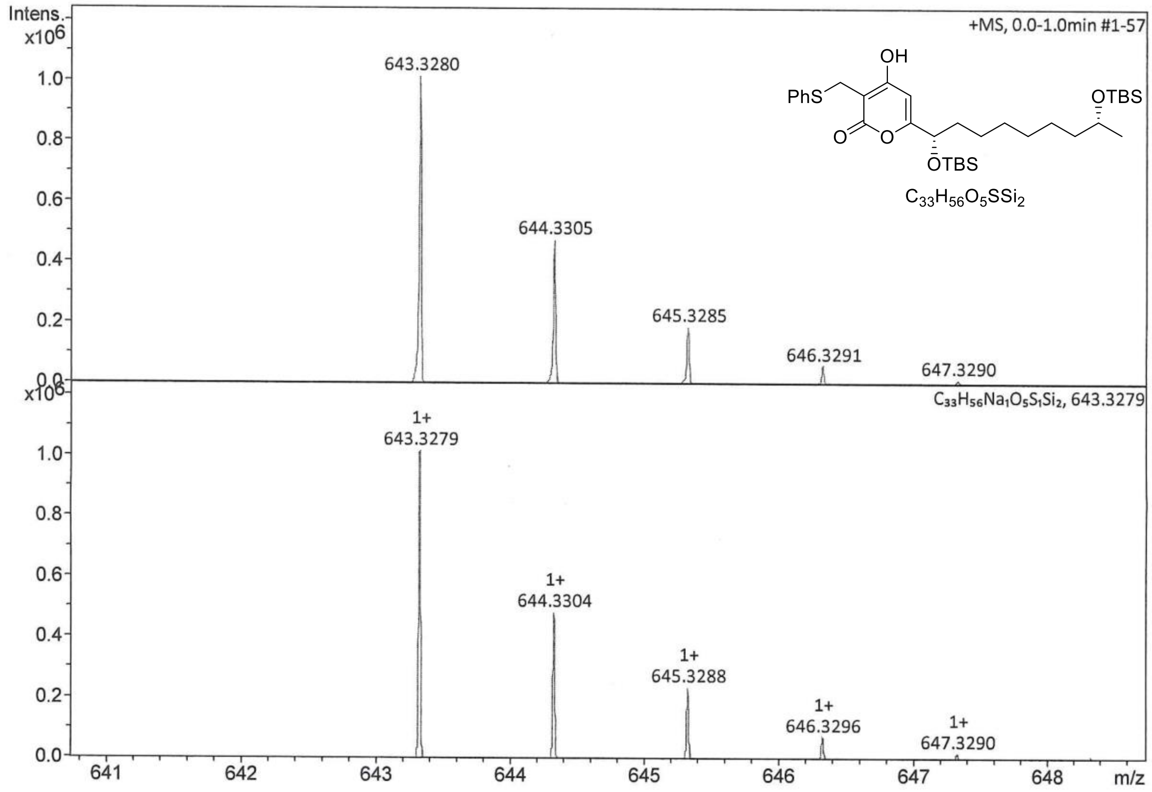


| Meas. m/z | Ion Formula | m/z | err [ppm] |
|---|---|---|---|
| 643.3280 | C39H51O4SSi | 643.3272 | -1.2 |
| | C31H51N6O3SSi2 | 643.3276 | -0.5 |
| | C40H47N4SSi | 643.3285 | 0.9 |
| | C38H51O5Si2 | 643.3270 | -1.6 |
| | C32H43N10O3Si | 643.3283 | 0.6 |
| | C39H47N4OSi2 | 643.3283 | 0.5 |
| | C31H47N6O7Si | 643.3270 | -1.5 |
| | C41H39N8 | 643.3292 | 2.0 |
| | C33H43N10O2S | 643.3286 | 1.0 |
| | C40H43N4O4 | 643.3279 | -0.1 |
| | C32H47N6O6S | 643.3272 | -1.1 |
| | C33H56NaO5SSi2 | 643.3279 | -0.1 |
| | C34H52N4NaOSSi2 | 643.3293 | 2.0 |
| | C34H48N4NaO5Si | 643.3286 | 1.0 |
| | C33H52NaO9Si | 643.3273 | -1.1 |
| | C35H48N4NaO4S | 643.3288 | 1.4 |
| | C39H40N8Na | 643.3268 | -1.8 |
| | C34H52NaO8S | 643.3275 | -0.7 |

**Figure S-62** HRMS spectrum of compound **21**.

## Acquisition Parameter

| | | | | | |
|---|---|---|---|---|---|
| Source Type | ESI | Set Capillary | 3500 V | Set Nebulizer | 0.3 Bar |
| Focus | Not active | Set End Plate Offset | -500 V | Set Dry Heater | 200 °C |
| Scan Begin | 50 m/z | Set Charging Voltage | 2000 V | Set Dry Gas | 4.0 l/min |
| Scan End | 1500 m/z | Set Corona | 0 nA | Set Divert Valve | Waste |
| | | | | Set APCI Heater | 0 °C |

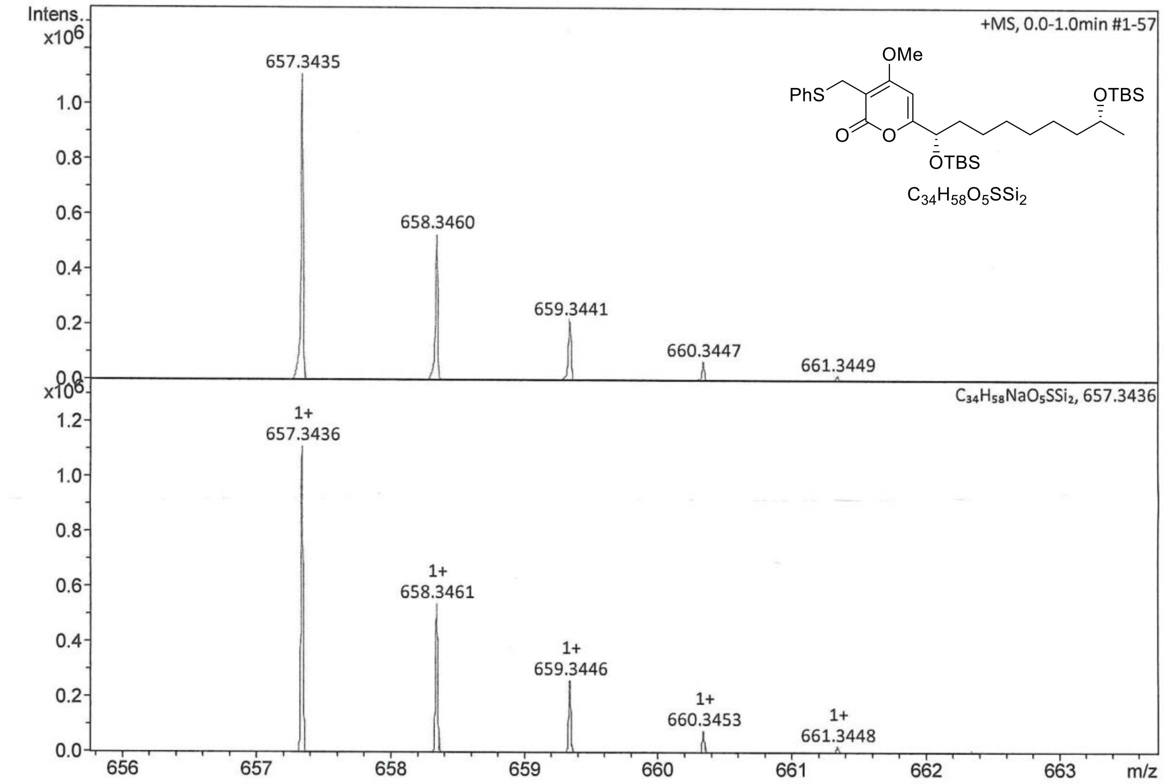


| Meas. m/z | Ion Formula | m/z | err [ppm] |
|---|---|---|---|
| 325.2169 | C14H29N6OSi | 325.2167 | -0.8 |
| | C15H29N6S | 325.2169 | -0.1 |
| | C16H34NaO3Si | 325.2169 | 0.0 |
| | C17H34NaO2S | 325.2172 | 0.7 |
| 657.3435 | C40H53O4SSi | 657.3428 | -1.0 |
| | C32H53N6O3SSi2 | 657.3433 | -0.3 |
| | C41H49N4SSi | 657.3442 | 1.1 |
| | C39H53O5Si2 | 657.3426 | -1.3 |
| | C33H45N10O3Si | 657.3440 | 0.8 |
| | C40H49N4OSi2 | 657.3439 | 0.7 |
| | C32H49N6O7Si | 657.3427 | -1.3 |
| | C34H45N10O2S | 657.3442 | 1.1 |
| | C41H45N4O4 | 657.3435 | 0.1 |
| | C33H49N6O6S | 657.3429 | -0.9 |
| | C34H58NaO5SSi2 | 657.3436 | 0.1 |
| | C35H50N4NaO5Si | 657.3443 | 1.2 |
| | C34H54NaO9Si | 657.3429 | -0.8 |
| | C36H50N4NaO4S | 657.3445 | 1.5 |
| | C35H54NaO8S | 657.3432 | -0.5 |

**Figure S-63** HRMS spectrum of compound **22**.

**Acquisition Parameter**

| | | | | | |
|---|---|---|---|---|---|
| Source Type | ESI | Set Capillary | 3500 V | Set Nebulizer | 0.3 Bar |
| Focus | Not active | Set End Plate Offset | -500 V | Set Dry Heater | 200 °C |
| Scan Begin | 50 m/z | Set Charging Voltage | 2000 V | Set Dry Gas | 4.0 l/min |
| Scan End | 1500 m/z | Set Corona | 0 nA | Set Divert Valve | Waste |
| | | | | Set APCI Heater | 0 °C |

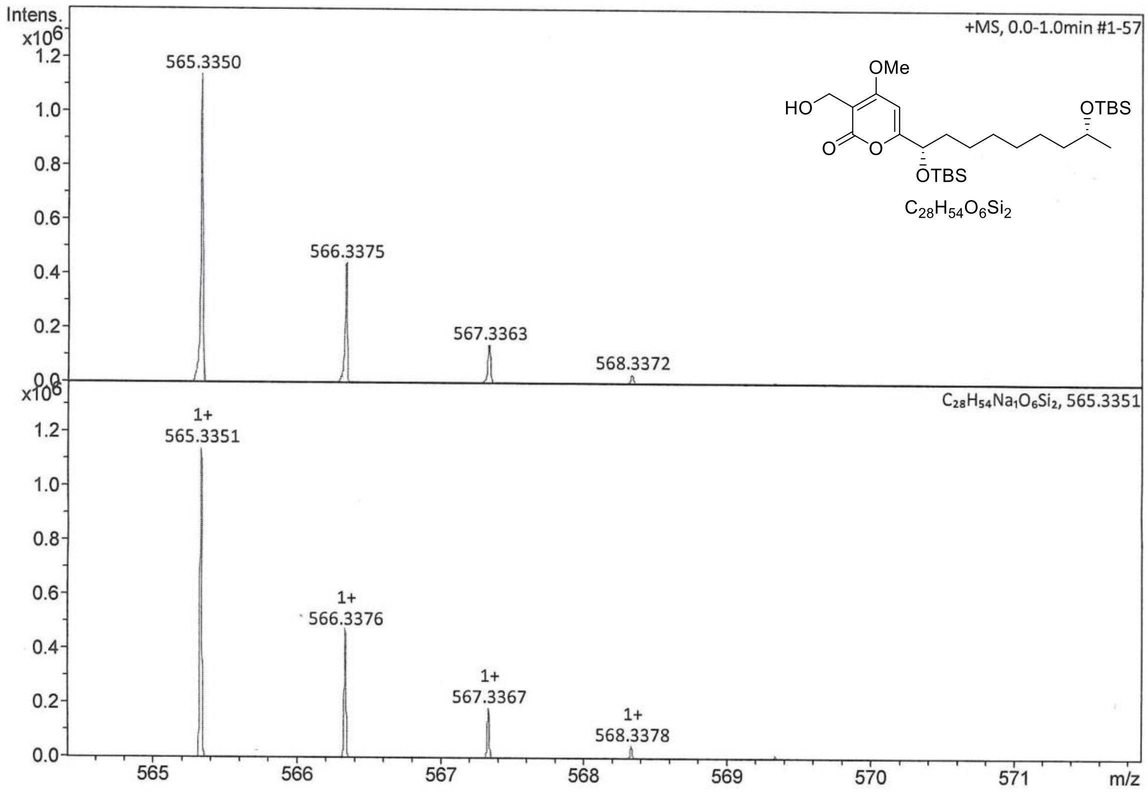


| Meas. m/z | Ion Formula | m/z | err [ppm] |
|---|---|---|---|
| 565.3350 | C26H49N6O4Si2 | 565.3348 | -0.3 |
| | C34H49O5Si | 565.3344 | -1.1 |
| | C35H45N4OSi | 565.3357 | 1.3 |
| | C28H41N10O3 | 565.3358 | 1.4 |
| | C19H45N12O6Si | 565.3349 | -0.2 |
| | C27H45N6O7 | 565.3344 | -1.0 |
| | C11H45N18O5Si2 | 565.3353 | 0.6 |
| | C28H54NaO6Si2 | 565.3351 | 0.2 |
| | C30H46N4NaO5 | 565.3360 | 1.9 |
| | C21H50N6NaO8Si | 565.3352 | 0.3 |
| | C29H50NaO9 | 565.3347 | -0.5 |
| | C18H42N16NaO2Si | 565.3338 | -2.1 |
| | C10H42N22NaOSi2 | 565.3343 | -1.3 |
| | C3H38N28NaO3Si | 565.3343 | -1.2 |

**Figure S-64** HRMS spectrum of compound **24**.

**Acquisition Parameter**

| | | | | | |
|---|---|---|---|---|---|
| Source Type | ESI | Set Capillary | 3500 V | Set Nebulizer | 0.3 Bar |
| Focus | Not active | Set End Plate Offset | -500 V | Set Dry Heater | 200 °C |
| Scan Begin | 50 m/z | Set Charging Voltage | 2000 V | Set Dry Gas | 4.0 l/min |
| Scan End | 1500 m/z | Set Corona | 0 nA | Set Divert Valve | Waste |
| | | | | Set APCI Heater | 0 °C |

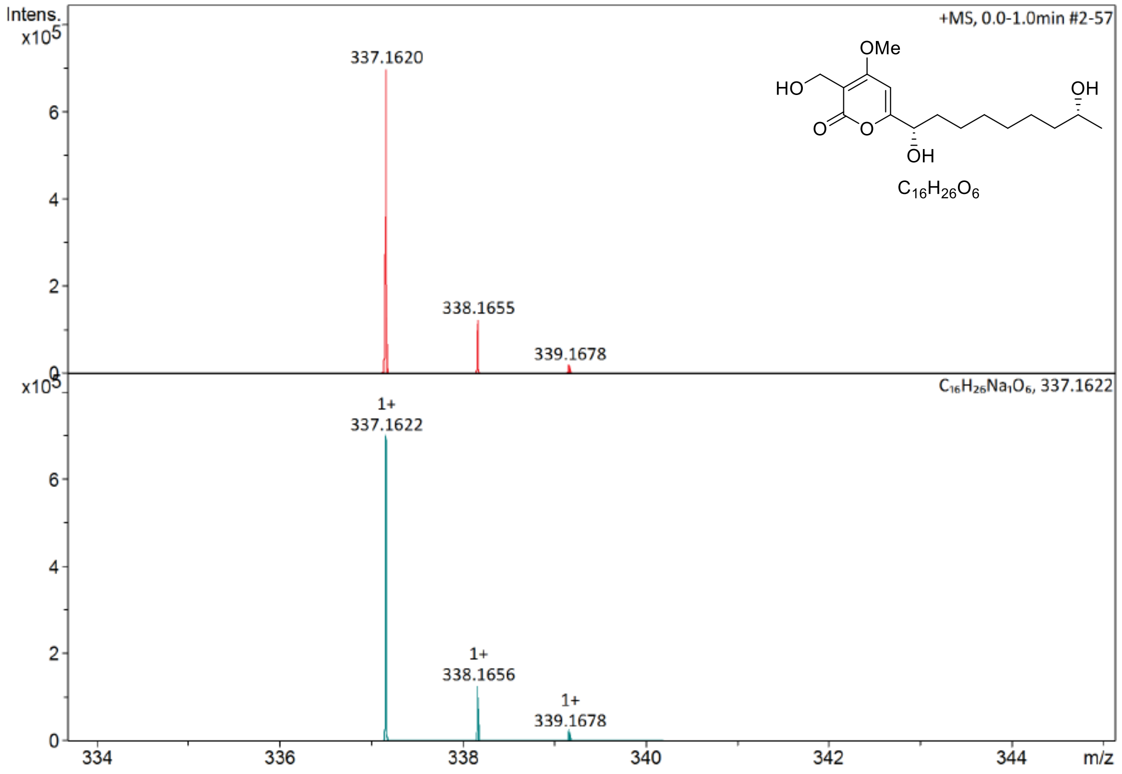


| Meas. m/z | Ion Formula | m/z | err [ppm] |
|---|---|---|---|
| 242.2841 | C16H36N | 242.2842 | 0.7 |
| 337.1620 | C14H21N6O4 | 337.1619 | -0.4 |
| | C16H26NaO6 | 337.1622 | 0.4 |

**Figure S-65** HRMS spectrum of dothideopyrone E (**1**).

**Acquisition Parameter**

| | | | | | |
|---|---|---|---|---|---|
| Source Type | ESI | Set Capillary | 3500 V | Set Nebulizer | 0.3 Bar |
| Focus | Not active | Set End Plate Offset | -500 V | Set Dry Heater | 200 °C |
| Scan Begin | 50 m/z | Set Charging Voltage | 2000 V | Set Dry Gas | 4.0 l/min |
| Scan End | 1500 m/z | Set Corona | 0 nA | Set Divert Valve | Waste |
| | | | | Set APCI Heater | 0 °C |

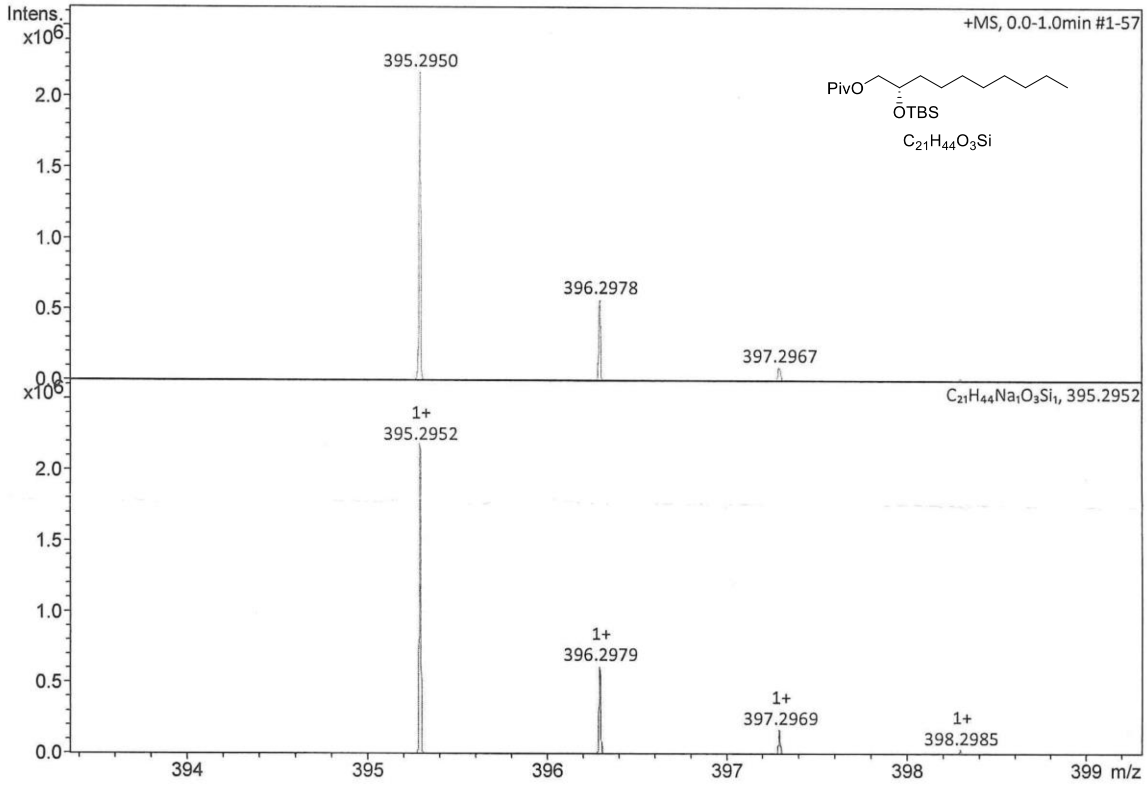


| Meas. m/z | Ion Formula | m/z | err [ppm] |
|---|---|---|---|
| 395.2950 | C18H43N2O5Si | 395.2936 | -3.5 |
| | C19H39N6OSi | 395.2949 | -0.1 |
| | C27H39O2 | 395.2945 | -1.3 |
| | C21H44NaO3Si | 395.2952 | 0.6 |

**Figure S-66** HRMS spectrum of compound **S2**.

**Acquisition Parameter**

| | | | | | |
|---|---|---|---|---|---|
| Source Type | ESI | Set Capillary | 3500 V | Set Nebulizer | 0.3 Bar |
| Focus | Not active | Set End Plate Offset | -500 V | Set Dry Heater | 200 °C |
| Scan Begin | 50 m/z | Set Charging Voltage | 2000 V | Set Dry Gas | 4.0 l/min |
| Scan End | 1500 m/z | Set Corona | 0 nA | Set Divert Valve | Waste |
| | | | | Set APCI Heater | 0 °C |

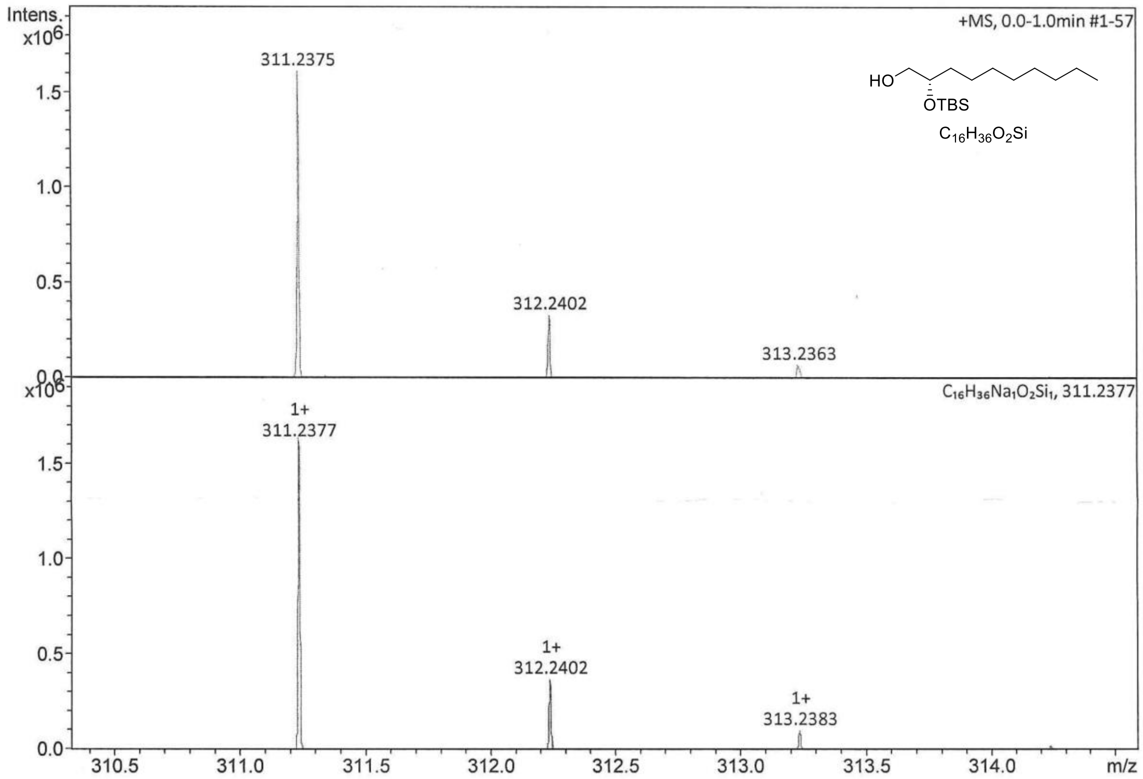


| Meas. m/z | Ion Formula | m/z | err [ppm] |
|---|---|---|---|
| 311.2375 | C14H31N6Si | 311.2374 | -0.4 |
| | C22H31O | 311.2369 | -1.9 |
| | C16H36NaO2Si | 311.2377 | 0.5 |

**Figure S-67** HRMS spectrum of compound **S3**.

**Acquisition Parameter**

| | | | | | |
|---|---|---|---|---|---|
| Source Type | ESI | Set Capillary | 3500 V | Set Nebulizer | 0.3 Bar |
| Focus | Not active | Set End Plate Offset | -500 V | Set Dry Heater | 200 °C |
| Scan Begin | 50 m/z | Set Charging Voltage | 2000 V | Set Dry Gas | 4.0 l/min |
| Scan End | 1500 m/z | Set Corona | 0 nA | Set Divert Valve | Waste |
| | | | | Set APCI Heater | 0 °C |

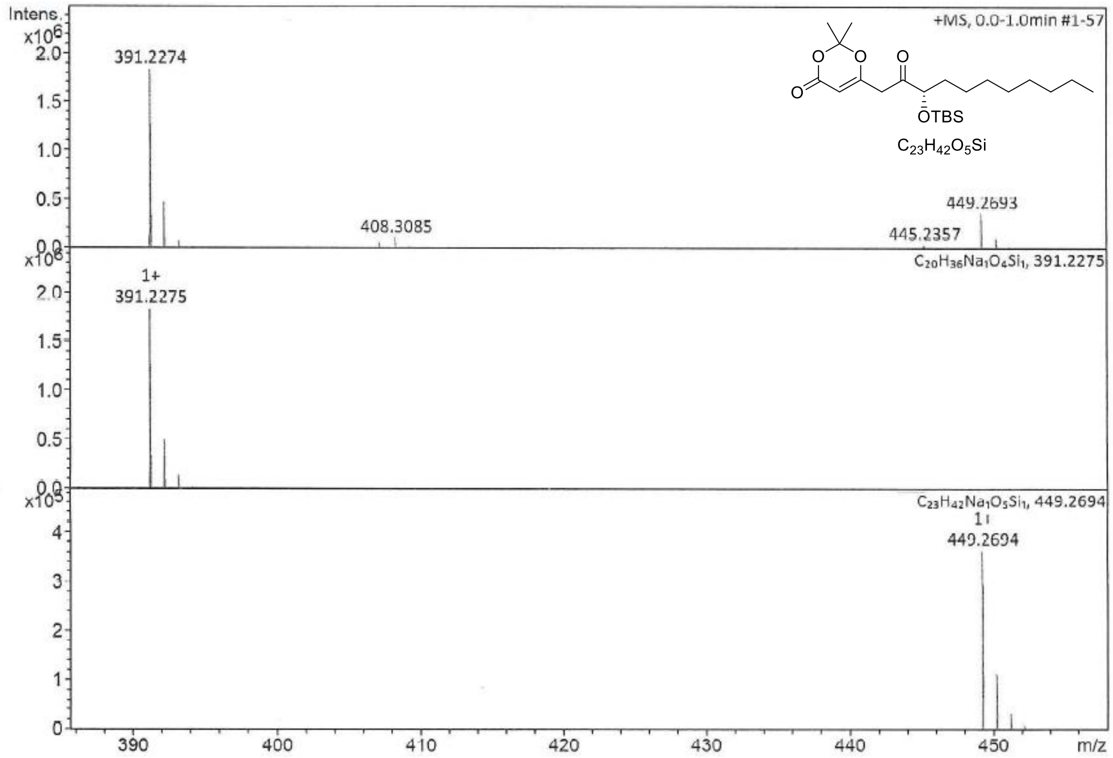


| Meas. m/z | Ion Formula | m/z | err [ppm] |
|---|---|---|---|
| 391.2274 | C18H31N6O2Si | 391.2272 | -0.4 |
| | C20H36NaO4Si | 391.2275 | 0.3 |
| 449.2693 | C29H37O4 | 449.2686 | -1.5 |
| | C21H37N6O3Si | 449.2691 | -0.5 |
| | C30H33N4 | 449.2700 | 1.4 |
| | C23H42NaO5Si | 449.2694 | 0.1 |

**Figure S-68** HRMS spectrum of compound **S6**.

**Acquisition Parameter**

| | | | | | |
|---|---|---|---|---|---|
| Source Type | ESI | Ion Polarity | Positive | Set Nebulizer | 0.4 Bar |
| Focus | Active | Set Capillary | 3500 V | Set Dry Heater | 200 °C |
| Scan Begin | 50 m/z | Set End Plate Offset | -500 V | Set Dry Gas | 4.0 l/min |
| Scan End | 2000 m/z | Set Charging Voltage | 2000 V | Set Divert Valve | Waste |
| | | Set Corona | 0 nA | Set APCI Heater | 0 °C |

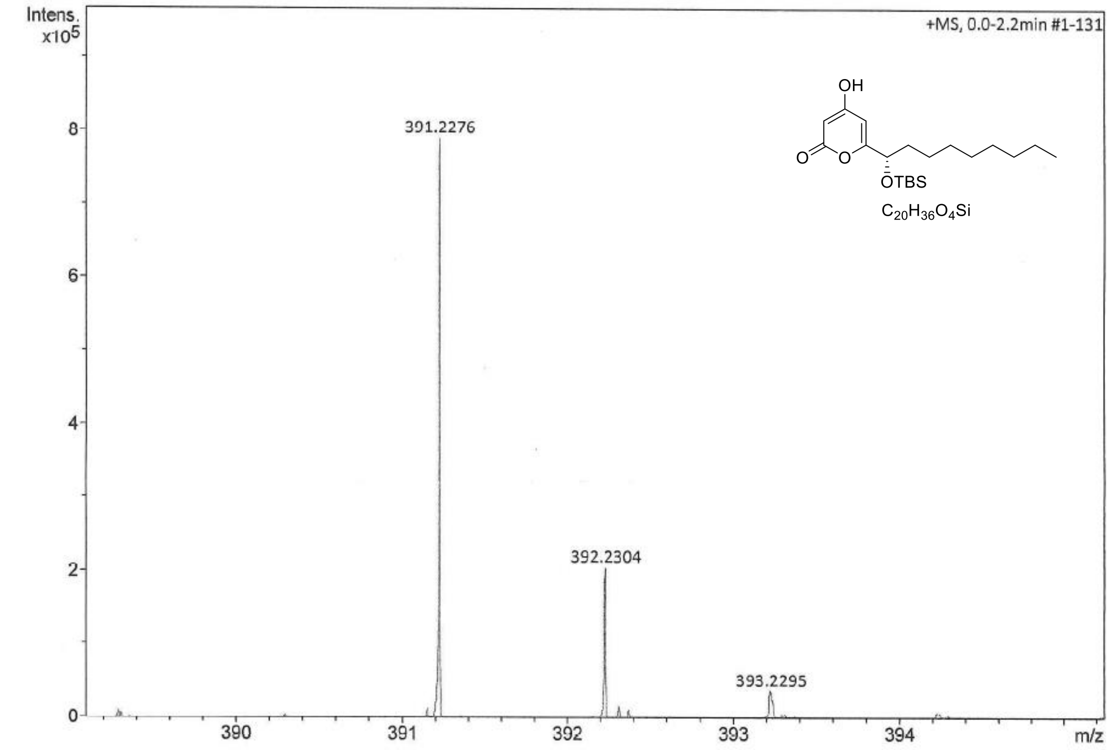


| Meas. m/z | Ion Formula | m/z | err [ppm] |
|---|---|---|---|
| 391.227648 | C18H31N6O2Si | 391.227227 | -1.1 |
| | C26H31O3 | 391.226771 | -2.2 |
| | C17H35N2O6Si | 391.225890 | -4.5 |
| | C20H36NaO4Si | 391.227507 | -0.4 |
| | C21H32N4NaSi | 391.228844 | 3.1 |

**Figure S-69** HRMS spectrum of compound **S7**.

**Acquisition Parameter**

| | | | | | |
|---|---|---|---|---|---|
| Source Type | ESI | Ion Polarity | Positive | Set Nebulizer | 0.4 Bar |
| Focus | Not active | Set Capillary | 3500 V | Set Dry Heater | 200 °C |
| Scan Begin | 100 m/z | Set End Plate Offset | -500 V | Set Dry Gas | 4.0 l/min |
| Scan End | 1500 m/z | Set Charging Voltage | 2000 V | Set Divert Valve | Waste |
| | | Set Corona | 0 nA | Set APCI Heater | 0 °C |

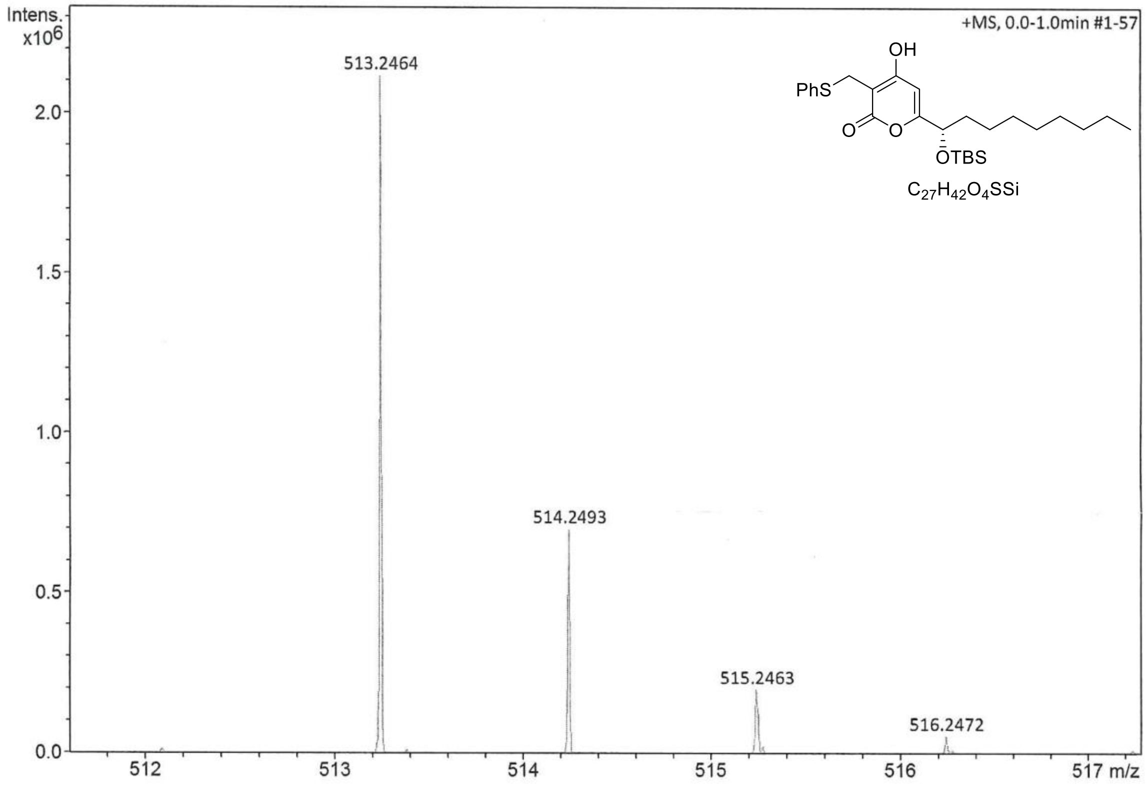


| Meas. m/z | Ion Formula | m/z | err [ppm] |
|---|---|---|---|
| 403.2275 | C20H31N6OS | 403.2275 | 0.0 |
| | C19H31N6O2Si | 403.2272 | -0.6 |
| | C22H36NaO3S | 403.2277 | 0.7 |
| | C21H36NaO4Si | 403.2275 | 0.1 |
| 513.2464 | C27H35N3O7 | 513.2470 | 1.0 |
| | C26H29N10O2 | 513.2469 | 1.0 |
| | C27H38NaO8 | 513.2459 | -1.1 |
| | C25H37N6O2SSi | 513.2462 | -0.4 |
| | C27H42NaO4SSi | 513.2465 | 0.2 |
| | C34H36NNaS | 513.2461 | -0.7 |
| 1003.5039 | C54H77N3O11SSi | 1003.5043 | 0.4 |
| | C60H71N4O8Si | 1003.5036 | -0.3 |
| | C62H70N5NaO4S | 1003.5041 | 0.2 |
| | C61H70N5NaO5Si | 1003.5038 | -0.0 |
| | C61H71N4O7S | 1003.5038 | -0.1 |
| | C59H65N11O3Si | 1003.5036 | -0.3 |
| | C60H65N11O2S | 1003.5038 | -0.1 |
| | C60H64N12NaSi | 1003.5038 | -0.0 |
| | C69H67N2O5 | 1003.5044 | 0.6 |

**Figure S-70** HRMS spectrum of compound **S8**.

**Acquisition Parameter**

| | | | | | |
|---|---|---|---|---|---|
| Source Type | ESI | Ion Polarity | Positive | Set Nebulizer | 0.4 Bar |
| Focus | Not active | Set Capillary | 3500 V | Set Dry Heater | 200 °C |
| Scan Begin | 100 m/z | Set End Plate Offset | -500 V | Set Dry Gas | 4.0 l/min |
| Scan End | 1500 m/z | Set Charging Voltage | 2000 V | Set Divert Valve | Waste |
| | | Set Corona | 0 nA | Set APCI Heater | 0 °C |

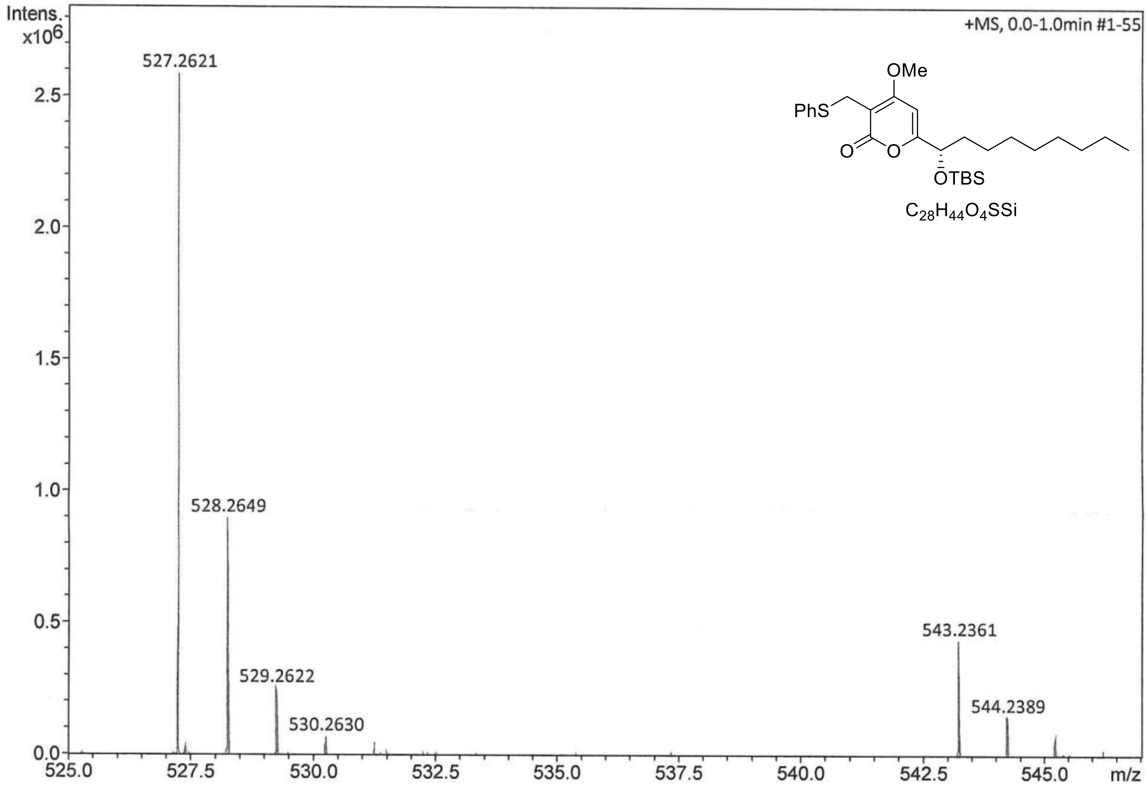


| Meas. m/z | Ion Formula | m/z | err [ppm] |
|---|---|---|---|
| 527.2621 | C34H39O3S | 527.2614 | -1.3 |
| | C29H36N4NaO4 | 527.2629 | 1.4 |
| | C28H37N3O7 | 527.2626 | 0.9 |
| | C27H31N10O2 | 527.2626 | 0.9 |
| | C26H39N6O2SSi | 527.2619 | -0.4 |
| | C35H38NNaS | 527.2617 | -0.8 |
| | C27H34N7NaO3 | 527.2615 | -1.1 |
| | C28H44NaO4SSi | 527.2622 | 0.1 |
| | C28H40NaO8 | 527.2615 | -1.1 |
| | C34H38NNaOSi | 527.2615 | -1.2 |
| | C34H35N4Si | 527.2626 | 0.8 |
| 543.2361 | C31H40NaO3SSi | 543.2360 | -0.2 |
| | C29H35N6OSSi | 543.2357 | -0.7 |
| | C24H36N6NaO5S | 543.2360 | -0.1 |
| | C31H33N3O6 | 543.2364 | 0.6 |
| | C32H32N4NaO3 | 543.2367 | 1.1 |
| | C30H30N7NaO2 | 543.2353 | -1.4 |
| | C30H27N10O | 543.2364 | 0.6 |
| | C37H34NNaSi | 543.2353 | -1.4 |

**Figure S-71** HRMS spectrum of compound **S9**.

**Acquisition Parameter**

| | | | | | |
|---|---|---|---|---|---|
| Source Type | ESI | Set Capillary | 3500 V | Set Nebulizer | 0.3 Bar |
| Focus | Not active | Set End Plate Offset | -500 V | Set Dry Heater | 200 °C |
| Scan Begin | 50 m/z | Set Charging Voltage | 2000 V | Set Dry Gas | 4.0 l/min |
| Scan End | 1500 m/z | Set Corona | 0 nA | Set Divert Valve | Waste |
| | | | | Set APCI Heater | 0 °C |

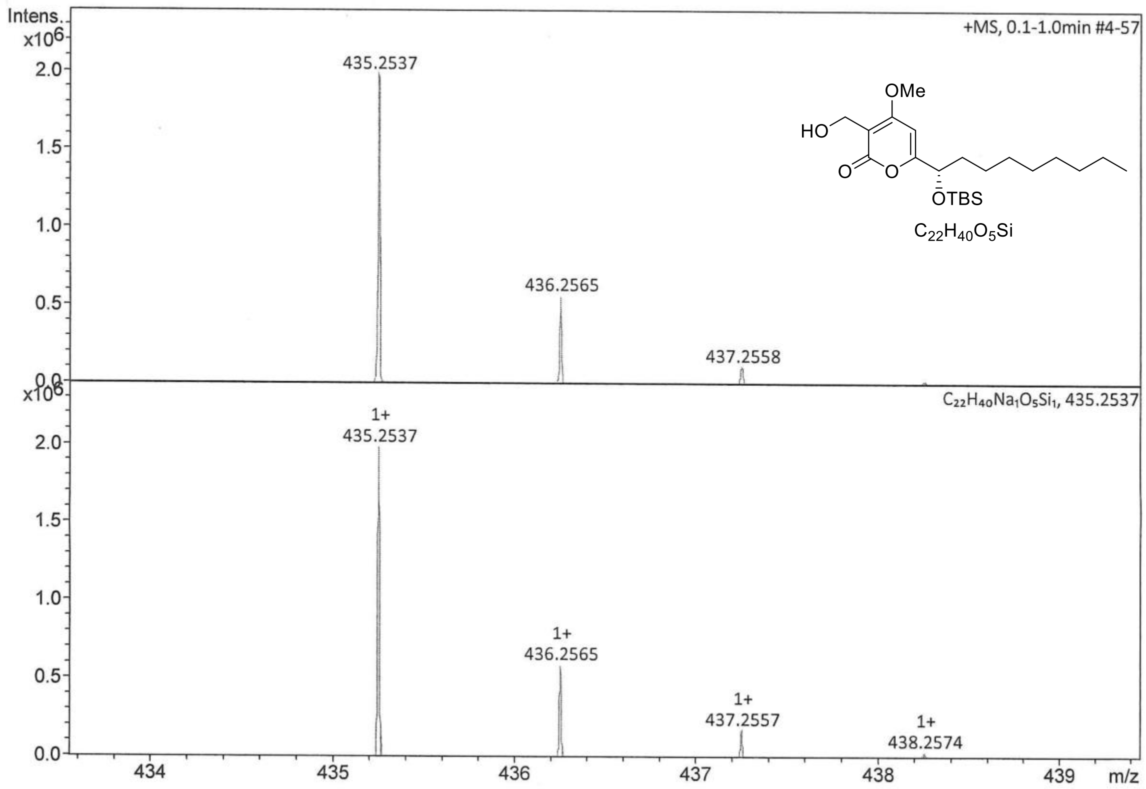


| Meas. m/z | Ion Formula | m/z | err [ppm] |
|---|---|---|---|
| 435.2537 | C28H35O4 | 435.2530 | -1.5 |
| | C20H35N6O3Si | 435.2534 | -0.5 |
| | C29H31N4 | 435.2543 | 1.5 |
| | C22H40NaO5Si | 435.2537 | 0.2 |

**Figure S-72** HRMS spectrum of compound **S11**.

## Acquisition Parameter

| | | | | | |
|---|---|---|---|---|---|
| Source Type | ESI | Set Capillary | 3500 V | Set Nebulizer | 0.3 Bar |
| Focus | Not active | Set End Plate Offset | -500 V | Set Dry Heater | 200 °C |
| Scan Begin | 50 m/z | Set Charging Voltage | 2000 V | Set Dry Gas | 4.0 l/min |
| Scan End | 1500 m/z | Set Corona | 0 nA | Set Divert Valve | Waste |
| | | | | Set APCI Heater | 0 °C |

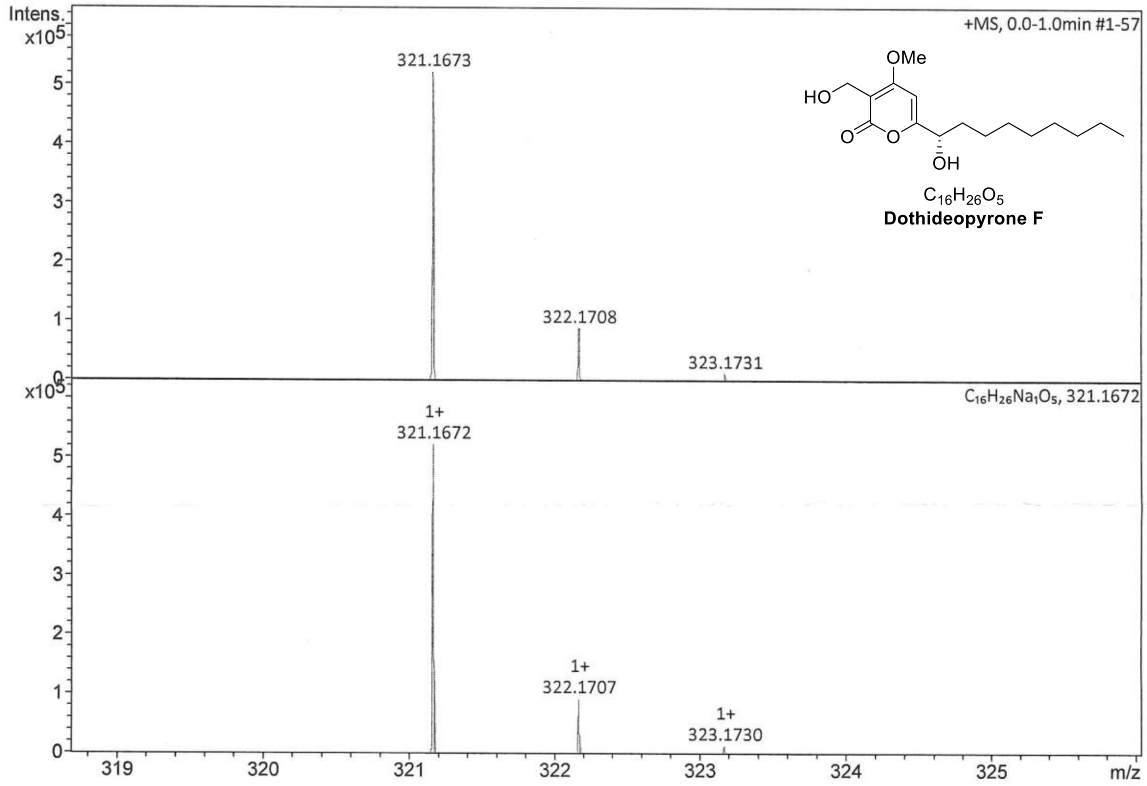


| Meas. m/z | Ion Formula | m/z | err [ppm] |
|---|---|---|---|
| 321.1673 | C14H21N6O3 | 321.1670 | -1.0 |
| | C13H25N2O7 | 321.1656 | -5.1 |
| | C16H26NaO5 | 321.1672 | -0.1 |
| | C17H22N4NaO | 321.1686 | 4.1 |

**Figure S-73** HRMS spectrum of dothideopyrone F (**2**).

## Acquisition Parameter

| | | | | | |
|---|---|---|---|---|---|
| Source Type | ESI | Ion Polarity | Positive | Set Nebulizer | 0.4 Bar |
| Focus | Active | Set Capillary | 3500 V | Set Dry Heater | 200 °C |
| Scan Begin | 50 m/z | Set End Plate Offset | -500 V | Set Dry Gas | 4.0 l/min |
| Scan End | 2000 m/z | Set Charging Voltage | 2000 V | Set Divert Valve | Waste |
| | | Set Corona | 0 nA | Set APCI Heater | 0 °C |

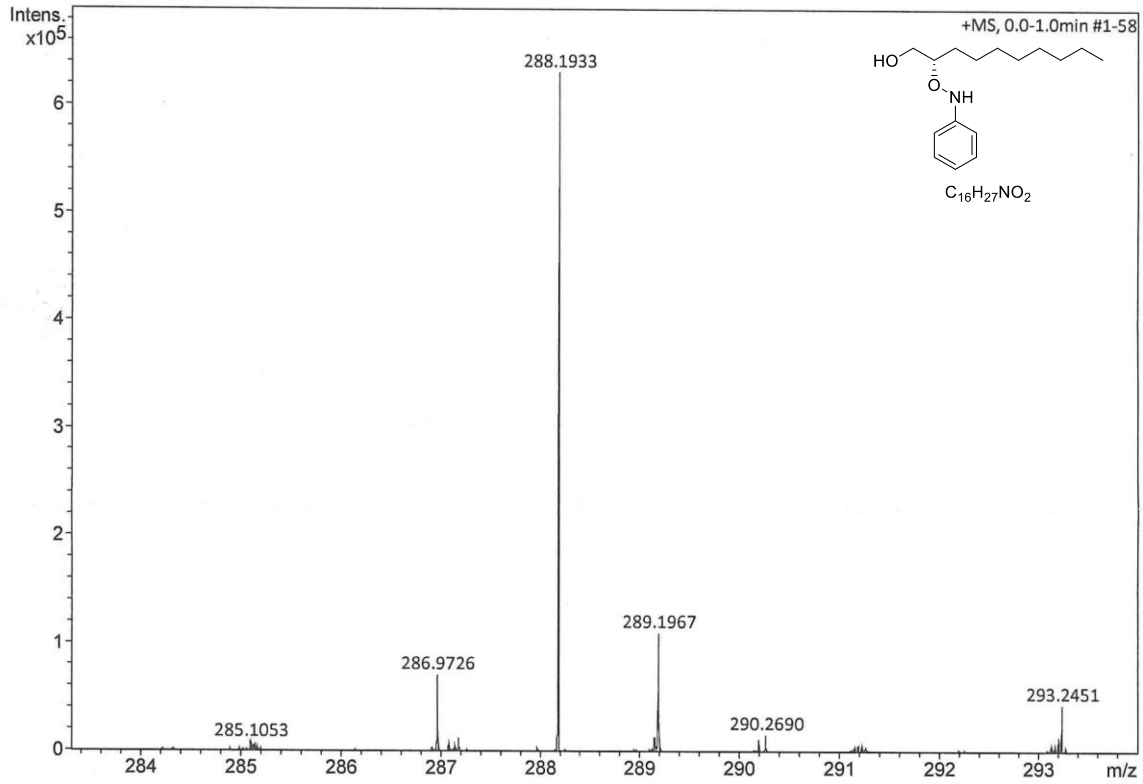


| Meas. m/z | Ion Formula | m/z | err [ppm] |
|---|---|---|---|
| 288.193346 | C14H22N7 | 288.193120 | -0.8 |
| | C16H27NNaO2 | 288.193400 | 0.2 |
| | C13H26N3O4 | 288.191783 | -5.4 |

**Figure S-74** HRMS spectrum of compound **S26b**.

# References:

(1) McGrath, M. J.; Fletcher, M. T.; König, W. A.; Moore, C. J.; Cribb, B. W.; Allsopp, P. G.; Kitching, W. *J. Org. Chem*., **2003**, *68*, 3739 and references therein.

(2) Kim, G. S.; Ko, W.; Kim, J. W.; Jeong, M.-H.; Ko, S.-K.; Hur, J.-S.; Oh, H.; Jang, J.-H.; Ahn, J. S. *J. Nat. Prod.*, **2018**, *81*, 1084.
